# New Synthetic Analogues of Natural Polyphenols as Sirtuin 1-Activating Compounds

**DOI:** 10.3390/ph15030339

**Published:** 2022-03-10

**Authors:** Giulia Bononi, Lorenzo Flori, Valentina Citi, Cecilia Acciai, Viviana Nocilla, Alma Martelli, Giulio Poli, Tiziano Tuccinardi, Carlotta Granchi, Lara Testai, Vincenzo Calderone, Filippo Minutolo

**Affiliations:** 1 Department of Pharmacy, University of Pisa, Via Bonanno 6, 56126 Pisa, Italy; giulia.bononi@phd.unipi.it (G.B.); lorenzo.flori@phd.unipi.it (L.F.); valentina.citi@unipi.it (V.C.); cecilia.acc@hotmail.it (C.A.); v.nocilla@studenti.unipi.it (V.N.); alma.martelli@unipi.it (A.M.); giulio.poli@unipi.it (G.P.); tiziano.tuccinardi@unipi.it (T.T.); vincenzo.calderone@unipi.it (V.C.); filippo.minutolo@unipi.it (F.M.); 2 Center for Instrument Sharing of the University of Pisa (CISUP), Lungarno Pacinotti 43, 56126 Pisa, Italy

**Keywords:** sirtuin 1, activators, I/R injury, cardioprotection, diarylamine, molecular modeling

## Abstract

NAD^+^-dependent deacetylase SIRT1 regulates many different biological processes, thus being involved in pathogenic conditions such as metabolic diseases, neurogenerative disorders and cancer. Notably, experimental evidence underlined that the activation of SIRT1 had promising cardioprotective effects. Consequently, many efforts have been so far devoted to finding new SIRT1 activators, both derived from natural sources or prepared by synthetic procedures. Herein, we discovered new SIRT1-activating derivatives, characterized by phenolic rings spaced by sulfur, nitrogen or oxygen-based central linkers. The newly synthesized derivatives were analyzed in enzymatic assays to determine their ability to activate SIRT1, as compared with that of resveratrol. Among the tested molecules, bisarylaniline compound **10** proved to be the most efficient SIRT1 activator. An evaluation of the effects caused by focused structural variations revealed that its *para*-hydroxy-substituted diphenyl moiety of **10** was the fundamental structural requirement for achieving good SIRT1 activation. Compound **10** was further investigated in ex vivo studies in isolated and perfused rat hearts submitted to ischemia/reperfusion (I/R), where it showed significant protection of the myocardium against I/R injury. Molecular modeling studies suggest the binding mode of **10** within SIRT1 in the presence of the p53-AMC peptide. Our findings reveal that this chemical scaffold may be used as the starting point to develop a new class of more potent SIRT1 activators as cardioprotective agents.

## 1. Introduction

**Figure 1:**
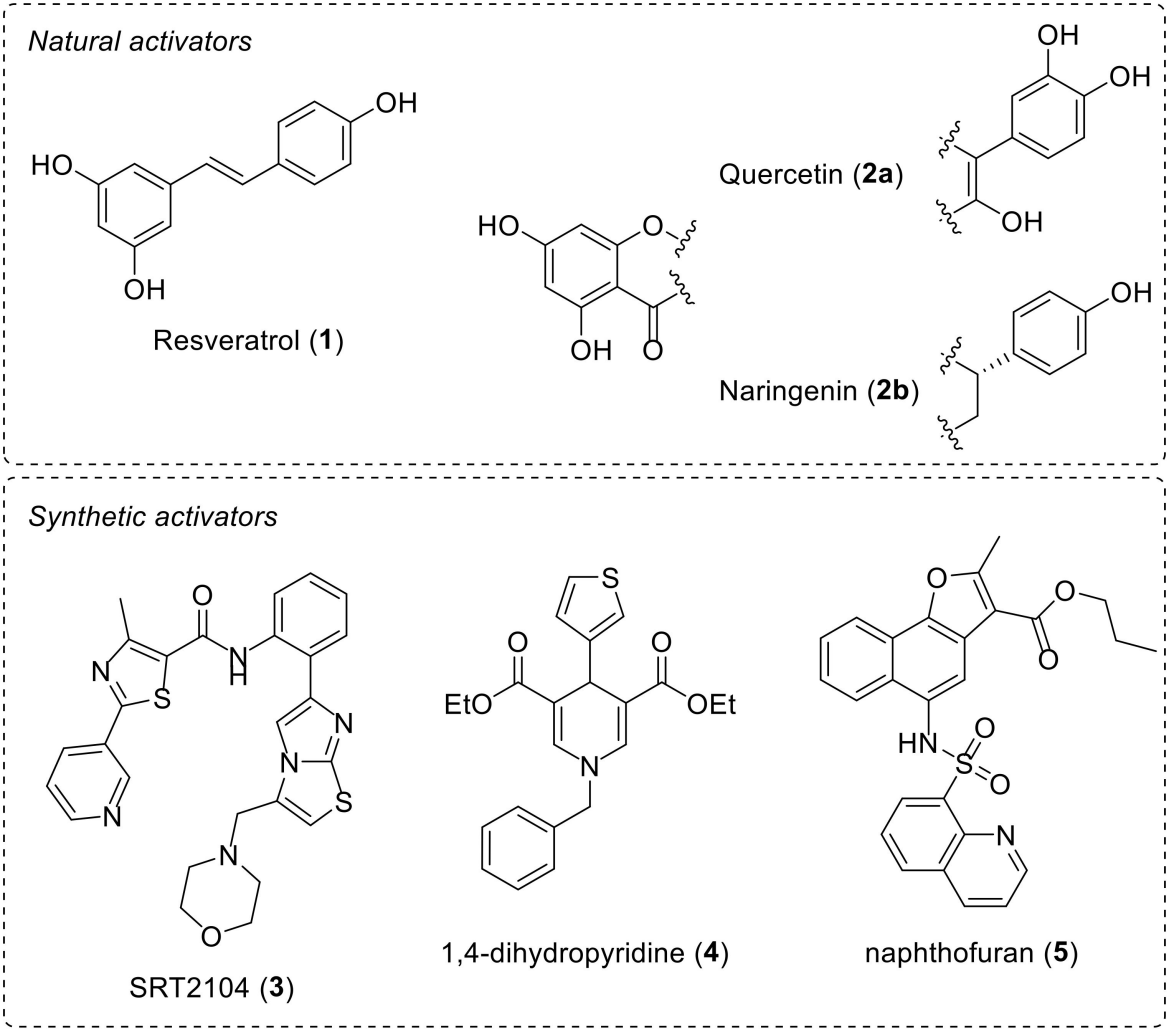
Chemical structures of some representative natural (**upper panel**) and synthetic (**lower panel**) SIRT1-activating compounds.

The highly conserved family of sirtuins (SIRTs) is composed of deacetylase enzymes, belonging to class III histone deacetylases, whose enzymatic activity is dependent on nicotinamide adenine dinucleotide (NAD^+^) as a cofactor [1]. SIRTs modulate many cellular physiological processes, such as gene expression, DNA repair, metabolism, oxidative stress defense and mitochondrial activity. There are seven members of this family, from SIRT1 to SIRT7, which differ in terms of cellular localization, tissue specificity and targets. SIRT1 is the most studied protein, and it is mainly expressed in the nucleus, but it is also present in the cytoplasm [2]. SIRT1 catalyzes the NAD^+^-dependent reversible deacetylation of ε-acetyl lysine side chains of target proteins, producing the deacetylated substrate, nicotinamide and 2′-*O*-acetyl-ADP-ribose, and it regulates different metabolic pathways, cell survival, cellular senescence and inflammation. Deregulation of these processes may lead to cancer, diabetes, cardiovascular diseases (i.e., myocardial ischemia/reperfusion injury) and age-related pathologies [3]. The key role of SIRT1 in the modulation of different transcription cofactors, which are essential for the maintenance of cellular homeostasis, makes this protein an appealing therapeutic target. For this reason, many efforts have been devoted to the development of SIRT1-activating compounds [4]. The most important SIRT1-activating compound of natural origin is resveratrol (*trans*-3,5,4′-trihydroxystilbene **1**, Figure 1), a polyphenolic compound isolated from red wine and grape skins. Resveratrol **1** not only activates SIRT1, but it also demonstrated to extend the lifespan of *Saccharomyces cerevisiae* and to play a cardioprotective role in several in vitro and in vivo models of ischemia/reperfusion injury [5]. Among other natural polyphenols, flavonoid quercetin **2a** (3,3′,4′,5,7-pentahydroxyflavone, Figure 1), typically present in fruits, vegetables and nuts, is able to activate SIRT1 and to protect endothelial cells from oxidative injuries [6]. Very recently, the flavonoid naringenin **2b** (4′,5,7-trihydroxyflavanone, Figure 1), characteristic of citrus fruits, including grapefruit and bergamot, has been also described as a SIRT1 activator and showed protective effects on myocardium against senescence-induced dysfunction [7]. In addition to natural SIRT1-activating compounds, many activators have been synthesized by both academic research groups and pharmaceutical companies, and one of the most important examples is the imidazothiazole derivative SRT2104 (**3**, Figure 1) developed by Sirtris Pharmaceuticals (a GSK company). SRT2104 displayed extensive biological and pharmacological effects mediated by SIRT1 activation and it was extensively investigated in clinical trials [8,9]. The research group of Prof. A. Mai developed a series of 1,4-dihydropyridine-based SIRT1-activating compounds exemplified by compound **4** (Figure 1), exerting a potent activation of SIRT1 [10]. Recently, a structure-based drug design strategy led to the identification of naphthofuran derivative **5** (Figure 1), which prevented apoptosis and inflammation by means of SIRT1 activation [11].

**Figure 2:**
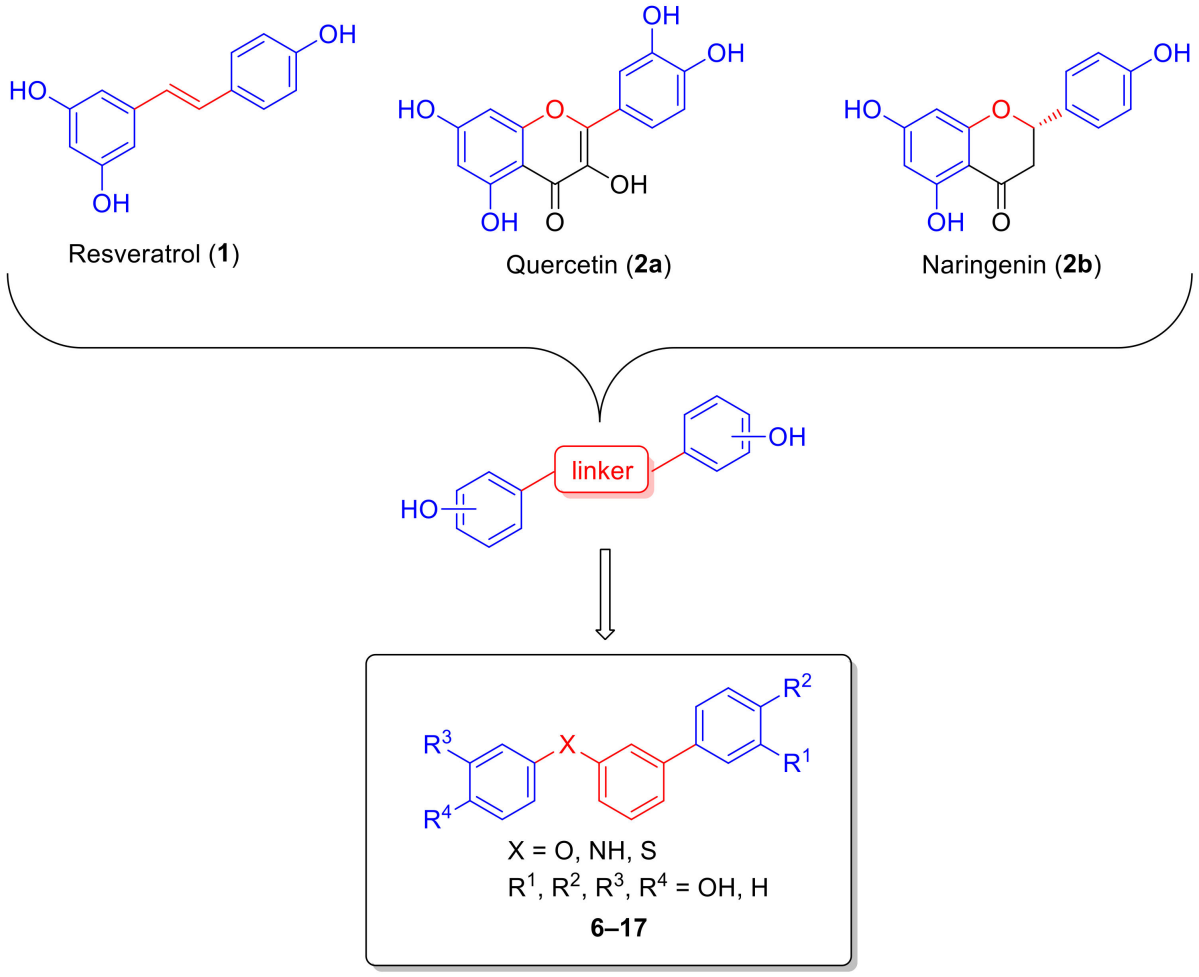
Design of new SIRT1-activating compounds. Structure of resveratrol **1**, quercetin **2a** and naringenin **2b** and schematic representation of their scaffold (phenolic rings in blue and linker portions in red); general structure of the new derivatives **6**–**17** (in the square).

A SAR analysis of the main natural SIRT1-activating compounds reported in literature discloses that the two phenolic rings of the *trans*-stilbene scaffold of resveratrol **1** are placed at the same distance as those of flavonoids quercetin **2a** and naringenin **2b**, since both structures are superimposable (Figure 2, superimposable moieties are highlighted in the same color). The only differences are: (a) the position of the hydroxyl groups in the phenyl rings and (b) the type of the linker between the two phenolic rings, which can be a simple two-atom alkene chain (in the case of **1**) or an ether portion, such as that of a pyranone cycle (in the case of **2a** and **2b**). This general structure, i.e., two phenolic rings linked by a portion containing heteroatoms and/or double bonds (Figure 2), prompted us to develop a new class of SIRT1-activating compounds possessing the general structure reported in Figure 2. The new derivatives **6**–**17** share two peripheral phenolic rings, with hydroxyl groups in *meta* and/or *para* position, which are linked by a central 1,3-disubstituted phenyl ring. The central benzene ring is directly connected to one phenolic ring, and it is linked to the other phenolic ring by means of a heteroatom, which can be an oxygen, a nitrogen or a sulfur atom.

## 2. Results and Discussion

### 2.1. Chemistry

**Scheme 1:**
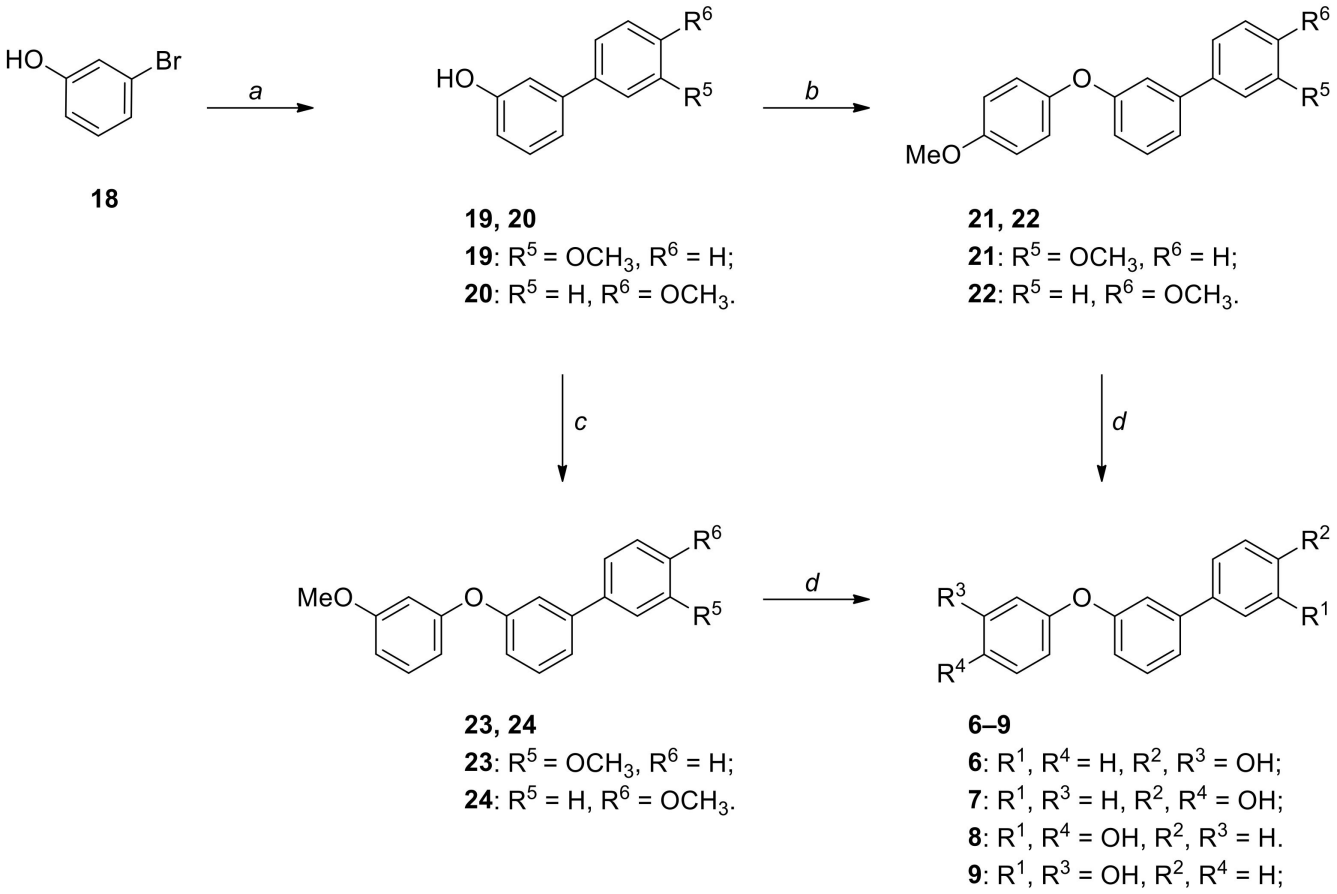
Synthesis of diarylether derivatives **6**–**9**. *Reagents and conditions:* (*a*) 3- or 4-methoxybenzeneboronic acid, Pd(OAc)_2_, PPh_3_, 2 M aq. Na_2_CO_3_, EtOH, toluene, 100 °C, 18 h [99%]; (*b*) 4-bromoanisole, CuI, K_3_PO_4_, anhydrous DMSO, 130 °C, 24 h [28–46%]; (*c*) 3-bromoanisole, Pd(OAc)_2_, Me_4_*t*BuXPhos, K_3_PO_4_, anhydrous toluene, 100 °C, 18 h [58–71%]; (*d*) 1 M BBr_3_ in CH_2_Cl_2_, anhydrous CH_2_Cl_2_, −15 to 0 °C, then RT, 3 h [71–95%].

The synthesis of derivatives **6**–**9**, characterized by the presence of an oxygen atom as the linker, started from commercially available 3-bromophenol **18**, which was subjected to a cross-coupling reaction under Pd(PPh_3_)_4_-catalyzed Suzuki conditions [12] with 3- or 4-methoxybenzeneboronic acid to obtain diaryl derivatives **19** or **20** (Scheme 1, step *a*). Intermediates **19** or **20** were reacted with 3- and 4-bromoanisole for the formation of a C–O bond by adopting different reaction conditions, according to the position of the methoxy group in the brominated derivative. In fact, they reacted in a ligand-free Ullmann-type reaction with 4-bromoanisole under Cu(I) catalysis, in the presence of potassium phosphate as the base and dimethylsulfoxide (DMSO) as the solvent, under prolonged heating [13], to yield 4-methoxy-substituted diarylether derivatives **21** and **22** (Scheme 1, step *b*). Differently, in the case of the coupling with 3-bromoanisole, the catalytic system consisted of palladium acetate as the catalyst and Me_4_*t*BuXPhos as the ligand, with potassium phosphate as the base in anhydrous toluene as the solvent [14] to yield 3-methoxy-susbstituted intermediates **23** and **24** (Scheme 1, step *c*). All the methoxy-substituted derivatives **21**–**24** were subjected to a common last synthetic step, consisting of a BBr_3_-promoted removal of the methoxy groups, to obtain the desired diarylether phenolic compounds **6**–**9** (Scheme 1, step *d*).

**Scheme 2:**
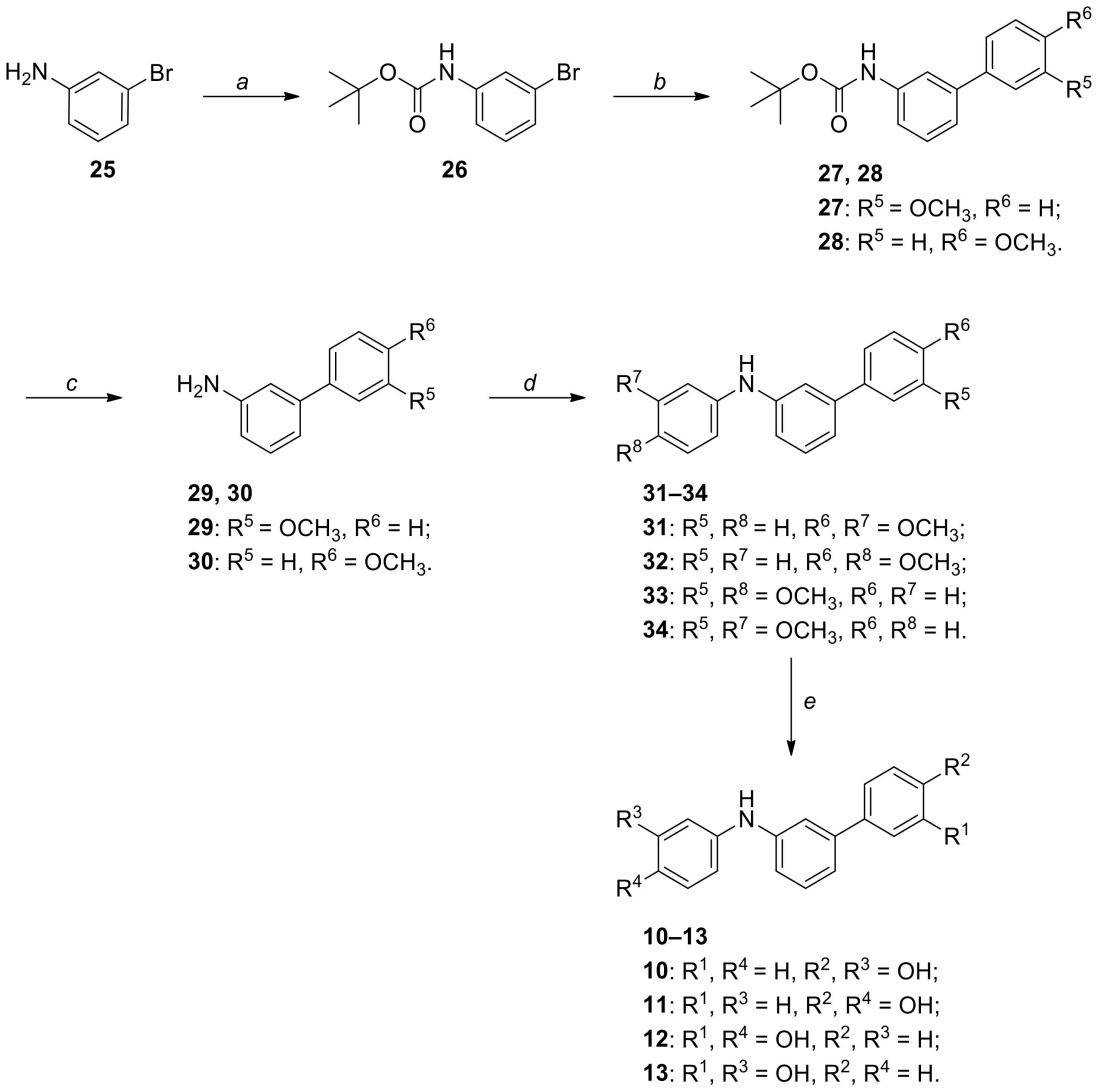
Synthesis of diarylamine derivatives **10**–**13**. *Reagents and conditions:* (*a*) (Boc)_2_O, Et_3_N, anhydrous THF, 0 °C to RT, 24 h [62%]; (*b*) 3- or 4-methoxybenzeneboronic acid, Pd(OAc)_2_, PPh_3_, 2 M aq. Na_2_CO_3_, EtOH, toluene, 100 °C, 18 h [89%]; (*c*) TFA, anhydrous CH_2_Cl_2_, 0 °C to RT, 2 h [85–94%]; (*d*) 3- or 4-bromoanisole, Pd_2_dba_3_, XPhos, K_3_PO_4_, anhydrous toluene, 100 °C, 20 h [39–91%]; (*e*) 1 M BBr_3_ in CH_2_Cl_2_, anhydrous CH_2_Cl_2_, −15 to 0 °C, then RT, 3 h [72–98%].

Diarylamine derivatives **10**–**13** were prepared from commercially available 3-bromoaniline **25**, but it was necessary to perform a precautionary Boc protection of the aniline moiety of **25** to obtain intermediate **26** (Scheme 2, step *a*). Boc-protected aryl bromide **26** was subjected to a cross-coupling reaction under Suzuki conditions, similarly to the previously described reaction of Scheme 1, to obtain diaryl intermediates **27** and **28** (Scheme 2, step *b*). Trifluoroacetic acid-mediated deprotection of the aniline moiety (Scheme 2, step *c*) was followed by Pd-catalyzed amination of 3- or 4-bromoanisole (Scheme 2, step *d*). The C–N bond-forming conditions required tris(dibenzylideneacetone)dipalladium as the catalyst, XPhos as the ligand, with potassium phosphate as the base in refluxing toluene [15]. Dimethoxy-substituted derivatives **31**–**34** were then transformed to the corresponding phenolic derivatives **10**–**13** by using a dichloromethane solution of boron tribromide (Scheme 2, step *e*).

**Scheme 3:**
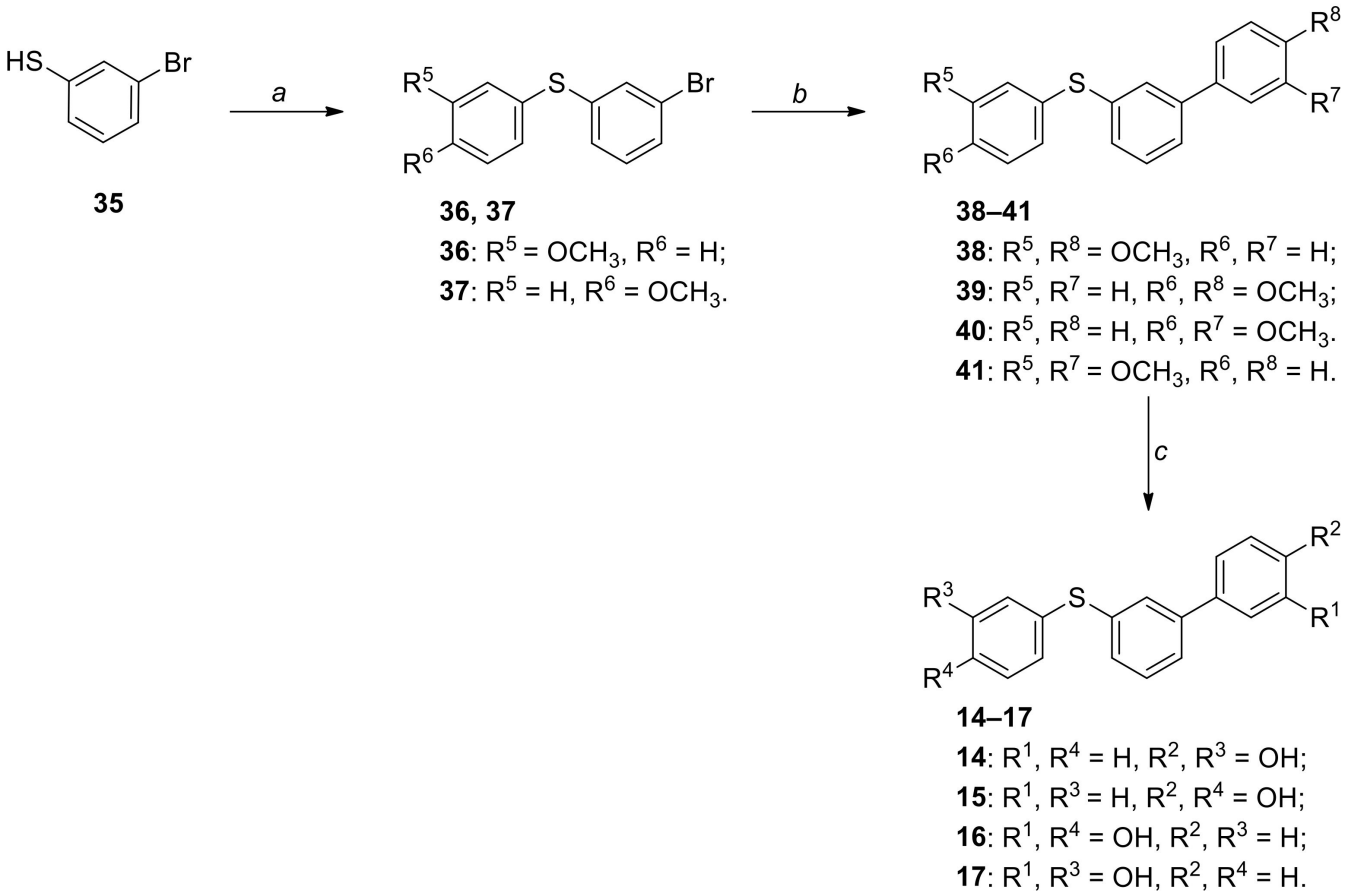
Synthesis of diarylsulphide derivatives **14**–**17**. *Reagents and conditions:* (*a*) 3- or 4-iodoanisole, CuI, K_2_CO_3_, ethylene glycol, *i*PrOH, 80 °C, 18 h [46–83%]; (*b*) 3- or 4-methoxybenzeneboronic acid, Pd(OAc)_2_, PPh_3_, 2 M aq. Na_2_CO_3_, EtOH, toluene, 100 °C, 18 h [61–91%]; (*c*) 1 M BBr_3_ in CH_2_Cl_2_, anhydrous CH_2_Cl_2_, −15 to 0 °C, then RT, 3 h [78–99%].

The formation of S-containing derivatives **14**–**17** followed a slightly different sequence of reactions: unlike the previous synthetic schemes, diarylsulphide derivatives were prepared from commercially available 3-bromothiophenol **35** (Scheme 3) and, in this case, it was chosen to first react the SH group of **35** with properly substituted iodoanisole derivatives, and later the aryl bromide intermediates were subjected to a C–C bond formation reaction. In detail, 3-bromothiophenol **35** and 3- or 4-iodoanisole reacted in an Ullmann-type reaction thanks to copper iodide-mediated catalysis (Scheme 3, step *a*), with potassium carbonate as the base, in an ethylene glycol–isopropanol mixture of solvents [16].

Diaryl sulfide derivatives **36** and **37** were subsequently subjected to a cross-coupling reaction with 3- or 4-methoxybenzeneboronic acid, yielding derivatives **38**–**41** (Scheme 3, step *b*). The last reaction of BBr_3_-mediated deprotection of the methoxy groups allowed the obtainment of final phenolic compounds **14**–**17** (Scheme 3, step *c*).

All the final compounds **6**–**17**, as well as intermediates **31** and **32**, which were submitted to biological assays, were characterized by a purity of ≥95%, as detected by HPLC analysis.

### 2.2. Enzymatic Assays

The newly synthesized compounds were tested for their ability to activate SIRT1 at the concentration of 100 µM, by using resveratrol as a positive control (Table 1). The basal value of fluorescence recorded in the presence of the vehicle (DMSO 0.1%) was considered as 100%. Then, the effects of the newly synthetized compounds, as well as of the reference compound resveratrol, were evaluated as %SIRT1 activation and compared to the basal value.

Unfortunately, diarylether derivatives **6**–**9** were not able to activate SIRT1, and similarly, compounds **14**–**17**, possessing a sulfur atom as the linker, were inefficient in inducing an activating effect on the target. The best results were yielded from bisarylaniline derivatives **10**–**13**, although their effects were different according to the substitution pattern. Compounds **10** and **11**, bearing a para-hydroxy-substituted diphenyl moiety, activated SIRT1, displaying a good activation ability (136% and 133% for **10** and **11**, respectively), albeit milder than that of resveratrol (174% activation). Conversely, the presence of a meta-OH-diphenyl portion, as in compounds **12** and **13**, was detrimental for the activating ability of this series of diarylamine derivatives, since compounds **12** and **13** did not activate SIRT1. In order to confirm that the presence of phenolic groups is necessary for the biological activity on SIRT1, the methoxylated analogues of the most promising compounds **10** and **11**, intermediates **31** and **32** (Scheme 2), were tested in the same assay conditions. Methoxy-substituted derivatives **31** and **32** were unable to activate SIRT1, thus confirming that the presence of para-hydroxy-substituted diphenyl moiety is necessary to confer activating abilities to the compounds.

### 2.3. Anti-Ischemic Activity of Compound **10** in an Ex Vivo Model of I/R

**Figure 3:**
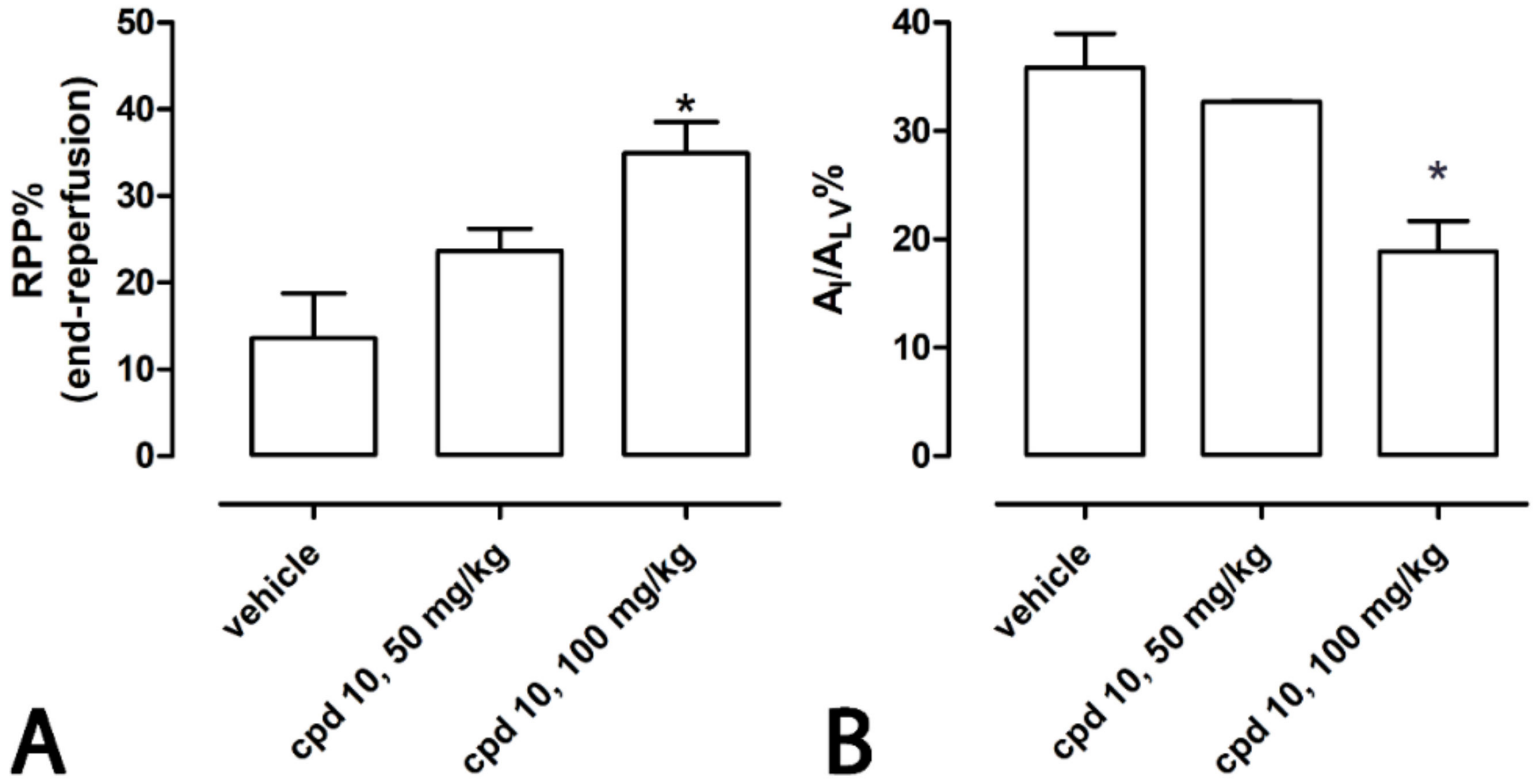
The histograms represent the functional and morphological changes induced by treatment of the animals with compound **10** or with vehicle before I/R episode. (**A**) Changes in RPP% at the end of reperfusion; (**B**) changes in the percentage of ischemic area vs. left ventricle area (A_I_/A_LV_%). The vertical bars symbolize the standard errors (*n* = 6). Asterisks show a statistically significant difference from the value observed in the hearts of vehicle-treated animals (* *p* < 0.05).

It is well-known that SIRT1 activators are able to produce a broad spectrum of benefits on the organism, such as regulation of the cell metabolism, of inflammatory processes and antioxidant defenses; moreover, although this enzyme is located in cytoplasm, its activity has significant effects on mitochondrial homeostasis. For this reason, SIRT1 enzyme has been implicated in several cardiovascular diseases, among which the protection of myocardium against the damage induced by exposition at ischemia/reperfusion (I/R) periods [4,17,18,19]. Therefore, in this work, we evaluated the functional efficacy of compound **10** in a heart isolated and perfused on Langendorff apparatus submitted to I/R protocol, which represents a well-known model to reproduce myocardial damage induced by ischemic events. In our experimental conditions, an I/R episode produced marked damage to the isolated hearts of vehicle-treated rats. In this regard, a marked decrease of the functional parameters of myocardial contractile function (RPP) was observed during the reperfusion time and at the end, the value of RPP was 14 ± 5%, compared to the pre-ischemic period (Figure 3A). The damage induced by the I/R event was also visible through morphometric analysis, and a size of the ischemic area equal to 36 ± 3% was measured using formazan salt (Figure 3B).

The treatment with compound **10** at the lowest dosage (50 mg/kg) did not significantly improve functional and morphometric parameters. Indeed, RPP value was 28 ± 3% at the end of reperfusion and A_I_/A_LV_% reached the value of 33 ± 1%. Very interestingly, compound **10** at 100 mg/kg significantly showed a protection of myocardial tissue against I/R injury. In particular, at the end of the reperfusion period, the RPP value was significantly increased (33 ± 4%) and consistently a reduced degree of damage (A_I_/A_LV_% = 19 ± 5%, Figure 3) was observed. Taken together, these results unequivocally demonstrate, accordingly to the SIRT1 activators profile, the cardioprotective activity of compound **10** against I/R injury.

### 2.4. Molecular Modeling Studies

**Figure 4:**
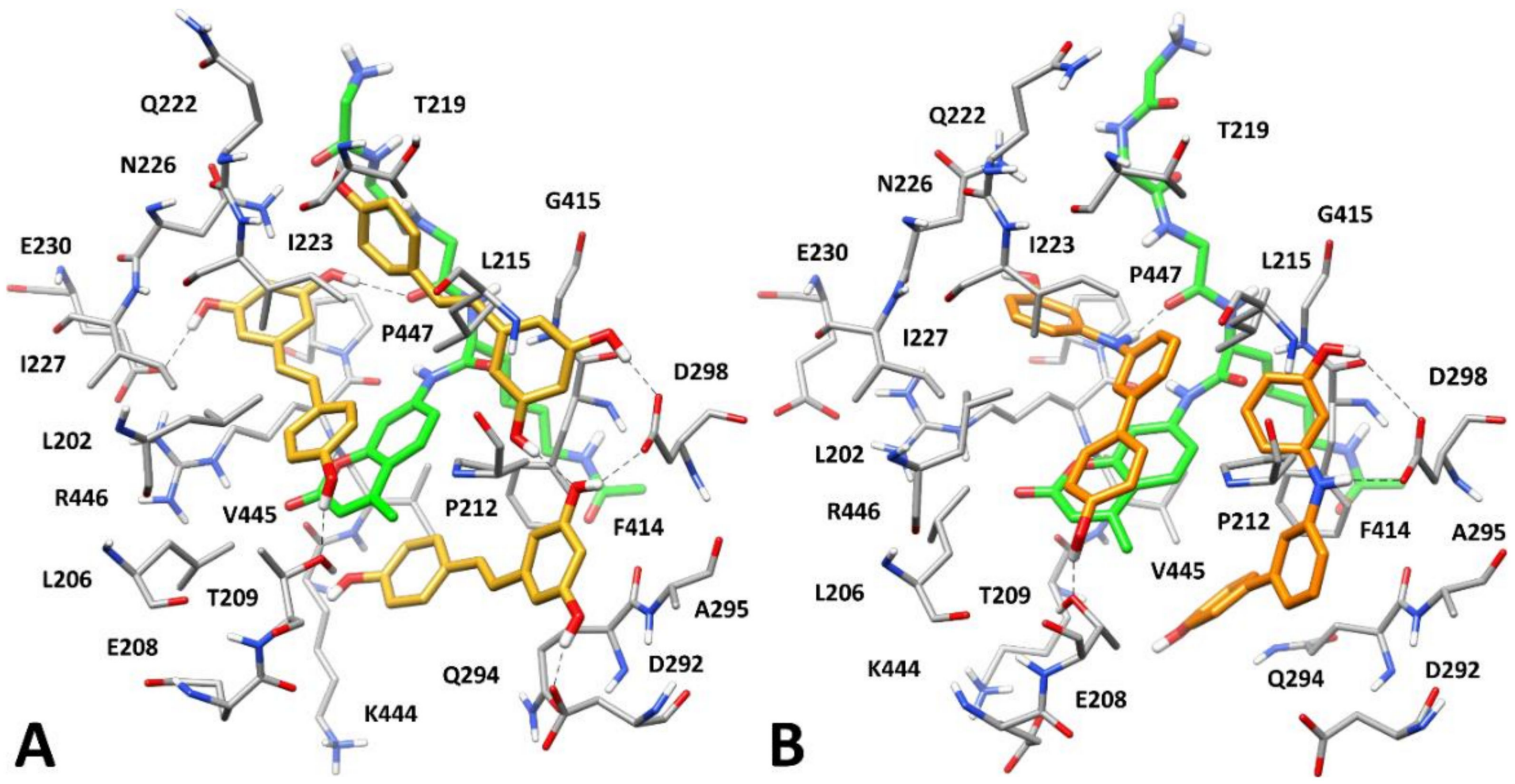
(**A**) Minimized average structure of SIRT1 in complex with p53-AMC peptide (green) and three molecules of resveratrol (gold). (**B**) Minimized average structure of SIRT1 in complex with p53-AMC peptide and two molecules of **10** (orange). In both panels, ligand–protein and ligand–peptide H-bonds are highlighted as black dashed lines. For clarity, the side chains of R1, H2 and K3 of the p53-AMC peptide are not shown.

In order to evaluate the potential binding disposition of the phenolic derivatives within SIRT1 in presence of the p53-7-amino-4-methylcoumarin (AMC) peptide, we performed molecular docking and molecular dynamics (MD) simulation studies using the X-ray structure of SIRT1 in complex with p53-AMC peptide and resveratrol (PDB code 5BTR [20]) as a reference. Since three resveratrol molecules are bound to the SIRT1-p53-AMC complex in the reference X-ray structure (Figure 4A), we initially validated our docking/MD protocol. Self-docking studies were performed using GOLD software, with the ChemScore fitness function, in all three receptor sub-sites in which resveratrol is located. The docking procedure successfully reproduced all three binding conformations, with RMSD values between each top-scored docking pose and the corresponding experimental disposition below 2.0 Å (see Materials and Methods for details). We then evaluated the reliability of a robust MD simulation protocol in explicit solvent composed of a multistep heating/equilibration stage followed by 500 ns of MD production, which was applied to the reference X-ray complex. As shown in Figure S29, the binding disposition of both the fluorogenic peptide and all resveratrol molecules was maintained during the MD. In particular, the AMC moiety of the peptide showed an average RMSD of its disposition during the MD of 0.7 Å, with respect to the experimental coordinates; similarly, the average RMSD of the three resveratrol molecules ranged between 0.7 and 1.1 Å, thus confirming the reliability of the MD protocol. Figure 4A shows the minimized average structure of SIRT1 in complex with the p53-AMC peptide and three molecules of resveratrol, obtained from the last 250 ns of MD simulation, which highlights the main interactions formed by the ligands observed during the MD. The resveratrol molecule that shows the greatest number of interactions with both the protein and the p53-AMC peptide is buried within a cavity, which will be herein called site 1, formed by the SIRT1 catalytic site, the backbone of the fluorogenic peptide and its AMC moiety. The molecule is firmly anchored to its binding site through three different H-bonds: one established with the backbone oxygen of the p53-AMC peptide, a second one formed with the carboxylic group of E230 and a last one with T209. Interestingly, this latter H-bond was not observed in the experimental X-ray complex, but was rapidly formed during the MD and maintained for about 80% of the simulation. Moreover, the ligand shows extensive π–π stacking interactions with the AMC moiety of the peptide, as well as numerous hydrophobic contacts with L202, I223, I227 and the side chains of R446 and P447. The other two resveratrol molecules are located in more solvent-exposed regions of the protein catalytic site. The molecule placed in what we refer to as site 2 forms two H-bonds with the carboxylic groups of D292 and D298, shows a π–π stacking with the AMC portion of the peptide and establishes lipophilic interactions with the side chains of A295, F414, K444 and V445. Moreover, an additional H-bond with the third resveratrol molecule is observed. In fact, the ligand placed in what we call site 3 shows a network of H-bonds with D298 and the molecule in site 2, and it forms a series of hydrophobic contacts with the p53-AMC peptide, the ligand molecule placed in site 1 and a series of protein residues, i.e., P212, L215, T219, I223 and G415 (Figure 4A).

The same validated docking/MD protocol was then employed for evaluating the binding mode of compound **10**, the most potent SIRT1 agonist among the series of synthesized phenolic derivatives. Due to the bigger size of compound **10** with respect to resveratrol, the top-scored binding pose obtained by docking the ligand within site 2 and site 3 converged into a single binding conformation occupying site 2 and part of site 3. Therefore, the final corresponding SIRT1-peptide-**10** complex predicted by docking included only two ligand molecules. Figure 4B shows the average structure of the refined quaternary complex obtained through MD simulation studies. The compound located in site 1 forms most of the interactions identified for the corresponding resveratrol molecule in the reference X-ray structure. In particular, we observe an H-bond between the diaryl amine group of the ligand and the backbone of the p53-AMC peptide that is maintained for the whole simulation, as well as an H-bond with T209 observed for most of the MD. Moreover, the ligand shows π–π stacking interactions with both AMC rings and hydrophobic interactions predominantly with L206, P212, L215 and I223 through the biphenyl moiety, as well as with N226, I227, R446 and P447 through the meta-phenol ring. The second ligand molecule mimics resveratrol’s interactions with the carboxylic group of D298, forming a strong H-bond through its NH group and a second, less stable H-bond established through the meta-hydroxyl group. Interestingly, the meta-phenol ring of the ligand is locked in its disposition thanks to the molecule placed in site 1, which creates a sort of pocket together with P212, F414 and G415 (which form hydrophobic interactions with the ligand in site 2), as well as D298. This reciprocal disposition of the two ligand molecules clearly contributes to the stability of the H-bonds with D298. Moreover, the molecule shows both T-shaped and parallel-displaced π–π stacking with F414 formed through its biphenyl moiety, as well as additional hydrophobic interactions with L215, Q294, A295 and V445. In conclusion, the two molecules of compound **10** nicely fit within the three SIRT1 subsites to which resveratrol is bound in the reference X-ray complex. Although compound **10** does not fully occupy site 3, it succeeds in establishing most of its interactions with the enzyme and the p53-AMC peptide observed for resveratrol, maintaining a stable binding conformation (with an average RMSD during the simulation between 1.1 and 1.5 Å), which is consistent with the remarkable agonist activity of this class of molecules.

**Figure 5:**
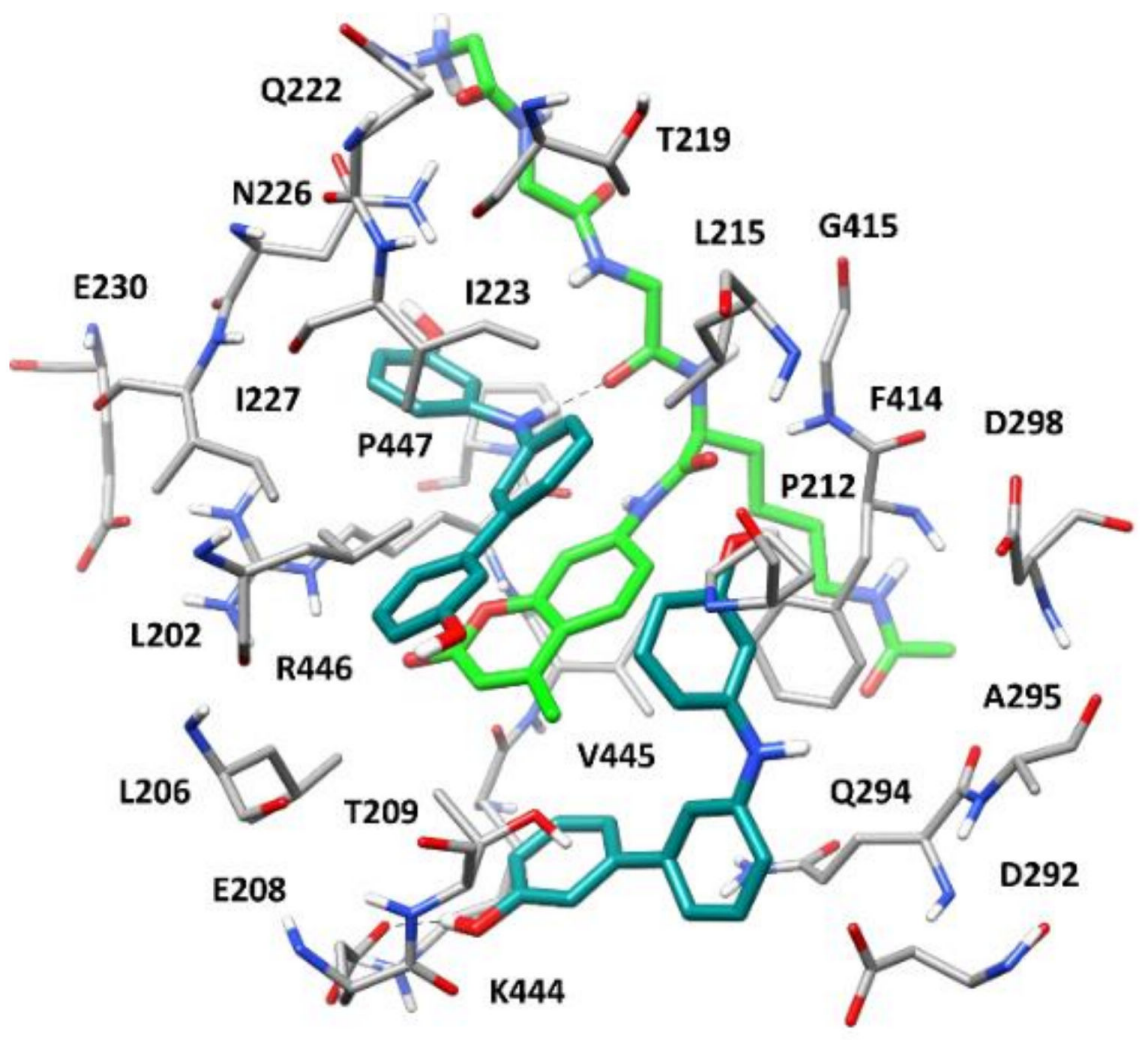
Minimized average structure of SIRT1 in complex with p53-AMC peptide (green) and two molecules of **13** (dark cyan). Ligand–protein and ligand–peptide H-bonds are highlighted as black dashed lines. For clarity, the side chains of R1, H2 and K3 of the of p53-AMC peptide are not shown.

The binding mode predicted for compound **10** within the quaternary complex with SIRT1 and the p53-AMC peptide is in agreement with the main SAR data derived from the biological evaluation of the compound series, highlighting the importance of an H-bond donor group as a linker between the phenol and the biphenyl fragments of the compounds for maintaining the activity. In fact, all diaryl ethers and sulfides, which would be unable to form the key interactions with D298 and the backbone of the p53-AMC peptide established by the NH group of compound **10**, appeared to be devoid of any activation activity. Moreover, moving the OH group in the biphenyl portion of the ligand from the para position (as in compound **10**) to the meta position proved to be highly detrimental for the activity, since compound **13** appeared to be totally inactive. In order to better evaluate the effect of this structural modification on the ligands’ disposition, the binding mode of compound **13** was studied using the same docking/MD protocol employed for compound **10**. As shown in Figure 5, the ligand molecule placed within site 1 shows a binding mode in which the H-bond between its NH group and the backbone oxygen of the p53-AMC peptide is maintained, as well as the disposition of the adjacent phenol fragment, which forms hydrophobic interactions with N226, I227, R446 and P447. However, the ligand is not able to form the key H-bond with T209, also observed for resveratrol; this leads to a shift in the disposition of the biphenyl moiety, which moves toward L206 and determines a rotation of the side chain of this residue. As a consequence of this, the second molecule of compound **13**, occupying site 2, is not locked anymore among P212, D298, F414 and G415 (as observed for compound **10**) and it moves towards the solvent, further away from these residues, losing both the H-bonds with D298 and the π–π stacking interactions with F414, being only able to form a transient interaction with E208, which is involved in a salt bridge with K444. In total, we observed a less stable ligand binding mode (with an average RMSD during the simulation between 1.5 and 2.2 Å) characterized by the disruption of key H-bonds, also formed by resveratrol in the reference complex, as well as the loss of the multiple aromatic and hydrophobic interactions predicted for compound **10**, which may justify the inactivity of **13**. Moreover, based on these results, we could envision that the methylation of both hydroxyl groups of compound **10** would cause a similar, or even worse, deleterious effect on the ligand binding mode, thus preventing the formation of the H-bonds with the key anchoring residues of SIRT1, which could be at the basis of the inactivity of the methoxylated derivatives **31** and **32**.

## 3. Materials and Methods

### 3.1. Synthesis. General Procedures and Materials

All solvents and chemicals were used as purchased without further purification. Chromatographic separations were performed on silica gel columns by flash chromatography (Kieselgel 40, 0.040−0.063 mm; Merck KGaA, Darmstadt, Germany). Reactions were followed by thin-layer chromatography (TLC) on Merck aluminum silica gel (60 F254) sheets that were visualized under a UV lamp. Evaporation was performed in vacuo (rotating evaporator). Sodium sulfate was always used as the drying agent. Proton (^1^H) and carbon (^13^C) NMR spectra were obtained with a Bruker Avance III 400 MHz spectrometer using the indicated deuterated solvents (Figures S15–S28). Chemical shifts are given in parts per million (ppm) (δ relative to residual solvent peak for ^1^H and ^13^C). ^1^H-NMR spectra are reported in this order: multiplicity and number of protons. Standard abbreviation indicating the multiplicity were used as follows: s = singlet, dd = doublet of doublets, ddd = doublet of doublet of doublets, t = triplet, dt = doublet of triplets, td = triplet of doublets, m = multiplet and bs = broad singlet. HPLC analysis was used to determine purity (Figures S1–S14): all target compounds (i.e., assessed in biological assays) were ≥95% pure by HPLC, as confirmed via UV detection (λ = 254 nm). Analytical reverse-phase HPLC was conducted using a Kinetex EVO C18 column (5 μm, 150 × 4.6 mm, Phenomenex, Inc., Torrance, CA, United States); eluent A, water; eluent B, CH_3_CN; after 5 min at 25% B, a gradient was formed from 25% to 75% of B in 5 min and held at 75% of B for 10 min; flow rate was 1 mL/min. HPLC analyses were performed at 254 nm. The ESI-MS spectra were recorded by direct injection at 5 μL min^−1^ flow rate in an Orbitrap high-resolution mass spectrometer (Thermo, San Jose, CA, USA), equipped with HESI source (Figures S30–S43). The working conditions were as follows: positive polarity, spray voltage 3.4 kV, capillary temperature 290 °C, S-lens RF level 50, the sheath gas was set at 24 and the auxiliary gas was set at 5 (arbitrary units). The conditions included negative polarity, spray voltage of 3.2 kV, capillary temperature at 290 °C, S-lens RF level 50; the sheath gas was set at 28 and the auxiliary gas was set at 4 (arbitrary units). For acquisition and analysis, Xcalibur 4.2 software (Thermo) was used. For spectrum acquisition, a nominal resolution (at *m*/*z* 200) of 140,000 was used. Yields refer to isolated and purified products derived from nonoptimized procedures.

#### 3.1.1. General Procedure for the Formation of Compounds **19**, **20**, **27**, **28**, **38**–**41**

A solution of Pd(OAc)_2_ (0.0435 mmol) and triphenylphosphine (0.218 mmol) in ethanol (3.3 mL) and toluene (3.3 mL) was stirred at room temperature under inert atmosphere for 10 min. After that period, commercially available 3-bromophenol **18** or intermediate **26** or intermediates **36** or **37** (1.45 mmol, 250 mg), a 2 M aqueous solution of Na_2_CO_3_ (3.3 mL) and commercially available 3- or 4-methoxybenzeneboronic acid (2.32 mmol) were sequentially added. The resulting mixture was heated at 100 °C in a sealed vial overnight. After being cooled to room temperature, the mixture was diluted with water and extracted with EtOAc. The combined organic phase was dried over anhydrous sodium sulphate and concentrated under vacuum. The crude product was purified by flash column chromatography, eluting with n-hexane/EtOAc mixtures as eluents afforded the biaryl intermediates **19, 20, 27, 28, 38**–**41**.

3′-Methoxy-[1,1′-biphenyl]-3-ol (**19**); 99% yield. ^1^H-NMR (CDCl_3_) δ (ppm): 3.87 (s, 3H), 6.83 (ddd, 1H, J = 8.0, 2.6, 1.0 Hz), 6.90 (ddd, 1H, J = 8.2, 2.6, 0.9 Hz), 7.06 (t, 1H, J = 2.0 Hz), 7.11 (t, 1H, J = 2.1 Hz), 7.14–7.16 (m, 1H), 7.16–7.18 (m, 1H), 7.31 (t, 1H, J = 7.9 Hz), 7.35 (t, 1H, J = 8.0 Hz).

4′-Methoxy-[1,1′-biphenyl]-3-ol (**20**); 99% yield. ^1^H-NMR (CDCl_3_) δ (ppm): 3.85 (s, 3H), 4.73–4.93 (bs, 1H), 6.78 (ddd, 1H, J = 8.1, 2.5, 0.9 Hz), 6.97 (AA’XX’, 2H, J_AX_ = 8.9 Hz, J_AA’/XX’_ = 2.6 Hz), 7.02 (dd, 1H, J = 2.2, 1.7 Hz), 7.13 (ddd, 1H, J = 7.7, 1.7, 1.0 Hz), 7.29 (t, 1H, J = 8.0 Hz), 7.51 (AA’XX’, 2H, J_AX_ = 8.9 Hz, J_AA’/XX’_ = 2.6 Hz).

tert-Butyl (3′-methoxy-[1,1′-biphenyl]-3-yl)carbamate (**27**); 89% yield. ^1^H-NMR (CDCl_3_) δ (ppm): 1.53 (s, 9H), 3.86 (s, 3H), 6.53 (bs, 1H), 6.89 (ddd, 1H, J = 8.2, 2.6, 0.9 Hz), 7.11 (t, 1H, J = 2.1 Hz), 7.17 (ddd, 1H, J = 7.7, 1.6, 1.0 Hz), 7.23–7.28 (m, 1H), 7.31–7.28 (m, 3H), 7.59 (s, 1H).

tert-Butyl (4′-methoxy-[1,1′-biphenyl]-3-yl)carbamate (**28**); 89% yield. ^1^H-NMR (CDCl_3_) δ (ppm): 1.53 (s, 9H), 3.85 (s, 3H), 6.51 (bs, 1H), 6.96 (AA’XX’, 2H, J_AX_ = 8.9 Hz, J_AA’/XX’_ = 2.6 Hz), 7.22 (dt, 1H, J = 7.4, 1.5 Hz), 7.27–7.35 (m, 2H), 7.52 (AA’XX’, 2H, J_AX_ = 8.9 Hz, J_AA’/XX’_ = 2.6 Hz), 7.59 (s, 1H).

(4′-Methoxy-[1,1′-biphenyl]-3-yl)(3-methoxyphenyl)sulfane (**38**); 71% yield. ^1^H-NMR (CDCl_3_) δ (ppm): 3.76 (s, 3H), 3.84 (s, 3H), 6.79 (ddd, 1H, J = 8.3, 2.5, 0.9 Hz), 6.91 (t, 1H, J = 2.1 Hz), 6.93–6.98 (m, 3H), 7.21 (t, 1H, J = 8.0 Hz), 7.29 (dt, 1H, J = 8.0, 1.5 Hz), 7.35 (t, 1H, J = 7.7 Hz), 7.44 (dt, 1H, J = 8.0, 1.5 Hz), 7.48 (AA’XX’, 2H, J_AX_ = 8.9 Hz, J_AA’/XX’_ = 2.6 Hz), 7.58 (t, 1H, J = 1.6 Hz).

(4′-Methoxy-[1,1′-biphenyl]-3-yl)(4-methoxyphenyl)sulfane (**39**); 91% yield. ^1^H-NMR (CDCl_3_) δ (ppm): 3.83 (s, 3H), 3.84 (s, 3H), 6.91 (AA’XX’, 2H, J_AX_ = 8.9 Hz, J_AA’/XX’_ = 2.6 Hz), 6.95 (AA’XX’, 2H, J_AX_ = 8.9 Hz, J_AA’/XX’_ = 2.6 Hz), 7.09 (ddd, 1H, J = 7.6, 1.8, 1.3 Hz), 7.27 (t, 1H, J = 7.6 Hz), 7.32 (dt, 1H, J = 7.8, 1.5 Hz), 7.39 (t, 1H, J = 1.6 Hz), 7.42–7.48 (m, 4H).

(3′-Methoxy-[1,1′-biphenyl]-3-yl)(4-methoxyphenyl)sulfane (**40**); 67% yield. ^1^H-NMR (CDCl_3_) δ (ppm): 3.83 (s, 3H), 3.84 (s, 3H), 6.88 (ddd, 1H, J = 8.3, 2.6, 0.9 Hz), 6.91 (AA’XX’, 2H, J_AX_ = 8.9 Hz, J_AA’/XX’_ = 2.6 Hz), 7.03 (t, 1H, J = 2.1 Hz), 7.09 (ddd, 1H, J = 7.6, 1.6, 1.0 Hz), 7.13 (ddd, 1H, J = 7.7, 1.8, 1.2 Hz), 7.26–7.33 (m, 2H), 7.35 (dt, 1H, J = 8.2, 1.7 Hz), 7.41 (t, 1H, J = 1.8 Hz), 7.45 (AA’XX’, 2H, J_AX_ = 8.9 Hz, J_AA’/XX’_ = 2.6 Hz).

(3′-Methoxy-[1,1′-biphenyl]-3-yl)(3-methoxyphenyl)sulfane (**41**); 61% yield. ^1^H-NMR (CDCl_3_) δ (ppm): 3.77 (s, 3H), 3.85 (s, 3H), 6.79 (ddd, 1H, J = 8.3, 2.5, 0.9 Hz), 6.88–6.93 (m, 2H), 6.96 (ddd, 1H, J = 7.7, 1.7, 1.0 Hz), 7.07 (t, 1H, J = 2.1 Hz), 7.13 (ddd, 1H, J = 7.6, 1.6, 1.0 Hz), 7.22 (t, 1H, J = 8.0 Hz), 7.31–7.36 (m, 2H), 7.37 (t, 1H, J = 7.6 Hz), 7.47 (dt, 1H, J = 7.4 Hz), 7.61 (t, 1H, J = 1.6 Hz).

#### 3.1.2. General Procedure for the Formation of Compounds **21**, **22**

A vial was loaded with K_3_PO_4_ (1.62 mmol) and intermediate **19** or **20** (1.62 mmol, 324 mg). Then, in an inert atmosphere, copper (I) iodide (0.081 mmol) in anhydrous DMSO (0.6 mL) and commercially available 4-bromoanisole (0.81 mmol) were added. The vial was sealed, and the reaction mixture was stirred at 130 °C. After the reaction mixture was heated for 24 h, it was cooled to room temperature and the workup consisted of the filtration of the reaction mixture through Celite pad, washing it with EtOAc. The filtrate was concentrated under vacuum to give a crude residue, which was then purified by flash chromatography, by using mixtures of n-hexane/EtOAc as eluent, to give intermediates **21** or **22**.

3-Methoxy-3′-(4-methoxyphenoxy)-1,1′-biphenyl (**21**); 28% yield. ^1^H-NMR (CDCl_3_) δ (ppm): 3.81 (s, 3H), 3.85 (s, 3H), 6.87–6.94 (m, 4H), 7.03 (AA’XX’, 2H, J_AX_ = 9.1 Hz, J_AA’/XX’_ = 3.0 Hz), 7.08 (t, 1H, J = 2.1 Hz), 7.13 (ddd, 1H, J = 7.6, 1.6, 0.9 Hz), 7.18 (t, 1H, J = 2.0 Hz), 7.28 (t, 1H, J = 1.3 Hz), 7.30–7.38 (m, 2H).

4′-Methoxy-3-(4-methoxyphenoxy)-1,1′-biphenyl (**22**); 46% yield. ^1^H-NMR (CDCl_3_) δ (ppm): 3.81 (s, 3H), 3.84 (s, 3H), 6.85–6.88 (m, 1H), 6.89 (AA’XX’, 2H, J_AX_ = 9.1 Hz, J_AA’/XX’_ = 2.8 Hz), 6.95 (AA’XX’, 2H, J_AX_ = 8.9 Hz, J_AA’/XX’_ = 2.6 Hz), 7.02 (AA’XX’, 2H, J_AX_ = 9.0 Hz, J_AA’/XX’_ = 3.0 Hz), 7.14 (t, 1H, J = 2.1 Hz), 7.23 (dt, 1H, J = 8.2, 1.3 Hz), 7.33 (t, 1H, J = 7.9 Hz), 7.48 (AA’XX’, 2H, J_AX_ = 8.8 Hz, J_AA’/XX’_ = 2.6 Hz).

#### 3.1.3. General Procedure for the Formation of Compounds **23**, **24**

A sealed vial was charged with Me_4_tBuXPhos (0.0125 mmol), Pd(OAc)_2_ (0.00832 mmol), K_3_PO_4_ (0.832 mmol), intermediate **19** or **20** (0.499 mmol, 100 mg), commercially available 3-bromoanisole (0.416 mmol) and anhydrous toluene (0.8 mL) under a positive pressure of argon. The resulting mixture was heated at 100 °C overnight. Then, the mixture was allowed to cool to room temperature and it was afterwards filtered through a small pad of Celite, washed several times with ethyl acetate, and the filtrate was concentrated under reduced pressure. The crude product was purified by flash chromatography on silica gel (n-hexane/EtOAc mixtures) to afford diarylether derivatives **23** or **24**.

3-Methoxy-3′-(3-methoxyphenoxy)-1,1′-biphenyl (**23**); 58% yield. ^1^H-NMR (CDCl_3_) δ (ppm): 3.79 (s, 3H), 3.85 (s, 3H), 6.60–6.70 (m, 3H), 6.90 (dd, 1H, J = 8.2, 1.8 Hz), 6.97–7.03 (m, 1H), 7.09 (t, 1H, J = 1.9 Hz), 7.12–7.18 (m, 1H), 7.19–7.28 (m, 2H), 7.30–7.43 (m, 3H).

4′-Methoxy-3-(3-methoxyphenoxy)-1,1′-biphenyl (**24**); 71% yield. ^1^H-NMR (CDCl_3_) δ (ppm): 3.79 (s, 3H), 3.85 (s, 3H), 6.60–6.69 (m, 3H), 6.93–6.99 (m, 3H), 7.20–7.27 (m, 2H), 7.30 (ddd, 1H, J = 7.6, 1.6, 1.2 Hz), 7.37 (t, 1H, J = 8.0 Hz), 7.50 (AA’XX’, 2H, J_AX_ = 8.9 Hz, J_AA’/XX’_ = 2.6 Hz).]

Synthesis of tert-butyl (3-bromophenyl)carbamate (**26**). To a solution of commercially available 3-bromoaniline **25** (8.72 mmol, 1.50 g) in anhydrous THF (22.2 mL) were added Et_3_N (2.4 mL) and di-tert-butyl dicarbonate (10.5 mmol) at 0 °C, and the reaction mixture was stirred for 24 h at room temperature. The solvent was removed under reduced pressure, then the residue was diluted with EtOAc and sequentially washed with saturated solution of sodium bicarbonate, water and brine. The organic phase was dried with Na_2_SO_4_ and concentrated. The residue was purified by column chromatography with a mixture of n-hexane/EtOAc 95:5 as eluent to afford intermediate **26** (62% yield). ^1^H-NMR (CDCl_3_) δ (ppm): 1.52 (s, 9H), 6.47 (bs, 1H), 7.10–7.18 (m, 2H), 7.20 (dt, 1H, J = 7.2, 2.0 Hz), 7.67 (s, 1H).

#### 3.1.4. General Procedure for the Formation of Compounds **29**, **30**

Compounds **27** or **28** (2.18 mmol, 653 mg) were dissolved in anhydrous dichloromethane (9.8 mL), cooled to 0 °C, treated with trifluoroacetic acid (3.0 mL), and stirred at room temperature until consumption of starting material (TLC). The mixture was concentrated to dryness under reduced pressure, diluted with EtOAc and washed with 1 M NaHCO_3_ aqueous solution. The organic layer was dried over Na_2_SO_4_ and concentrated to give the title compounds **29** or **30**.

3′-Methoxy-[1,1′-biphenyl]-3-amine (**29**); 85% yield. ^1^H-NMR (CDCl_3_) δ (ppm): 3.86 (s, 3H), 6.70 (ddd, 1H, J = 7.9, 2.3, 0.9 Hz), 6.89 (ddd, 1H, J = 8.2, 2.6, 0.9 Hz), 6.92 (t, 1H, J = 1.9 Hz), 7.00 (ddd, 1H, J = 7.6, 1.6, 1.0 Hz), 7.10 (t, 1H, J = 2.0 Hz), 7.15 (ddd, 1H, J = 7.6, 1.6, 1.0 Hz), 7.23 (t, 1H, J = 7.8 Hz), 7.33 (t, 1H, J = 7.9 Hz).

4′-Methoxy-[1,1′-biphenyl]-3-amine (**30**); 94% yield. ^1^H-NMR (CDCl_3_) δ (ppm): 3.84 (s, 3H), 6.64 (ddd, 1H, J = 7.9, 2.3, 0.9 Hz), 6.87 (t, 1H, J = 1.9 Hz), 6.93–6.98 (m, 3H), 7.20 (t, 1H, J = 7.8 Hz), 7.50 (AA’XX’, 2H, J_AX_ = 8.9 Hz, J_AA’/XX’_ = 2.6 Hz).

#### 3.1.5. General Procedure for the Formation of Compounds **31**–**34**

A solution of Pd_2_dba_3_ (0.0125 mmol), XPhos (0.0502 mmol), K_3_PO_4_ (0.878 mmol), commercially available 3- or 4-bromoanisole (0.627 mmol) and aniline intermediate **29** or **30** (0.753 mmol, 150 mg) in anhydrous toluene (1.3 mL), was stirred at 100 °C under inert atmosphere in a sealed vial for 20 h. The reaction mixture was allowed to cool to room temperature, then filtered through a small pad of Celite, washed with ethyl acetate and concentrated under vacuum. The obtained crude residue was purified by flash column chromatography (eluent mixtures of n-hexane/EtOAc) to give intermediates **31**–**34**.

4′-Methoxy-N-(3-methoxyphenyl)-[1,1′-biphenyl]-3-amine (**31**). Yellow solid; 86% yield. ^1^H-NMR (CDCl_3_) δ (ppm): 3.79 (s, 3H), 3.85 (s, 3H), 6.50 (ddd, 1H, J = 8.2, 2.4, 0.8 Hz), 6.67–6.73 (m, 2H), 6.96 (AA’XX’, 2H, J_AX_ = 8.9 Hz, J_AA’/XX’_ = 2.6 Hz), 7.05 (ddd, 1H, J = 8.0, 2.3, 1.0 Hz), 7.14 (ddd, 1H, J = 7.7, 1.7, 1.0 Hz), 7.18 (t, 1H, J = 8.1 Hz), 7.28 (t, 1H, J = 1.9 Hz), 7.31 (t, 1H, J = 7.9 Hz), 7.50 (AA’XX’, 2H, J_AX_ = 8.9 Hz, J_AA’/XX’_ = 2.6 Hz). ^13^C-NMR (CDCl_3_) δ (ppm): 55.37, 55.49, 103.66, 106.46, 110.47, 114.30 (2C), 116.74, 116.81, 119.98, 128.26 (2C), 129.82, 130.28, 133.78, 142.28, 143.37, 144.71, 159.37, 160.89. HPLC analysis: retention time = 13.870; peak area 95% (254 nm). HRMS: *m*/*z* for C_20_H_20_NO_2_ [M + H]^+^ calculated: 306.14886, found: 306.14880.

4′-Methoxy-N-(4-methoxyphenyl)-[1,1′-biphenyl]-3-amine (**32**). Light-yellow solid; 91% yield. ^1^H-NMR (acetone-d_6_) δ (ppm): 3.77 (s, 3H), 3.82 (s, 3H), 6.85–7.03 (m, 6H), 7.08–7.27 (m, 5H), 7.48–7.56 (m, 2H). ^13^C-NMR (CDCl_3_) δ (ppm): 55.46, 55.71, 114.15, 114.20, 114.22 (4C), 114.85, 118.41, 122.51, 128.24 (2C), 129.77, 134.01, 135.77, 142.20, 145.66, 155.51, 159.28. HPLC analysis: retention time = 13.768; peak area 96% (254 nm). HRMS: *m*/*z* for C_20_H_20_NO_2_ [M + H]^+^ calculated: 306.14886, found: 306.14874.

3′-Methoxy-N-(4-methoxyphenyl)-[1,1′-biphenyl]-3-amine (**33**); 39% yield. ^1^H-NMR (acetone-d_6_) δ (ppm): 3.77 (s, 3H), 3.85 (s, 3H), 6.87–6.93 (m, 3H), 6.96 (ddd, 1H, J = 8.1, 2.4, 0.9 Hz), 7.02 (ddd, 1H, J = 7.6, 1.7, 1.0 Hz), 7.10–7.13 (m, 1H), 7.13–7.18 (m, 3H), 7.19 (bs, 1H), 7.21–7.24 (m, 1H), 7.25 (t, 1H, J = 7.9 Hz), 7.34 (t, 1H, J = 7.9 Hz).

3′-Methoxy-N-(3-methoxyphenyl)-[1,1′-biphenyl]-3-amine (**34**); 54% yield. ^1^H-NMR (CDCl_3_) δ (ppm): 3.79 (s, 3H), 3.86 (s, 3H), 6.51 (ddd, 1H, J = 8.2, 2.4, 0.8 Hz), 6.67–6.74 (m, 2H), 6.90 (ddd, 1H, J = 8.2, 2.6, 0.9 Hz), 7.07–7.13 (m, 2H), 7.13–7.21 (m, 3H), 7.29–7.37 (m, 3H).

#### 3.1.6. General Procedure for the Formation of Compounds **36**, **37**

Cu(I) iodide (0.0795 mmol), potassium carbonate (3.18 mmol), commercially available 3- or 4-iodoanisoloe (1.59 mmol) and 3-bromothiophenol **35** (1.59 mmol, 300 mg) were dissolved in a mixture of isopropanol (1.1 mL) and ethylene glycol (0.2 mL) in a screw-capped test tube under inert atmosphere. The reaction was heated to 80 °C and stirred for 18 h. After cooling to room temperature, the mixture was diluted with water and extracted several times with EtOAc. The combined organic phases were washed with brine, dried over anhydrous sodium sulphate and concentrated under vacuum. The crude product was purified by flash column chromatography on silica gel, using petroleum ether as the eluent to afford the desired thioether derivatives **36** or **37**.

(3-Bromophenyl)(3-methoxyphenyl)sulfane (**36**); 83% yield. ^1^H-NMR (CDCl_3_) δ (ppm): 3.78 (s, 3H), 6.84 (ddd, 1H, J = 8.3, 2.5, 0.8 Hz), 6.92 (t, 1H, J = 2.1 Hz), 6.97 (ddd, 1H, J = 7.7, 1.5, 0.9 Hz), 7.15 (t, 1H, J = 7.9 Hz), 7.20–7.28 (m, 2H), 7.34 (ddd, 1H, J = 7.9, 1.8, 1.1 Hz), 7.44 (t, 1H, J = 1.8 Hz).

(3-Bromophenyl)(4-methoxyphenyl)sulfane (**37**); 46% yield. ^1^H-NMR (CDCl_3_) δ (ppm): 3.84 (s, 3H), 6.93 (AA’XX’, 2H, J_AX_ = 8.9 Hz, J_AA’/XX’_ = 2.6 Hz), 7.02–7.11 (m, 2H), 7.21–7.26 (m, 2H), 7.43 (AA’XX’, 2H, J_AX_ = 8.9 Hz, J_AA’/XX’_ = 2.6 Hz).

#### 3.1.7. General Procedure for the Synthesis of O-Deprotected Compounds **6**–**17**

A solution of methoxylated intermediate **21**–**24**, **31**–**34** or **38**–**41** (0.349 mmol, 107 mg) in anhydrous dichloromethane (4.1 mL) was cooled to -15 °C and treated dropwise with a 1 M solution of BBr_3_ in dichloromethane (2.2 mL), and the resulting solution was stirred at 0 °C for 1 h and at room temperature until the reaction was complete (disappearance of starting material by TLC analysis). The mixture was then diluted with water and extracted with ethyl acetate. The organic phase was washed with brine, dried and concentrated. The crude product was purified by chromatography over silica gel (n-hexane/ethyl acetate mixtures) to yield pure phenolic compounds **6**–**17**.

3′-(3-Hydroxyphenoxy)-[1,1′-biphenyl]-4-ol (**6**). Light-pink solid; 76% yield. ^1^H-NMR (acetone-d_6_) δ (ppm): 6.50–6.54 (m, 2H), 6.62 (ddd, 1H, J = 8.1, 2.3, 0.9 Hz), 6.90–6.95 (m, 3H), 7.16–7.22 (m, 1H), 7.23 (t, 1H, J = 1.9 Hz), 7.37 (dt, 1H, J = 8.2, 1.5 Hz), 7.42 (t, 1H, J = 7.8 Hz), 7.50 (AA’XX’, 2H, J_AX_ = 8.9 Hz, J_AA’/XX’_ = 2.6 Hz), 8.49 (s, 1H). ^13^C-NMR (acetone-d_6_) δ (ppm): 106.64, 110.45, 111.26, 116.58 (2C), 117.67, 117.78, 122.18, 128.84 (2C), 130.98, 131.18, 132.32, 143.74, 158.31, 158.45, 159.51, 159.74. HPLC analysis: retention time = 11.817; peak area 99% (254 nm). HRMS: *m*/*z* for C_18_H_13_O_3_ [M − H]^−^ calculated: 277.08702, found: 277.08704.

3′-(4-Hydroxyphenoxy)-[1,1′-biphenyl]-4-ol (**7**). White solid; 77% yield. ^1^H-NMR (acetone-d_6_) δ (ppm): 6.82 (ddd, 1H, J = 8.1, 2.5, 1.0 Hz), 6.88 (AA’XX’, 2H, J_AX_ = 9.1 Hz, J_AA’/XX’_ = 2.9 Hz), 6.91 (AA’XX’, 2H, J_AX_ = 8.8 Hz, J_AA’/XX’_ = 2.6 Hz), 6.95 (AA’XX’, 2H, J_AX_ = 9.1 Hz, J_AA’/XX’_ = 2.9 Hz), 7.12 (t, 1H, J = 2.0 Hz), 7.26 (ddd, 1H, J = 7.7, 1.7, 1.1 Hz), 7.35 (t, 1H, J = 7.9 Hz), 7.46 (AA’XX’, 2H, J_AX_ = 8.8 Hz, J_AA’/XX’_ = 2.6 Hz), 8.28 (s, 1H), 8.47 (s, 1H). ^13^C-NMR (acetone-d_6_) δ (ppm): 115.90, 115.99, 116.57, 117.13, 121.91 (4C), 128.83 (2C), 130.83 (2C), 132.60, 143.60, 149.92, 154.79, 158.25, 160.32. HPLC analysis: retention time = 11.640; peak area 98% (254 nm). HRMS: *m*/*z* for C_18_H_13_O_3_ [M − H]^−^ calculated: 277.08702, found: 277.08710.

3′-(4-Hydroxyphenoxy)-[1,1′-biphenyl]-3-ol (**8**). White solid; 95% yield. ^1^H-NMR (acetone-d_6_) δ (ppm): 6.83 (ddd, 1H, J = 8.1, 2.5, 0.9 Hz), 6.86–6.91 (m, 3H), 6.97 (AA’XX’, 2H, J_AX_ = 9.0 Hz, J_AA’/XX’_ = 2.9 Hz), 7.03–7.08 (m, 2H), 7.13 (t, 1H, J = 2.0 Hz), 7.23–7.31 (m, 2H), 7.39 (t, 1H, J = 7.9 Hz), 8.30 (s, 1H), 8.43 (s, 1H). ^13^C-NMR (acetone-d_6_) δ (ppm): 114.56, 115.49, 116.28, 116.89, 117.17 (2C), 118.97 (2C), 121.60, 122.03, 130.80, 130.93, 142.83, 143.66, 149.77, 154.89, 158.75, 160.36. HPLC analysis: retention time = 11.739; peak area 100% (254 nm). HRMS: *m*/*z* for C_18_H_13_O_3_ [M − H]^−^ calculated: 277.08702, found: 277.08682.

3′-(3-Hydroxyphenoxy)-[1,1′-biphenyl]-3-ol (**9**). Light-green oil; 71% yield. ^1^H-NMR (acetone-d_6_) δ (ppm): 6.51–6.55 (m, 2H), 6.63 (ddd, 1H, J = 8.1, 2.3, 0.9 Hz), 6.85 (ddd, 1H, J = 8.1, 2.4, 1.0 Hz), 7.01 (ddd, 1H, J = 8.0, 2.4, 1.1 Hz), 7.07–7.12 (m, 2H), 7.20 (t, 1H, J = 8.4 Hz), 7.24 (t, 1H, J = 2.0 Hz), 7.28 (t, 1H, J = 7.8 Hz), 7.39 (dt, 1H, J = 8.2, 1.4 Hz), 7.46 (t, 1H, J = 7.9 Hz), 8.42–8.56 (bm, 2H). ^13^C-NMR (acetone-d_6_) δ (ppm): 106.81, 110.61, 111.43, 114.58, 115.57, 118.10, 118.67, 118.98, 122.71, 130.84, 131.10, 131.26, 142.60, 143.82, 158.54, 158.78, 159.39, 159.79. HPLC analysis: retention time = 11.940; peak area 96% (254 nm). HRMS: *m*/*z* for C_18_H_13_O_3_ [M − H]^−^ calculated: 277.08702, found: 277.08710.

3′-((3-Hydroxyphenyl)amino)-[1,1′-biphenyl]-4-ol (**10**). Yellow solid; 72% yield. ^1^H-NMR (acetone-d_6_) δ (ppm): 6.36 (ddd, 1H, J = 8.0, 2.3, 0.8 Hz), 6.61–6.66 (m, 1H), 6.71 (t, 1H, J = 2.2 Hz), 6.91 (AA’XX’, 2H, J_AX_ = 8.7 Hz, J_AA’/XX’_ = 2.5 Hz), 7.03–7.09 (m, 3H), 7.28 (t, 1H, J = 7.8 Hz), 7.34 (t, 1H, J = 1.9 Hz), 7.37 (bs, 1H), 7.47 (AA’XX’, 2H, J_AX_ = 8.6 Hz, J_AA’/XX’_ = 2.5 Hz), 8.15 (s, 1H), 8.42 (s, 1H). ^13^C-NMR (acetone-d_6_) δ (ppm): 104.83, 108.26, 109.64, 116.40, 116.49 (2C), 116.60, 119.22, 128.75 (2C), 130.34, 130.79, 133.44, 142.86, 145.04, 146.04, 158.02, 159.28. HPLC analysis: retention time = 11.306; peak area 97% (254 nm). HRMS: *m*/*z* for C_18_H_14_NO_2_ [M − H]^−^ calculated: 276.10300, found: 276.10300.

3′-((4-Hydroxyphenyl)amino)-[1,1′-biphenyl]-4-ol (**11**). Light-pink solid; 83% yield. ^1^H-NMR (acetone-d_6_) δ (ppm): 6.81 (AA’XX’, 2H, J_AX_ = 8.8 Hz, J_AA’/XX’_ = 2.8 Hz), 6.85 (ddd, 1H, J = 8.1, 2.3, 0.9 Hz), 6.89 (AA’XX’, 2H, J_AX_ = 8.7 Hz, J_AA’/XX’_ = 2.5 Hz), 6.93 (ddd, 1H, J = 7.6, 1.7, 1.0 Hz), 7.02 (bs, 1H), 7.07 (AA’XX’, 2H, J_AX_ = 8.8 Hz, J_AA’/XX’_ = 2.8 Hz), 7.13 (t, 1H, J = 2.0 Hz), 7.19 (t, 1H, J = 7.8 Hz), 7.42 (AA’XX’, 2H, J_AX_ = 8.7 Hz, J_AA’/XX’_ = 2.5 Hz), 8.38 (s, 1H), 8.01 (s, 1H). ^13^C-NMR (acetone-d_6_) δ (ppm): 113.69, 113.91, 116.43 (2C), 116.71 (2C), 117.50, 123.17 (2C), 128.72 (2C), 130.30, 133.77, 136.04, 142.83, 147.57, 153.58, 157.91. HPLC analysis: retention time = 10.992; peak area 99% (254 nm). HRMS: *m*/*z* for C_18_H_14_NO_2_ [M − H]^−^ calculated: 276.10300, found: 276.10297.

3′-((4-Hydroxyphenyl)amino)-[1,1′-biphenyl]-3-ol (**12**). Yellow oil; 98% yield. ^1^H-NMR (acetone-d_6_) δ (ppm): 6.77–6.85 (m, 3H), 6.87–6.93 (m, 1H), 6.93–6.98 (m, 1H), 7.01–7.10 (m, 5H), 7.16 (t, 1H, J = 1.9 Hz), 7.18–7.26 (m, 2H), 8.04 (s, 1H), 8.36 (s, 1H). ^13^C-NMR (acetone-d_6_) δ (ppm): 113.95, 114.53, 114.66, 115.01, 116.71 (2C), 117.83, 118.92, 123.33, 130.33 (2C), 130.58, 135.86, 142.88, 143.97, 147.64, 153.67, 158.62. HPLC analysis: retention time = 11.232; peak area 99% (254 nm). HRMS: *m*/*z* for C_18_H_14_NO_2_ [M − H]^−^ calculated: 276.10300, found: 276.10294.

3′-((3-Hydroxyphenyl)amino)-[1,1′-biphenyl]-3-ol (**13**). Yellow solid; 86% yield. ^1^H-NMR (acetone-d_6_) δ (ppm): 6.37 (ddd, 1H, J = 8.1, 2.3, 0.9 Hz), 6.64 (ddd, 1H, J = 8.1, 2.1, 0.8 Hz), 6.72 (t, 1H, J = 2.2 Hz), 6.82 (ddd, 1H, J = 8.1, 2.4, 1.0 Hz), 7.04–7.12 (m, 5H), 7.26 (t, 1H, J = 8.1 Hz), 7.31 (t, 1H, J = 7.9 Hz), 7.37 (t, 1H, J = 1.8 Hz), 7.42 (bs, 1H), 8.18 (s, 1H), 8.40 (s, 1H). ^13^C-NMR (acetone-d_6_) δ (ppm): 104.97, 108.43, 109.79, 114.61, 115.20, 116.70, 117.38, 118.98, 119.62, 130.44, 130.70, 130.87, 142.96, 143.70, 145.15, 145.91, 158.73, 159.31. HPLC analysis: retention time = 11.445; peak area 98% (254 nm). HRMS: *m*/*z* for C_18_H_14_NO_2_ [M − H]^−^ calculated: 276.10300, found: 276.10297.

3′-((3-Hydroxyphenyl)thio)-[1,1′-biphenyl]-4-ol (**14**). White solid; 99% yield. ^1^H-NMR (acetone-d_6_) δ (ppm): 6.76 (ddd, 1H, J = 8.1, 2.4, 0.9 Hz), 6.83 (t, 1H, J = 1.8 Hz), 6.86 (ddd, 1H, J = 7.7, 1.7, 0.9 Hz), 6.92 (AA’XX’, 2H, J_AX_ = 8.8 Hz, J_AA’/XX’_ = 2.6 Hz), 7.19 (t, 1H, J = 7.8 Hz), 7.28 (ddd, 1H, J = 7.7, 1.8, 1.1 Hz), 7.42 (td, 1H, J = 7.7, 0.4 Hz), 7.48 (AA’XX’, 2H, J_AX_ = 8.8 Hz, J_AA’/XX’_ = 2.6 Hz), 7.53 (ddd, 1H, J = 7.8, 1.8, 1.1 Hz), 7.59 (td, 1H, J = 1.8, 0.4 Hz), 8.49 (bs, 2H). ^13^C-NMR (acetone-d_6_) δ (ppm): 115.28, 116.71 (2C), 118.11, 122.54, 126.27, 128.91 (2C), 129.91, 130.12, 130.62, 131.11, 132.21, 136.73, 137.72, 143.07, 158.43, 159.05. HPLC analysis: retention time = 12.253; peak area 95% (254 nm). HRMS: *m*/*z* for C_18_H_13_O_2_S [M − H]^−^ calculated: 293.06417, found: 293.06427.

3′-((4-Hydroxyphenyl)thio)-[1,1′-biphenyl]-4-ol (**15**). Colorless oil; 78% yield. ^1^H-NMR (acetone-d_6_) δ (ppm): 6.90 (AA’XX’, 2H, J_AX_ = 8.8 Hz, J_AA’/XX’_ = 2.6 Hz), 6.93 (AA’XX’, 2H, J_AX_ = 8.8 Hz, J_AA’/XX’_ = 2.6 Hz), 7.04 (ddd, 1H, J = 7.7, 1.8, 1.3 Hz), 7.30 (td, 1H, J = 7.6, 0.7 Hz), 7.34–7.38 (m, 2H), 7.38–7.44 (m, 4H), 8.48 (bs, 1H), 8.69 (bs, 1H). ^13^C-NMR (acetone-d_6_) δ (ppm): 116.61, 117.56 (2C), 123.02, 124.63, 126.23, 126.47, 128.83 (4C), 130.23, 132.51, 136.88, 140.55, 142.69, 158.29, 159.15. HPLC analysis: retention time = 12.167; peak area 95% (254 nm). HRMS: *m*/*z* for C_18_H_13_O_2_S [M − H]^−^ calculated: 293.06417, found: 293.06436.

3′-((4-Hydroxyphenyl)thio)-[1,1′-biphenyl]-3-ol (**16**). Colorless oil; 92% yield. ^1^H-NMR (acetone-d_6_) δ (ppm): 6.83 (ddd, 1H, J = 8.1, 2.4, 1.0 Hz), 6.93 (AA’XX’, 2H, J_AX_ = 8.8 Hz, J_AA’/XX’_ = 2.6 Hz), 7.00–7.04 (m, 2H), 7.11 (ddd, 1H, J = 7.6, 1.9, 1.3 Hz), 7.25 (t, 1H, J = 8.1 Hz), 7.31–7.40 (m, 3H), 7.42 (AA’XX’, 2H, J_AX_ = 8.8 Hz, J_AA’/XX’_ = 2.6 Hz), 8.58 (bs, 2H). ^13^C-NMR (acetone-d_6_) δ (ppm): 114.61, 115.52, 117.62 (2C), 118.96, 122.77, 125.14, 126.62, 127.24, 130.28, 130.81, 137.01 (2C), 140.76, 142.78, 142.82, 158.78, 159.25. HPLC analysis: retention time = 12.263; peak area 95% (254 nm). HRMS: *m*/*z* for C_18_H_13_O_2_S [M − H]^−^ calculated: 293.06417, found: 293.06418.

3′-((3-Hydroxyphenyl)thio)-[1,1′-biphenyl]-3-ol (**17**). Light-green oil; 99% yield. ^1^H-NMR (acetone-d_6_) δ (ppm): 6.77 (ddd, 1H, J = 8.1, 2.4, 0.9 Hz), 6.82–6.90 (m, 3H), 7.05–7.11 (m, 2H), 7.20 (t, 1H, J = 7.8 Hz), 7.27 (t, 1H, J = 8.1 Hz), 7.34 (ddd, 1H, J = 7.7, 1.7, 1.1 Hz), 7.44 (t, 1H, J = 7.7 Hz), 7.55 (ddd, 1H, J = 7.7, 1.7, 1.1 Hz), 7.59 (t, 1H, J = 1.6 Hz), 8.49 (bs, 2H). ^13^C-NMR (acetone-d_6_) δ (ppm): 114.64, 115.40, 115.65, 118.26, 119.00, 122.70, 126.76, 130.27, 130.66, 130.86, 130.89, 131.16, 136.97, 137.45, 142.45, 143.13, 158.82, 159.05. HPLC analysis: retention time = 12.295; peak area 96% (254 nm). HRMS: *m*/*z* for C_18_H_13_O_2_S [M − H]^−^ calculated: 293.06417, found: 293.06415.

### 3.2. Docking Calculations

The compounds were built with Maestro and then subjected to energy minimization performed with Macromodel, until a convergence value of 0.05 kcal/Å·mol, by employing the conjugate gradient (CG) algorithm, MMFFs force field and a distance-dependent dielectric constant of 1.0. The compounds were docked into the X-ray structure of SIRT1 in complex with the p53-AMC peptide and resveratrol (PDB code 5BTR [20]). All docking calculations were performed with GOLD software, using ChemScore fitness function. For each docked compound, we performed three different docking studies related to the three different receptor subsites occupied by the three resveratrol molecules in the reference X-ray complex. In each calculation, the docking site included all residues which stayed within a 10 Å shell from the specific bound ligand in the reference X-ray complex. The compounds were subjected to 100 genetic algorithm runs, in which the “allow early termination” option was deactivated, while the possibility for the ligand to flip ring corners was activated. All other settings were left as their defaults. The root-mean-squared deviation (RMSD) threshold for pose clustering was set to 2.0 Å. The best docked conformation belonging to the best cluster of solutions (top-scored pose) was considered for each ligand in each docking study. By following this procedure, resveratrol was properly self-docked in each of the three receptor sub-sites, with RMSD values between each top-scored pose and the corresponding experimental disposition below 2.0 Å, thus validating the reliability of the protocol. Two out of the three top-scored poses obtained for each of the phenolic derivatives **10** and **13** converged in a single binding mode. For this reason, the final corresponding SIRT1–peptide–ligand complexes predicted by docking included only two ligand molecules.

### 3.3. Molecular Dynamics Simulations

MD simulations were carried out using AMBER, version 20, using the ff14SB force field. General Amber force field (GAFF) parameters were used for the ligand, whose partial charges were assigned using the Antechamber suite of AMBER 20, based on the AM1-BCC method. The reference SIRT1–peptide–resveratrol complex (PDB code 5BTR) and the two predicted SIRT1–peptide–ligand complexes were placed at the center of a rectangular parallelepiped box and solvated with a 15 Å water cap, generated using TIP3P explicit solvent model. Sodium ions were added to neutralize the solvated systems, which were then energy-minimized using a two-step protocol. In the first step, only the minimization of the solvent was performed by applying a harmonic restraint of 100 kcal/mol·Å^2^ on all solute atoms. In the second step, 5000 cycles of steepest descent followed by CG were used to minimize the whole system, until a convergence of 0.05 kcal/Å·mol. The minimized complexes were used as input structures for the MD simulations, which were run using Particle Mesh Ewald (PME) electrostatics, a cutoff of 10 Å for the nonbonded interactions and periodic boundary conditions. The SHAKE algorithm was used to constrain all bonds involving hydrogen atoms and a time step of 2.0 fs was thus used for the simulations, following a protocol optimized from previous studies [21]. Initially, a heating stage of 1 ns, in which the temperature of the system was raised from 0 to 300 K, was performed using constant-volume periodic boundary conditions. An initial equilibration stage of constant-pressure periodic boundary MD was run for 3 ns, keeping the temperature of the system at the constant value of 300 K with a Langevin thermostat. A second longer constant-pressure MD (20 ns) was then performed at 300 K for assuring the equilibration of the protein-peptide-ligand binding conformation. Finally, a 500 ns production step was performed, maintaining the same constant pressure and temperature conditions. All α carbons of the protein were subjected to a harmonic potential of 10 kcal/mol·Å^2^ during all MD stages, for a total of 524 ns of simulation. The final structures of the different complexes corresponded to the average of the last 250 ns of MD simulation minimized by the CG method until a convergence of 0.05 kcal/mol·Å^2^. The average structures were obtained using the Cpptraj program implemented in AMBER 20.

### 3.4. In Vitro Screening on Isolated SIRT1 Enzyme

The effects of the new compounds on SIRT1 activity were evaluated by a direct enzymatic assay using a SIRT1 Direct Fluorescent Screening Assay Kit (Cayman Chemical, Ann Arbor, MI, USA), following the protocol user guide. Fluorescence was analyzed with the EnSpire spectrofluorometer (PerkinElmer, Waltham, MA, USA) at a wavelength of 350–360 nm in excitation and 450–465 nm in emission. The increase in recorded fluorescence was directly proportional to the activation of SIRT1. Data obtained were analyzed by removing baseline and normalizing to the fluorescence value of the vehicle (100%), dimethylsulfoxide (DMSO) 0.1%; the SIRT1 activator resveratrol (Sigma-Aldrich, St.Louis, MO, USA) was used as positive control at the concentration of 100 µM. One-way ANOVA followed by Bonferroni’s post hoc test was selected as statistical analysis, and the difference between groups was considered statistically different when *p* < 0.05.

### 3.5. Ex Vivo Evaluation on Myocardial Ischemia/Reperfusion Model

All procedures were performed according to European (EEC Directive 2010/63) and Italian (D.L. 4 March 2014 n. 26) legislation (protocol number 45972, 21 September 2016). Animals were housed in cages with food and water ad libitum and exposed to 12 h:12 h light/dark cycles. Adult male Wistar rats, 350–400 g, were treated with an intraperitoneal injection of compound **10** (at the dose 50 mg/kg or 100 mg/kg) or vehicle (dimethyl sulfoxide; DMSO, 0.35–0.4 mL). After 2 h, the rats were anaesthetized with sodium pentobarbital (100 mg/kg, i.p.) and heparinized (100 U, i.p.) to prevent blood clotting. Subsequently, the hearts were quickly excised, mounted on a Langendorff apparatus and perfused at constant pressure (70–80 mmHg), as previously published [22,23]. To check the functional parameters of the heart, a latex balloon filled with bidistilled water at a pressure of 5–10 mmHg was inserted into the left ventricle through the mitral valve and connected to a pressure transducer (Bentley Trantec, mod 800, Ugo Basile, Comerio, Italy) and, in turn, to a data acquisition system (Biopac Systems, Goleta, CA, USA). The functional parameter of rate pressure product (RPP) was calculated as RPP = HR x LVDP, where HR was the heart rate value and LVDP was the left ventricular developed pressure, continuously monitored by a computerized Biopac Systems (Goleta, CA, USA). After 30 min of equilibration, the hearts were submitted to 30 min of global ischemia. Then, hearts were reperfused for 120 min. At the end of reperfusion, the RPP value was acquired, and the hearts were removed from the apparatus, dried and subsequently the left ventricle was isolated. The latter was cut in slices of about 2 mm, which were immersed in a 1% *w*/*v* solution of 2,3,5 triphenyltetrazolium chloride (TTC, Sigma-Aldrich, Darmstadt, Germany) dissolved in a phosphate buffer (pH = 7.4) for 20 min in the dark and at 37 °C. TTC reacts with dehydrogenases present in intact and viable cells, and oxidizes to formazan, a red and insoluble compound. Then, slices were fixed in a water solution of formaldehyde 10% *v*/*v*. Subsequently, the ventricular slices were photographed and analyzed to identify the necrotic areas (visible as a white or light-pink color) and the healthy areas (visible as a strong red due to the TTC reaction). Ischemic area was excessed as a percentage of the total area of the left ventricle (A_I_/A_LV_%). All values are expressed as a mean ± standard error for six different experiments. The effects of compound **10** on RPP and A_I_/A_LV_ were statistically analyzed by Student’s *t*-test. *p* < 0.05 was considered indicative of a significant difference.

## 4. Conclusions

Sirtuins belong to class III histone deacetylases and their function is the NAD^+^-mediated removal of acyl groups from protein lysine residues. Among the seven human sirtuins (SIRT1-7), a growing body of evidence suggests that SIRT1 modulates many signaling pathways by deacetylating substrate proteins involved in metabolic diseases, neurodegeneration, cancer and inflammation, thus linking SIRT1 to several diseases [24]. Many activators and inhibitors have been discovered to modulate SIRT1 activity [25] and, in this context, we identified a new chemical class of SIRT1 activators. The design of the newly synthesized derivatives reported in this work was inspired by the common general polyphenolic structure of natural SIRT1 activators such as resveratrol, quercetin and naringenin. In fact, the new compounds shared common features, such as antipodal phenolic rings, which are connected by a 1,3-disubstituted phenyl ring and an oxygen atom, a sulfur atom or a NH group. Diarylether derivatives **6**–**9**, diarylamine derivatives **10**–**13** and diarylsulphide derivatives **14**–**17** were synthesized and subjected to enzymatic assays to evaluate their SIRT1 activating ability with respect to the well-known SIRT1 activator resveratrol. The best result was obtained by bisarylaniline compound **10**, which reached a 136% activation, compared to that of the vehicle, which was set at 100% and with the reference compound resveratrol (174%). A SAR analysis highlighted that the NH spacer and the para-hydroxy-substituted diphenyl moiety of compound **10** are necessary to reach an efficient SIRT1 activation. Compound **10** was investigated in an ex vivo I/R protocol, where it proved to reduce myocardial damage induced by ischemic events, thus supporting a cardioprotective profile of this derivative. Molecular modeling studies suggested a potential binding orientation for **10** inside SIRT1 in the presence of p53-AMC peptide.

Taken together, these results identified compound **10** as an efficient SIRT1 activator endowed of a promising cardioprotective effect. Further studies will be necessary to better optimize the chemical scaffold as well as to enlarge the class of phenolic diarylamines. Moreover, evaluation of the toxicity and exploration of the activity on other epigenetic targets will be addressed for the most active compounds in in vitro and in vivo models, with the aim of finding more potent and selective SIRT1 activators.

## Figures and Tables

**Table 1:** The table shows the % of activation of the tested compounds in comparison to resveratrol. n.a. = no activation.

Compound	% SIRT1 Activation
**Resveratrol**	174 ± 5
**6**	n.a.
**7**	n.a.
**8**	n.a.
**9**	n.a.
**10**	136 ± 7
**11**	133 ± 3
**12**	n.a.
**13**	n.a.
**14**	n.a.
**15**	n.a.
**16**	n.a.
**17**	n.a.
**31**	n.a.
**32**	n.a.

## Data Availability

Data is contained within the article and Supplementary Materials.

## Supplementary Materials

The following supporting information can be downloaded at: https://www.mdpi.com/article/10.3390/ph15030339/s1, Figures S1–S14: RP-HPLC traces of the final compounds; Figures S15–S28: NMR spectra of the final compounds; Figure S29: RMSD analysis of the MD of the reference SIRT1 X-ray complex; Figures S30–S43: ESI-HRMS spectra of the final compounds.
