# Supplementary material for: New Synthetic Analogues of Natural Polyphenols as Sirtuin 1-Activating Compounds
Source: Pharmaceuticals (Basel). 2022 Mar 10;15(3):339. doi: 10.3390/ph15030339 (PMC8949162; doi:10.3390/ph15030339)
Supplement: Supplementary file 1 [file pharmaceuticals-15-00339-s001.zip › pharmaceuticals-1599827-supplementary.pdf]

Supporting Information

New synthetic analogues of natural polyphenols as sirtuin 1-activating compounds

Giulia Bononi, Lorenzo Flori, Valentina Citi, Cecilia Acciai, Viviana Nocilla, Alma Martelli, Giulio Poli, Tiziano Tuccinardi, Carlotta Granchi, Lara Testai, Vincenzo Calderone, Filippo Minutolo.

Table of Contents

Figure S1-S14. RP-HPLC traces of the final compounds. S2-S15

Figure S15-S28. NMR spectra of the final compounds. S16-S29

Figure S29. RMSD analysis of the MD of the reference SIRT1 X-ray complex S30

Figure S30-S43. ESI-HRMS spectra of the final compounds. S31-S37

==== Shimadzu LabSolutions Analysis Report ====

Sample Name : SIRT6_500uM
Sample ID : SIRT6_500uM
Data Filename : SIRT6_500uM_p.lcd
Method Filename : MAGL254.lcm

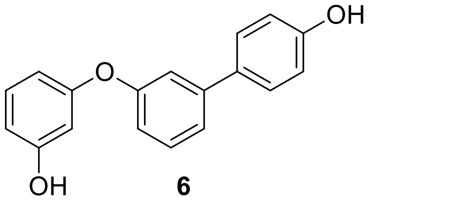

6

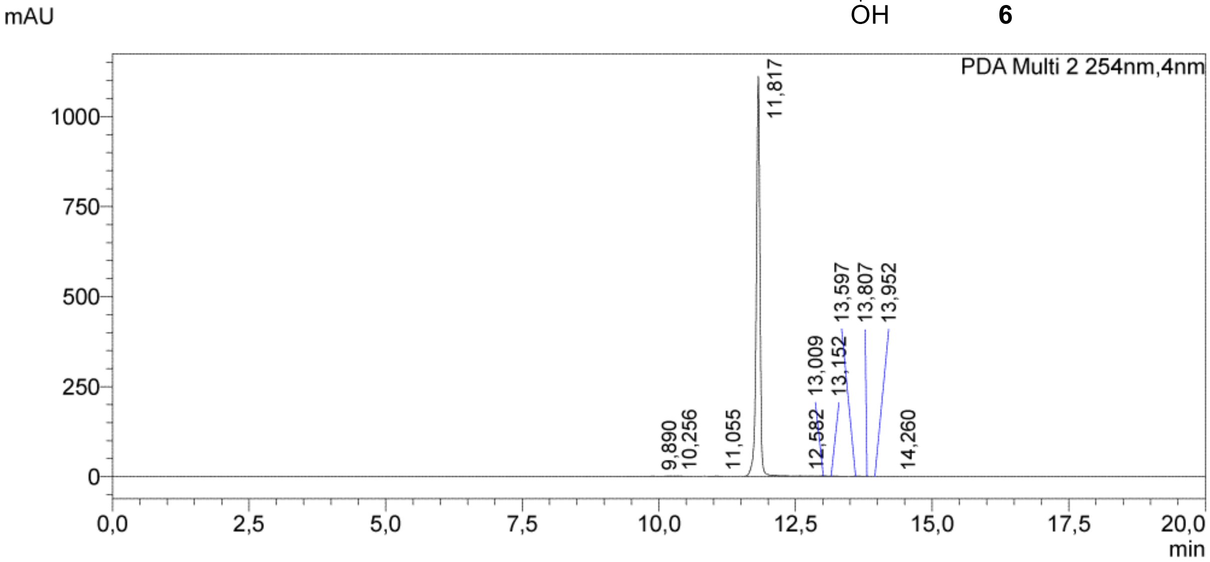

PDA Ch2 254nm

Peak#	Ret. Time	Area	Area%	Height
1	9,890	5427	0,105	340
2	10,256	9439	0,182	424
3	11,055	1082	0,021	154
4	11,817	5134027	99,168	1111240
5	12,582	3193	0,062	730
6	13,009	11015	0,213	1414
7	13,152	5582	0,108	723
8	13,597	3971	0,077	597
9	13,807	1263	0,024	255
10	13,952	1058	0,020	133
11	14,260	1061	0,020	250
Total		5177118	100,000	1116260

Figure S1. HPLC chromatogram of compound **6**.

==== Shimadzu LabSolutions Analysis Report ====

Sample Name : SIRT7_500uM
Sample ID : SIRT7_500uM
Data Filename : SIRT7_500uM_p.lcd
Method Filename : MAGL254.lcm

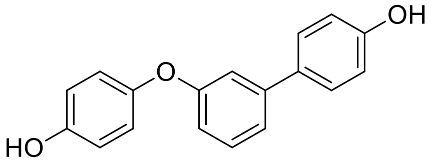

7

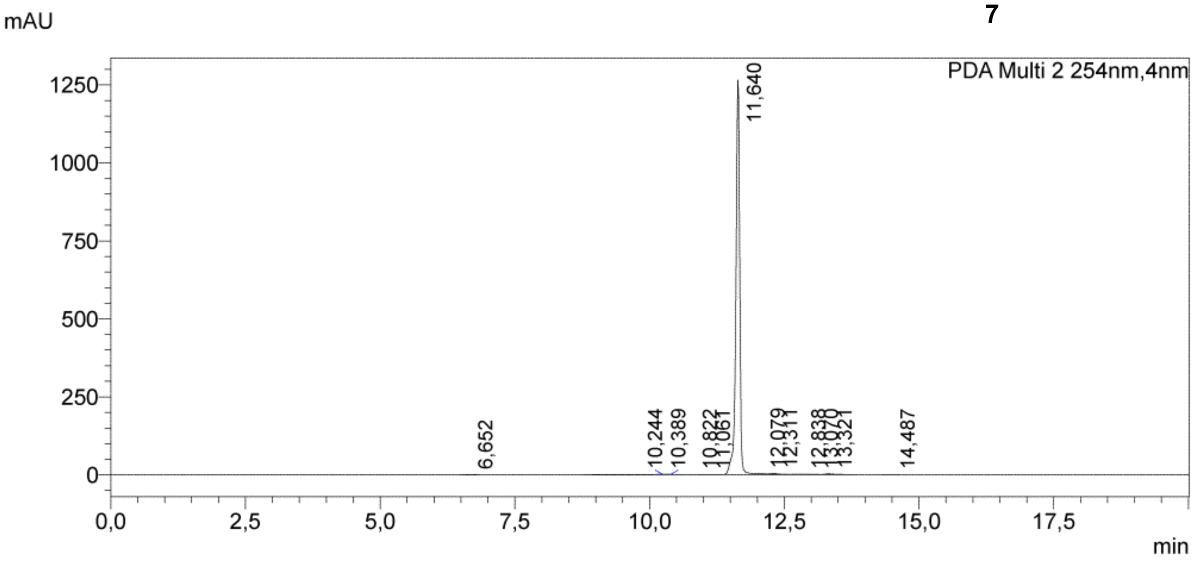

PDA Ch2 254nm

Peak#	Ret. Time	Area	Area%	Height
1	6,652	3138	0,049	276
2	10,244	45494	0,714	1132
3	10,389	10649	0,167	974
4	10,822	10047	0,158	661
5	11,061	14041	0,220	549
6	11,640	6234628	97,783	1264908
7	12,079	3629	0,057	740
8	12,311	16962	0,266	2541
9	12,838	5032	0,079	619
10	13,070	3067	0,048	443
11	13,321	27491	0,431	2414
12	14,487	1816	0,028	180
Total		6375994	100,000	1275439

Figure S2. HPLC chromatogram of compound **7**.

==== Shimadzu LabSolutions Analysis Report ====

Sample Name : SIRT9_500uM
Sample ID : SIRT9_500uM
Data Filename : SIRT9_500uM_p.lcd
Method Filename : MAGL254.lcm

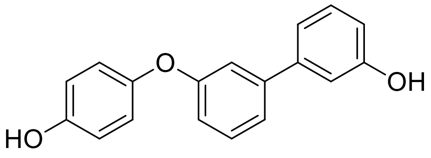

8

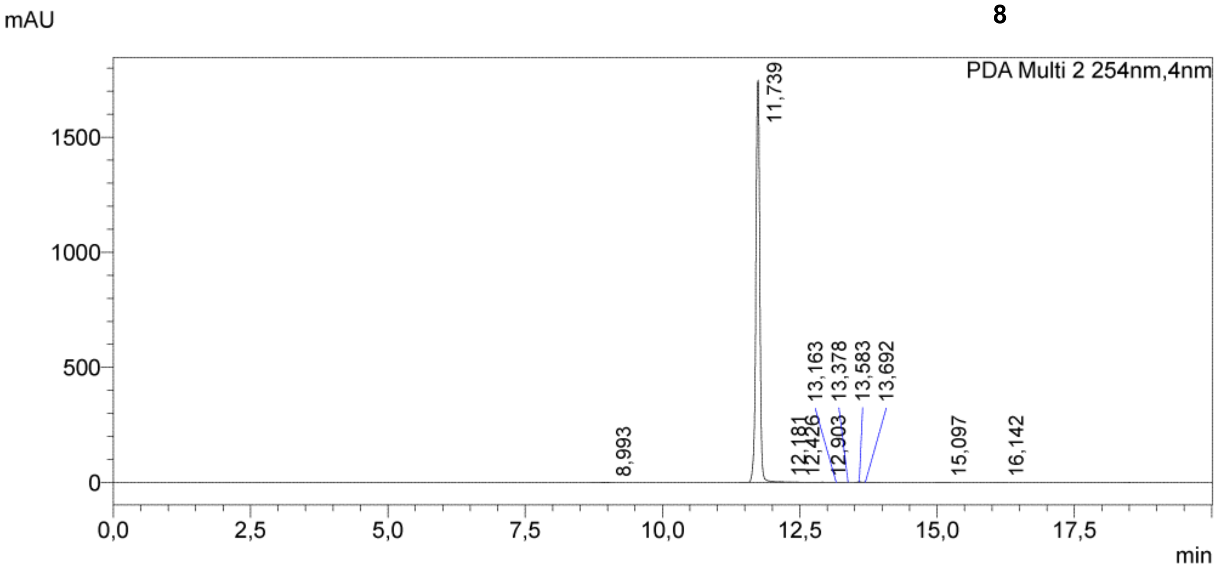

PDA Ch2 254nm

Peak#	Ret. Time	Area	Area%	Height
1	8,993	2424	0,028	241
2	11,739	8738237	99,513	1747686
3	12,181	2026	0,023	577
4	12,426	4705	0,054	1210
5	12,903	4929	0,056	1338
6	13,163	2244	0,026	189
7	13,378	5418	0,062	968
8	13,583	11252	0,128	2715
9	13,692	6901	0,079	1628
10	15,097	1462	0,017	251
11	16,142	1381	0,016	217
Total		8780980	100,000	1757020

Figure S3. HPLC chromatogram of compound **8**.

==== Shimadzu LabSolutions Analysis Report ====

Sample Name : SIRT8_500uM
Sample ID : SIRT8_500uM
Data Filename : SIRT8_500uM_p.lcd
Method Filename : MAGL254.lcm

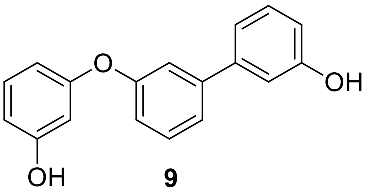

9

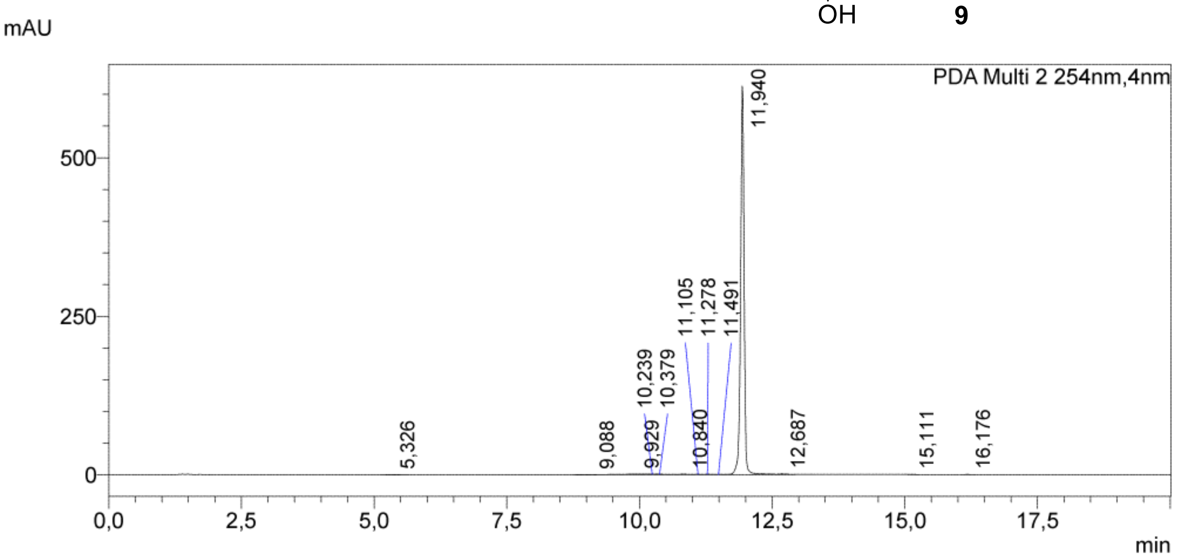

PDA Ch2 254nm

Peak#	Ret. Time	Area	Area%	Height
1	5,326	2642	0,092	212
2	9,088	6699	0,234	316
3	9,929	26135	0,915	965
4	10,239	19642	0,687	941
5	10,379	10825	0,379	839
6	10,840	11688	0,409	895
7	11,105	8486	0,297	601
8	11,278	4918	0,172	568
9	11,491	6482	0,227	489
10	11,940	2753762	96,367	612386
11	12,687	3134	0,110	695
12	15,111	2032	0,071	357
13	16,176	1145	0,040	182
Total		2857589	100,000	619448

Figure S4. HPLC chromatogram of compound **9**.

==== Shimadzu LabSolutions Analysis Report ====

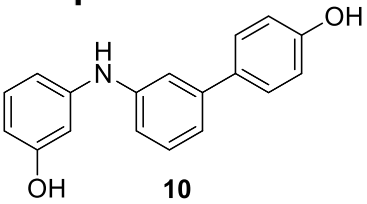

10

Sample Name : SIRT10_500uM
Sample ID : SIRT10_500uM
Data Filename : SIRT10_500uM_p.lcd
Method Filename : MAGL254.lcm

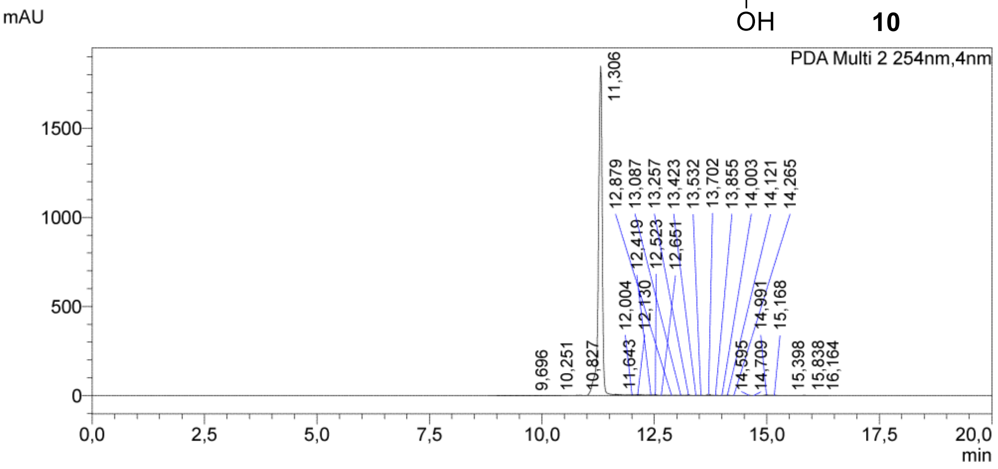

PDA Ch2 254nm

Peak#	Ret. Time	Area	Area%	Height
1	9,696	22211	0,194	577
2	10,251	31462	0,275	982
3	10,827	29595	0,259	2109
4	11,306	11139235	97,415	1847597
5	11,643	6368	0,056	1618
6	12,004	4836	0,042	968
7	12,130	18964	0,166	2967
8	12,419	9900	0,087	2099
9	12,523	15219	0,133	4025
10	12,651	1609	0,014	373
11	12,879	7005	0,061	984
12	13,087	7473	0,065	1462
13	13,257	17879	0,156	3346
14	13,423	2707	0,024	670
15	13,532	4759	0,042	920
16	13,702	32486	0,284	6670
17	13,855	6288	0,055	1162
18	14,003	2443	0,021	481
19	14,121	10606	0,093	2006
20	14,265	4142	0,036	448

Peak#	Ret. Time	Area	Area%	Height
21	14,595	5077	0,044	976
22	14,709	2948	0,026	372
23	14,991	24771	0,217	4415
24	15,168	11263	0,098	1796
25	15,398	3341	0,029	621
26	15,838	10551	0,092	1060
27	16,164	1646	0,014	210
Total		11434785	100,000	1890912

Figure S5. HPLC chromatogram of compound **10**.

==== Shimadzu LabSolutions Analysis Report ====

Sample Name : VDL18_500uM
Sample ID : VDL18_500uM
Data Filename : VDL18_500uM_p.lcd
Method Filename : MAGL254.lcm

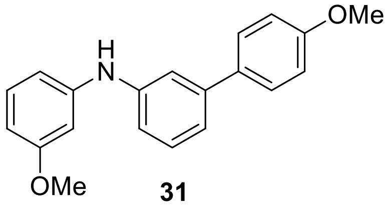

31

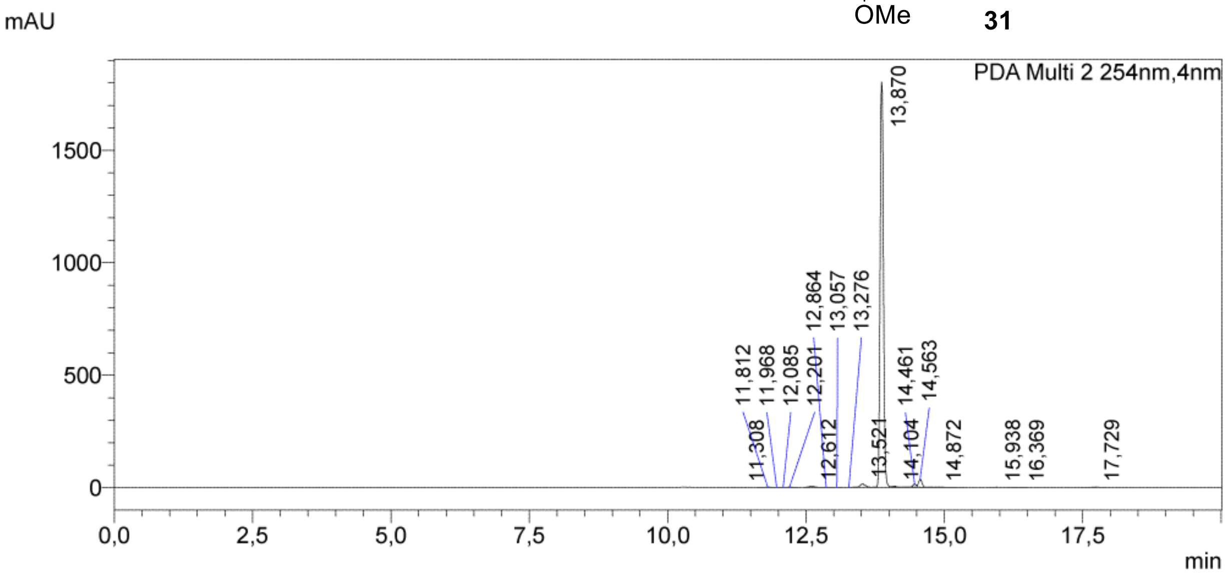

PDA Ch2 254nm

Peak#	Ret. Time	Area	Area%	Height
1	11,308	1173	0,014	198
2	11,812	4720	0,057	416
3	11,968	1792	0,022	300
4	12,085	1817	0,022	314
5	12,201	3673	0,044	459
6	12,612	32564	0,393	3749
7	12,864	2834	0,034	841
8	13,057	2029	0,025	348
9	13,276	4097	0,049	553
10	13,521	111850	1,351	15976
11	13,870	7853316	94,837	1802821
12	14,104	7653	0,092	2518
13	14,461	67404	0,814	15107
14	14,563	156359	1,888	36385
15	14,872	8334	0,101	661
16	15,938	5411	0,065	709
17	16,369	3039	0,037	286
18	17,729	12829	0,155	1667
Total		8280893	100,000	1883307

Figure S6. HPLC chromatogram of compound **31**.

==== Shimadzu LabSolutions Analysis Report ====

Sample Name : VDL34_500uM
Sample ID : VDL34_500uM
Data Filename : VDL34_500uM_p_c.lcd
Method Filename : MAGL254.lcm

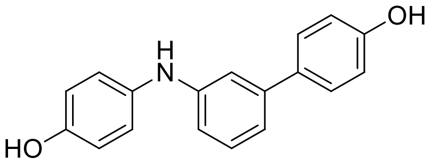

11

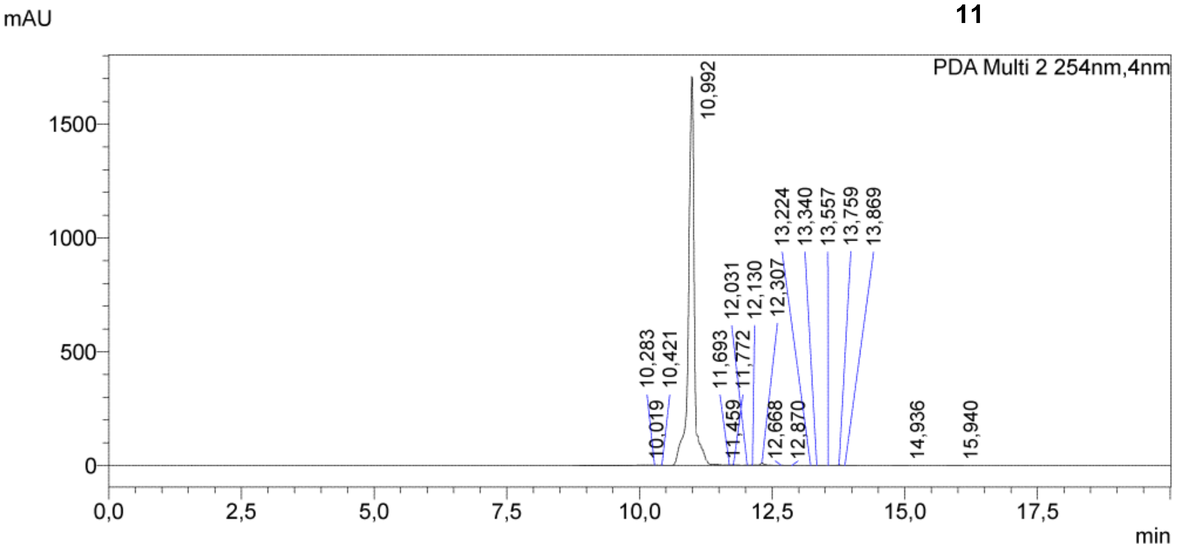

PDA Ch2 254nm

Peak#	Ret. Time	Area	Area%	Height
1	10,019	59782	0,418	1843
2	10,283	18605	0,130	1374
3	10,421	11264	0,079	1271
4	10,992	14094126	98,626	1708369
5	11,459	3379	0,024	767
6	11,693	4165	0,029	903
7	11,772	4683	0,033	1190
8	12,031	1949	0,014	421
9	12,130	3699	0,026	629
10	12,307	59221	0,414	10802
11	12,668	4037	0,028	647
12	12,870	1746	0,012	274
13	13,224	2758	0,019	461
14	13,340	1531	0,011	213
15	13,557	1002	0,007	125
16	13,759	9547	0,067	2150
17	13,869	2960	0,021	560
18	14,936	3696	0,026	479
19	15,940	2369	0,017	400
Total		14290520	100,000	1732879

Figure S7. HPLC chromatogram of compound **11**.

==== Shimadzu LabSolutions Analysis Report ====

Sample Name : VDL32A_500uM
Sample ID : VDL32A_500uM
Data Filename : VDL32A_500uM_p_c.lcd
Method Filename : MAGL254.lcm

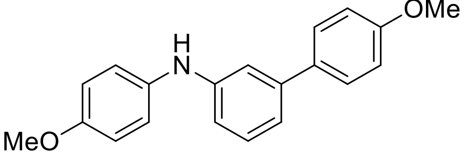

32

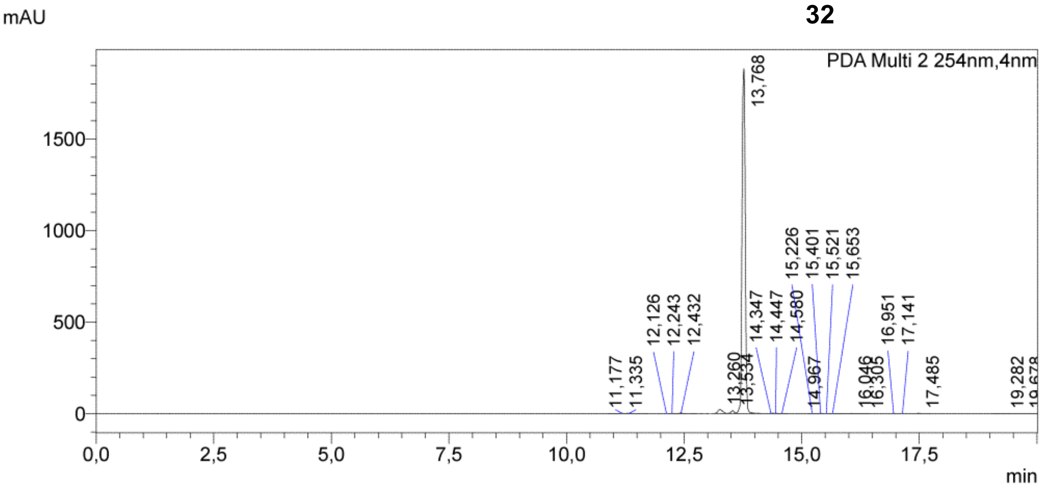

PDA Ch2 254nm

Peak#	Ret. Time	Area	Area%	Height
1	11,177	10660	0,118	1527
2	11,335	14198	0,158	1994
3	12,126	3337	0,037	478
4	12,243	3022	0,034	466
5	12,432	13699	0,152	2890
6	13,260	156508	1,737	21002
7	13,534	83433	0,926	15931
8	13,768	8644680	95,957	1881584
9	14,347	6408	0,071	1641
10	14,447	8735	0,097	1930
11	14,580	4740	0,053	848
12	14,967	3547	0,039	422
13	15,226	1060	0,012	261
14	15,401	4703	0,052	902
15	15,521	3199	0,036	622
16	15,653	5143	0,057	702
17	16,046	7823	0,087	898
18	16,305	4245	0,047	539
19	16,951	8462	0,094	1103
20	17,141	1286	0,014	223

Peak#	Ret. Time	Area	Area%	Height
21	17,485	10527	0,117	1295
22	19,282	3908	0,043	304
23	19,678	5568	0,062	253
Total		9008890	100,000	1937816

Figure S8. HPLC chromatogram of compound **32**.

==== Shimadzu LabSolutions Analysis Report ====

Sample Name : SIRT12_500uM
Sample ID : SIRT12_500uM
Data Filename : SIRT12_500uM_p_corrected.lcd
Method Filename : MAGL254.lcm

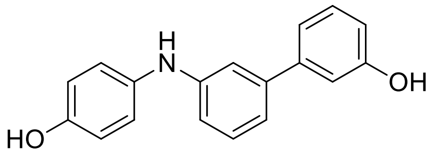

12

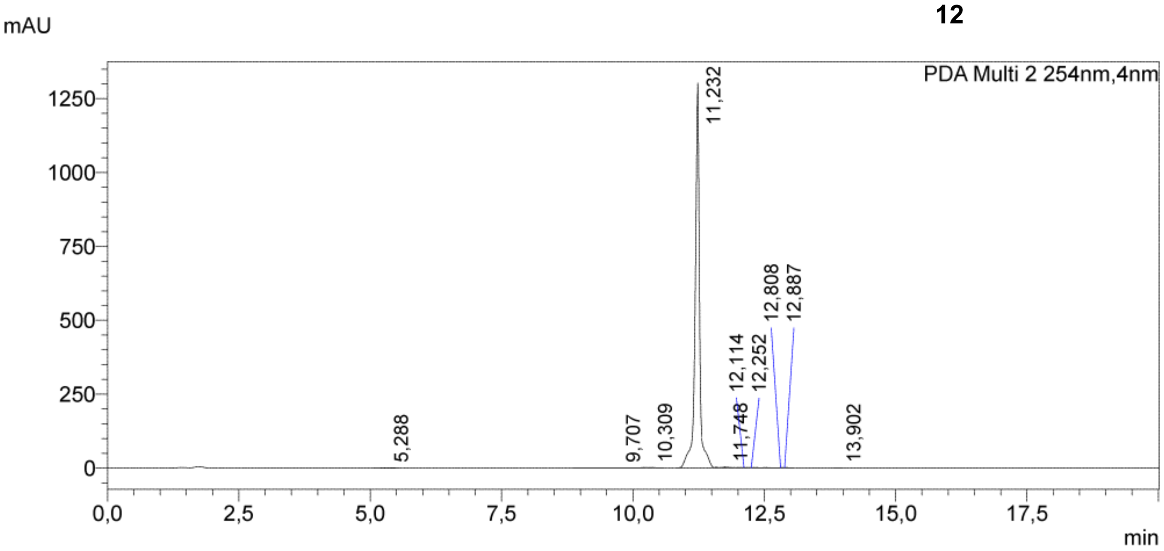

PDA Ch2 254nm

Peak#	Ret. Time	Area	Area%	Height
1	5,288	1753	0,022	139
2	9,707	13033	0,162	295
3	10,309	11737	0,146	488
4	11,232	8007796	99,438	1301233
5	11,748	7669	0,095	1453
6	12,114	4595	0,057	1022
7	12,252	2260	0,028	386
8	12,808	1116	0,014	284
9	12,887	2017	0,025	309
10	13,902	1067	0,013	211
Total		8053043	100,000	1305821

Figure S9. HPLC chromatogram of compound **12**.

==== Shimadzu LabSolutions Analysis Report ====

Sample Name : SIRT13_500uM
Sample ID : SIRT13_500uM
Data Filename : SIRT13_500uM_p.lcd
Method Filename : MAGL254.lcm

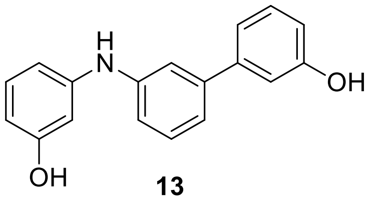

13

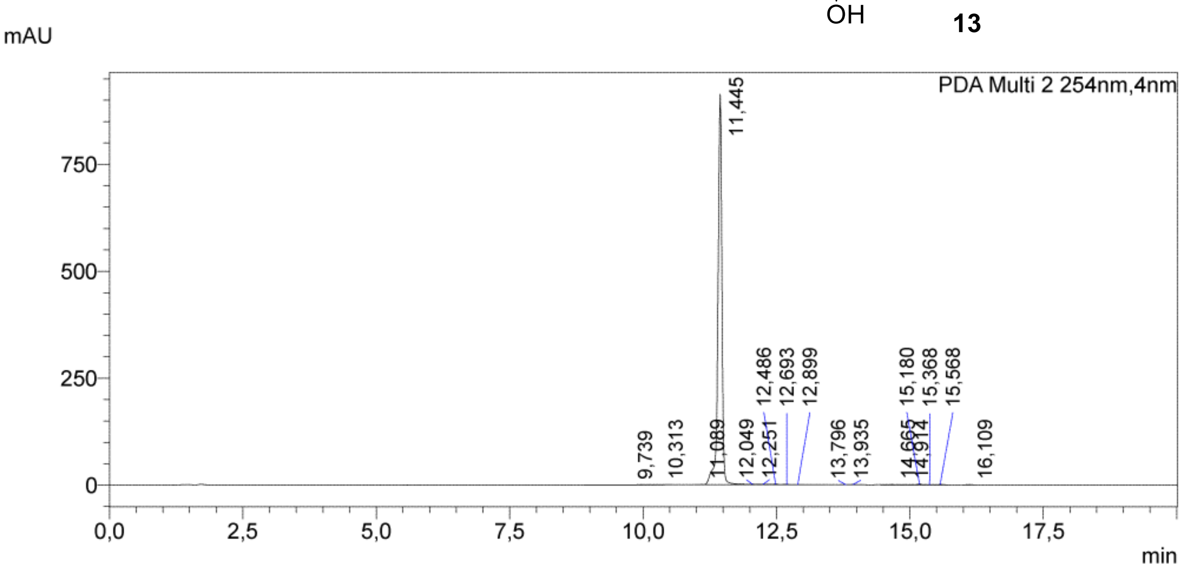

PDA Ch2 254nm

Peak#	Ret. Time	Area	Area%	Height
1	9,739	8658	0,180	210
2	10,313	11690	0,244	498
3	11,089	3951	0,082	799
4	11,445	4712803	98,234	912790
5	12,049	2273	0,047	410
6	12,251	4332	0,090	709
7	12,486	6158	0,128	1263
8	12,693	4436	0,092	811
9	12,899	2834	0,059	530
10	13,796	2195	0,046	474
11	13,935	7274	0,152	1310
12	14,665	5973	0,124	803
13	14,914	2350	0,049	294
14	15,180	10687	0,223	1807
15	15,368	4321	0,090	626
16	15,568	5861	0,122	1012
17	16,109	1730	0,036	295
Total		4797526	100,000	924639

Figure S10. HPLC chromatogram of compound **13**.

==== Shimadzu LabSolutions Analysis Report ====

Sample Name : SIRT31_500uM
Sample ID : SIRT31_500uM
Data Filename : SIRT31_500uM_p.lcd
Method Filename : MAGL254.lcm

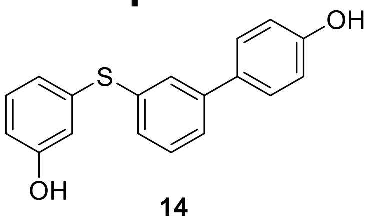

14

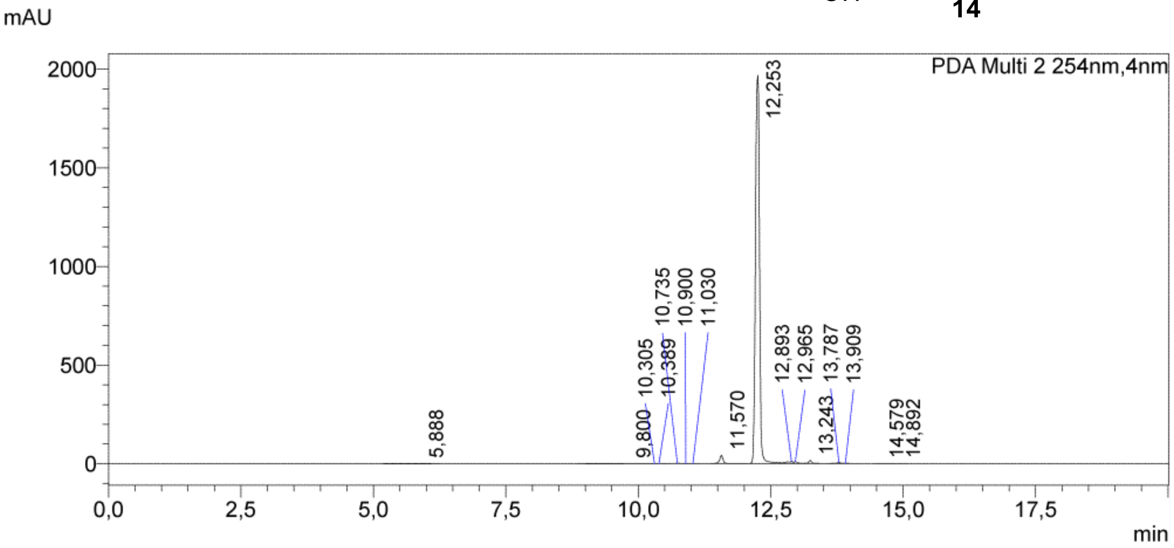

PDA Ch2 254nm

Peak#	Ret. Time	Area	Area%	Height
1	5,888	32543	0,302	1176
2	9,800	21862	0,203	1065
3	10,305	13691	0,127	794
4	10,389	7696	0,071	724
5	10,735	11680	0,108	1667
6	10,900	3696	0,034	694
7	11,030	1563	0,014	235
8	11,570	199247	1,847	41374
9	12,253	10216796	94,731	1966938
10	12,893	92426	0,857	10114
11	12,965	51608	0,479	9414
12	13,243	80790	0,749	15871
13	13,787	35525	0,329	5267
14	13,909	10991	0,102	1177
15	14,579	3331	0,031	363
16	14,892	1637	0,015	303
Total		10785080	100,000	2057177

Figure S11. HPLC chromatogram of compound **14**.

==== Shimadzu LabSolutions Analysis Report ====

Sample Name : SIRT29_500uM
Sample ID : SIRT29_500uM
Data Filename : SIRT29_500uM_p.lcd
Method Filename : MAGL254.lcm

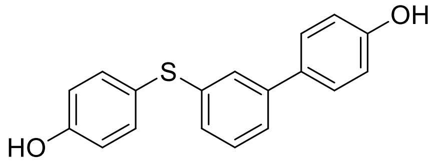

15

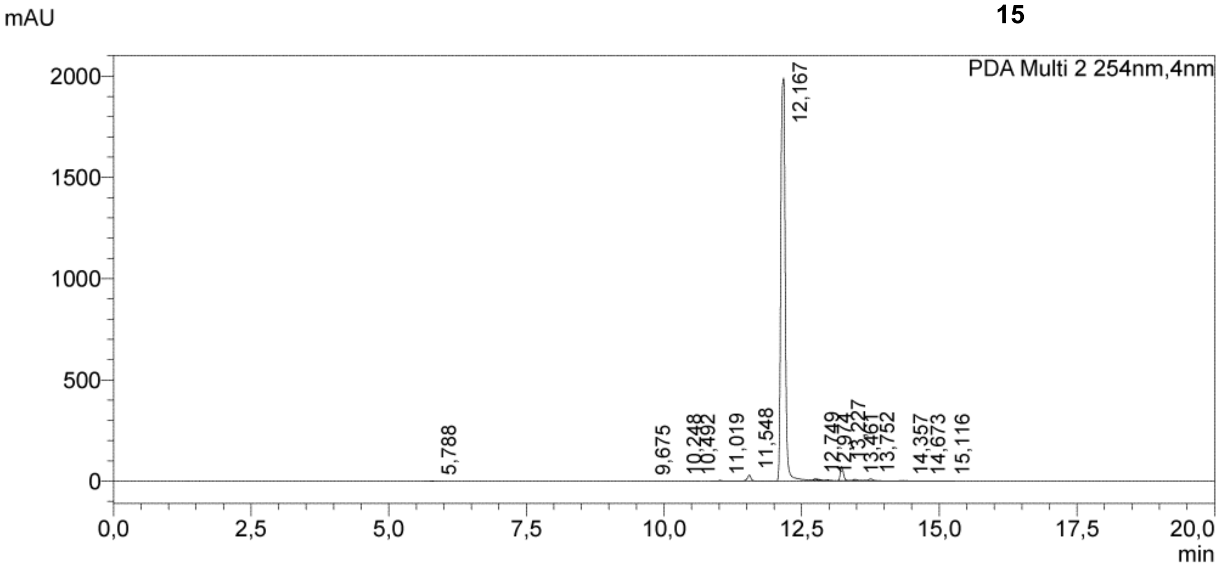

PDA Ch2 254nm

Peak#	Ret. Time	Area	Area%	Height
1	5,788	1532	0,013	165
2	9,675	1602	0,013	99
3	10,248	7486	0,061	499
4	10,492	8369	0,068	844
5	11,019	17847	0,146	2757
6	11,548	147973	1,209	29928
7	12,167	11607029	94,861	1989564
8	12,749	36429	0,298	6269
9	12,974	9784	0,080	2938
10	13,227	267323	2,185	70528
11	13,461	54685	0,447	6994
12	13,752	57006	0,466	10096
13	14,357	14130	0,115	802
14	14,673	2446	0,020	398
15	15,116	2149	0,018	398
Total		12235792	100,000	2122278

Figure S12. HPLC chromatogram of compound **15**.

==== Shimadzu LabSolutions Analysis Report ====

Sample Name : SIRT30_500uM
Sample ID : SIRT30_500uM
Data Filename : SIRT30_500uM_p.lcd
Method Filename : MAGL254.lcm

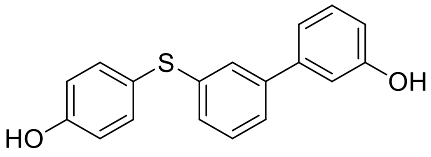

16

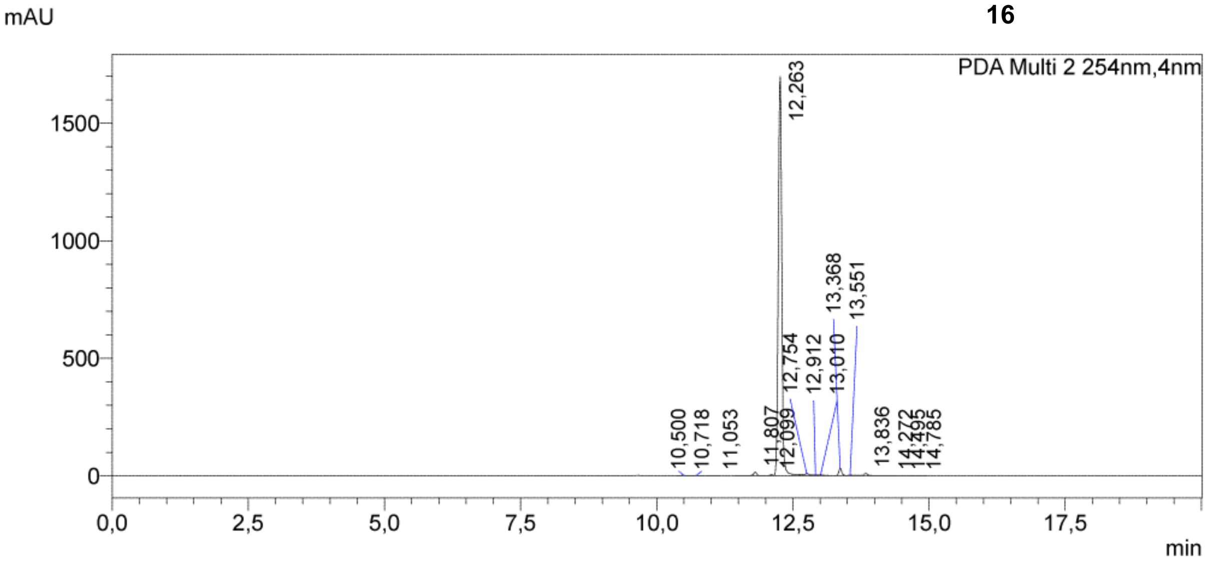

PDA Ch2 254nm

Peak#	Ret. Time	Area	Area%	Height
1	10,500	5193	0,066	934
2	10,718	1929	0,025	254
3	11,053	1175	0,015	163
4	11,807	65903	0,837	15005
5	12,099	15300	0,194	3539
6	12,263	7446913	94,625	1697377
7	12,754	67255	0,855	9336
8	12,912	17924	0,228	3228
9	13,010	29405	0,374	3817
10	13,368	125937	1,600	32255
11	13,551	24570	0,312	2445
12	13,836	62711	0,797	11555
13	14,272	1342	0,017	126
14	14,495	2890	0,037	303
15	14,785	1433	0,018	232
Total		7869881	100,000	1780570

Figure S13. HPLC chromatogram of compound **16**.

==== Shimadzu LabSolutions Analysis Report ====

Sample Name : CCA20_500uM
Sample ID : CCA20_500uM
Data Filename : CCA20_500uM_p_c.lcd
Method Filename : MAGL254.lcm

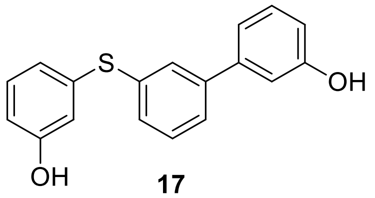

17

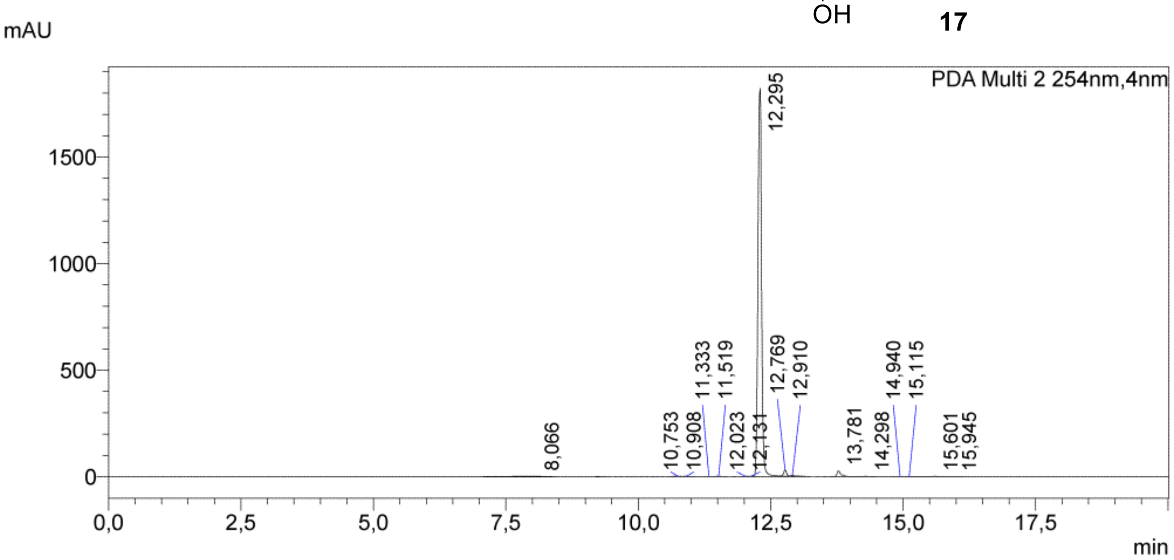

PDA Ch2 254nm

Peak#	Ret. Time	Area	Area%	Height
1	8,066	81090	0,865	2254
2	10,753	9557	0,102	1515
3	10,908	3854	0,041	749
4	11,333	1049	0,011	258
5	11,519	6999	0,075	1580
6	12,023	4124	0,044	921
7	12,131	6213	0,066	1446
8	12,295	8950411	95,505	1820648
9	12,769	120466	1,285	30536
10	12,910	22536	0,240	3903
11	13,781	140965	1,504	27496
12	14,298	6306	0,067	1311
13	14,940	3104	0,033	416
14	15,115	3455	0,037	402
15	15,601	8826	0,094	1743
16	15,945	2689	0,029	445
Total		9371643	100,000	1895622

Figure S14. HPLC chromatogram of compound **17**.

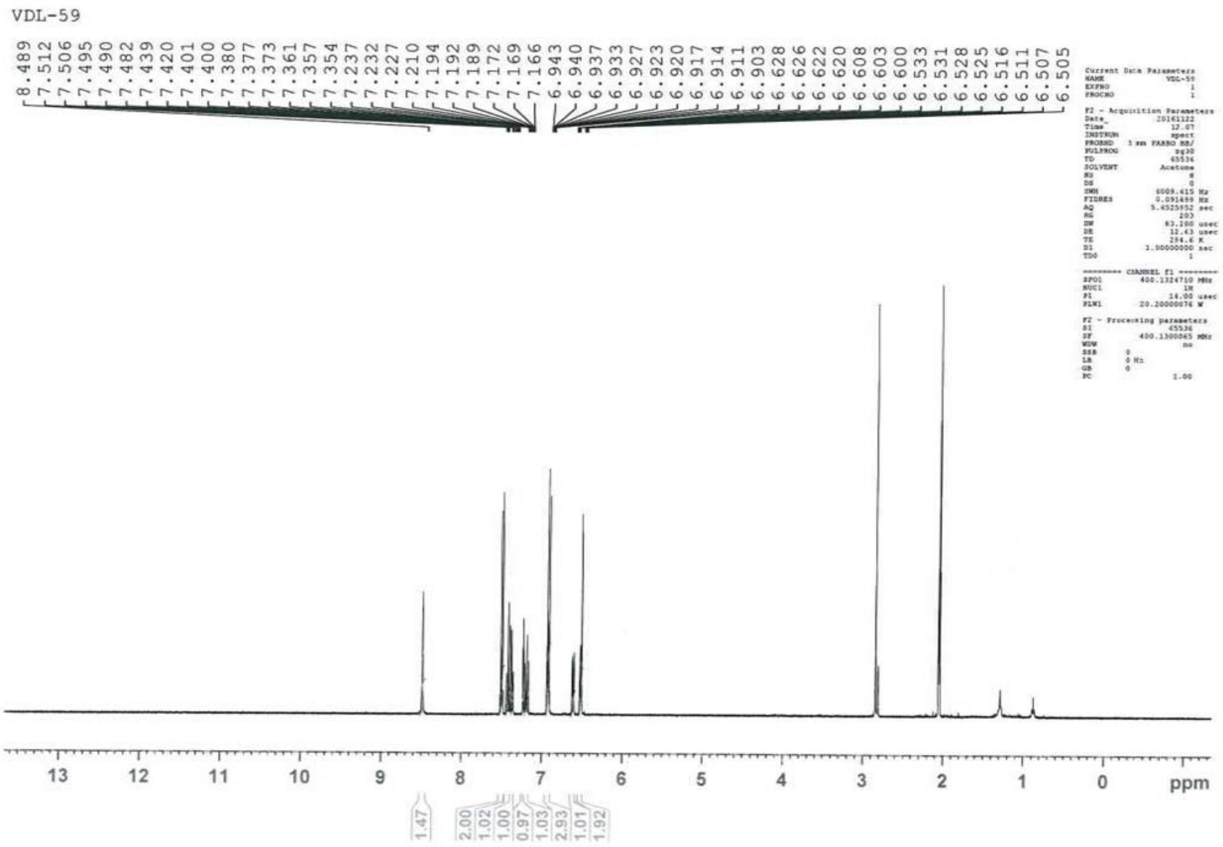

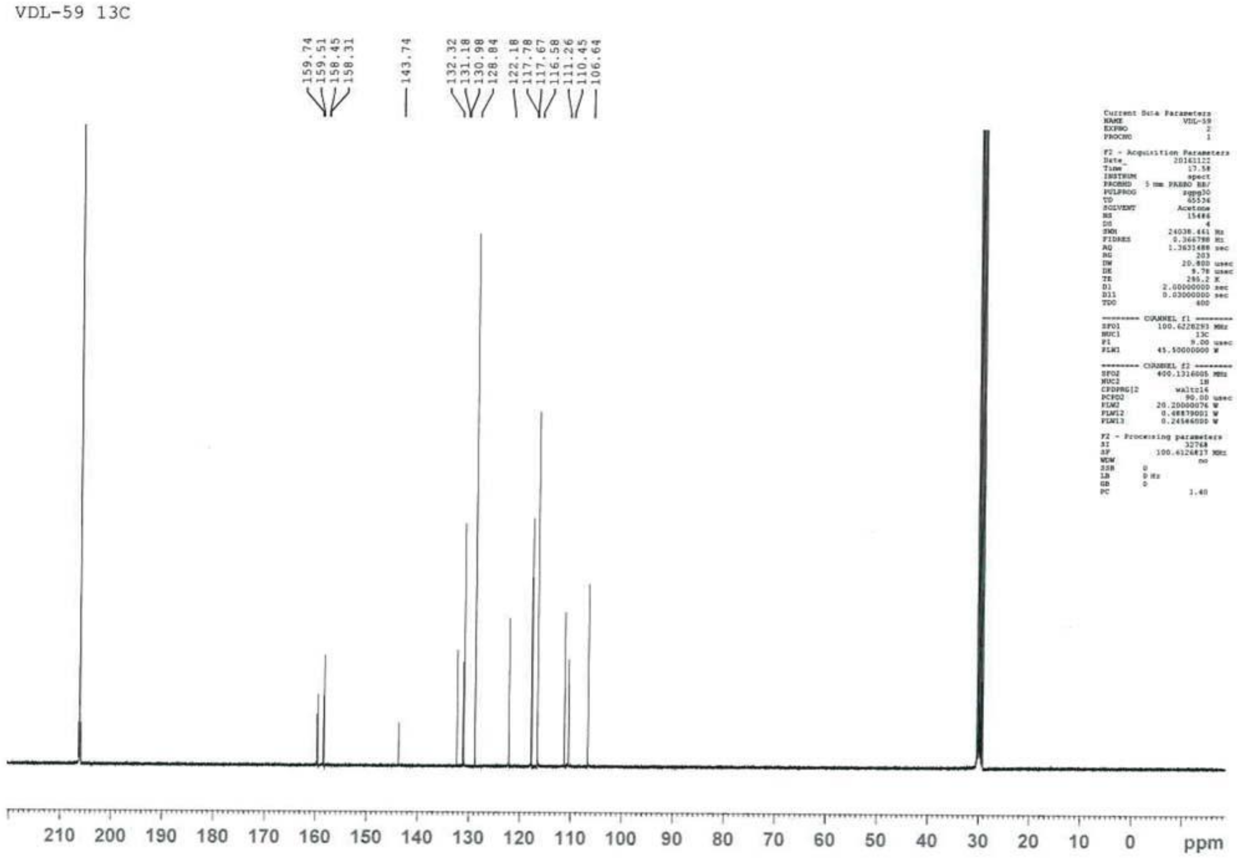

Figure S15. ^{1}H-NMR and ^{13}C-NMR of compound **6**.

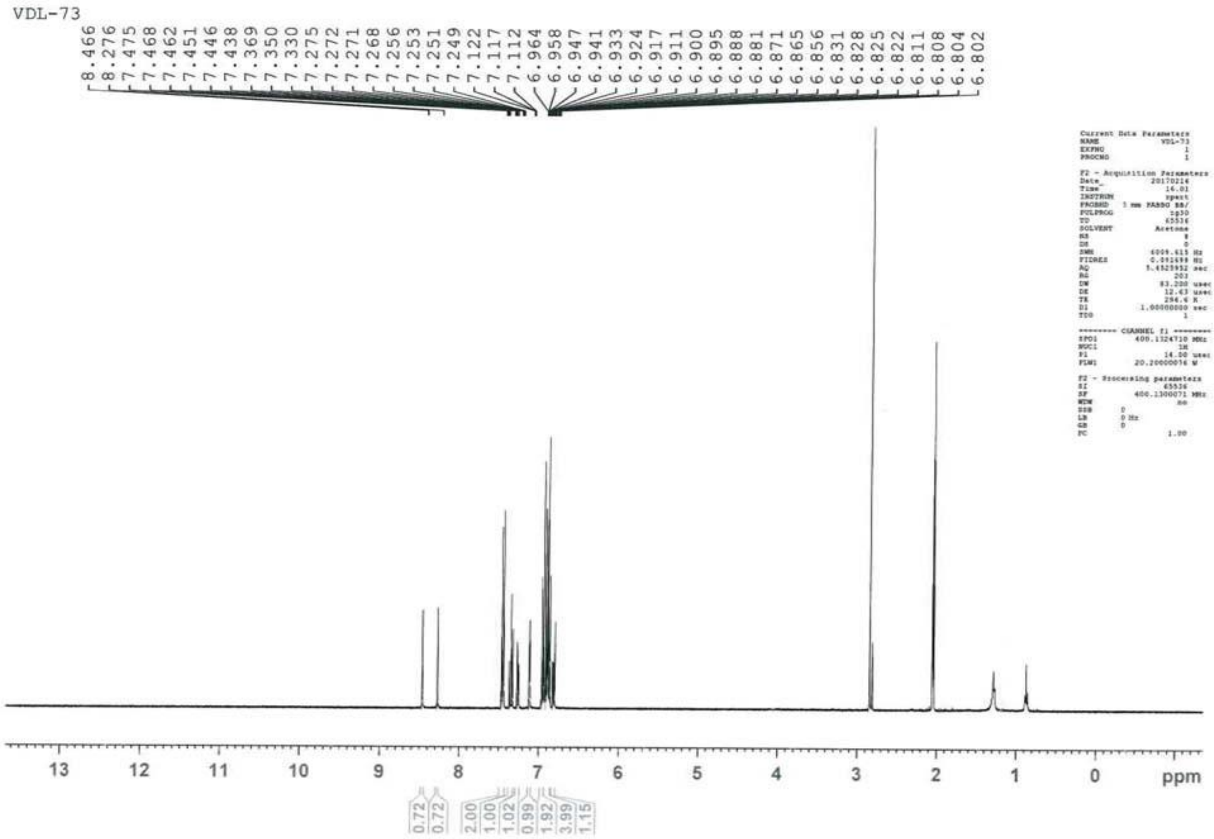

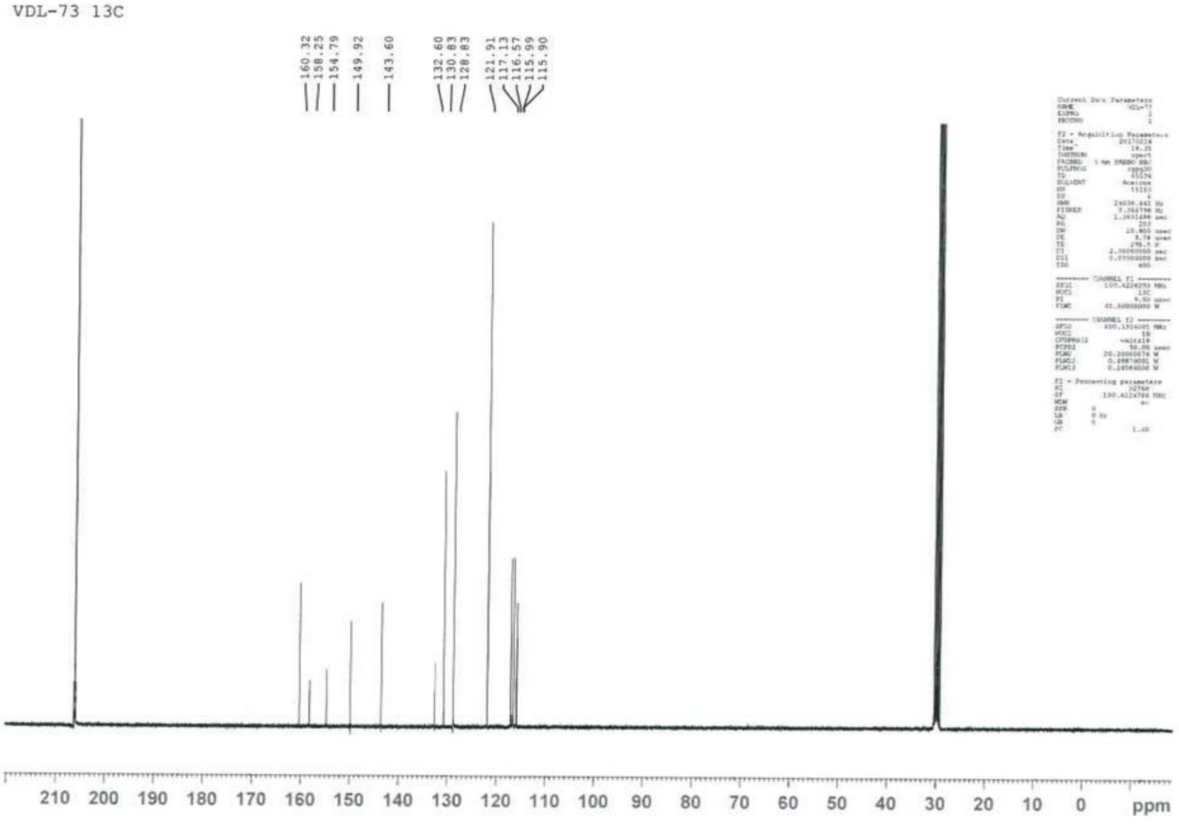

Figure S16. ^{1}H-NMR and ^{13}C-NMR of compound **7**.

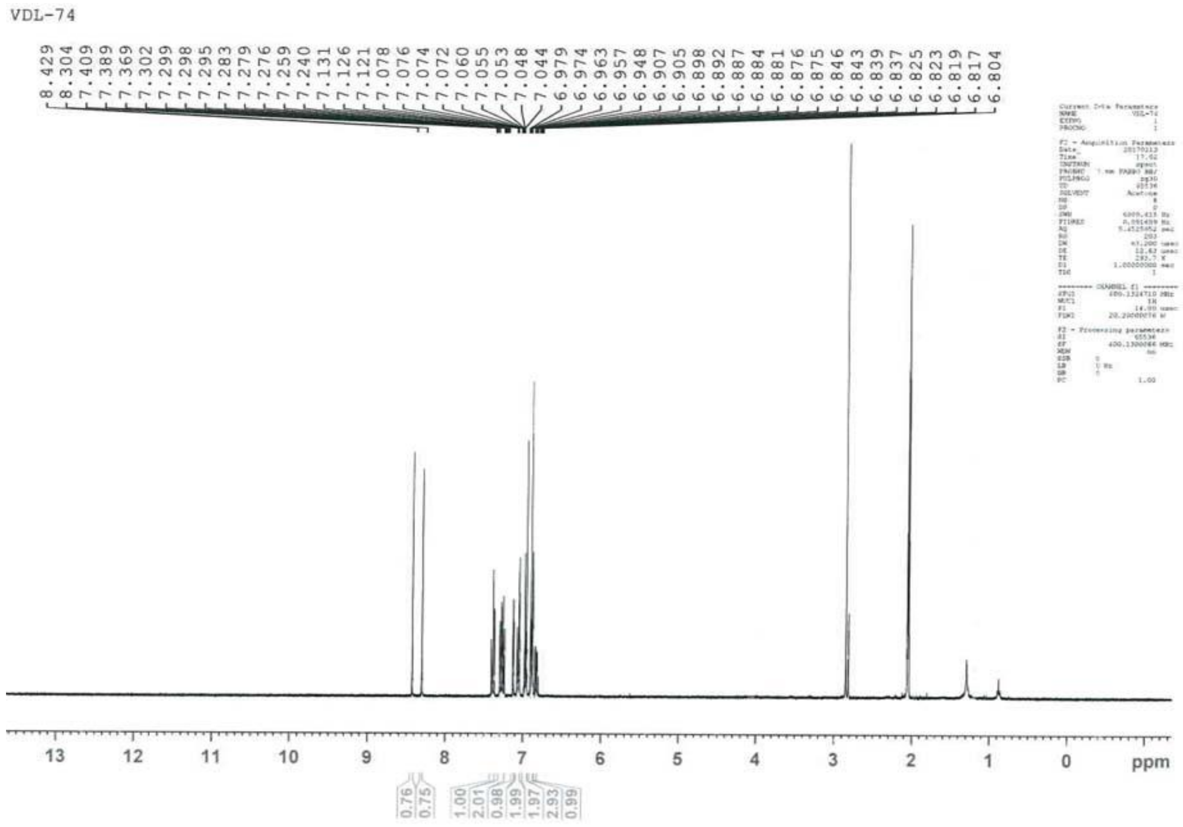

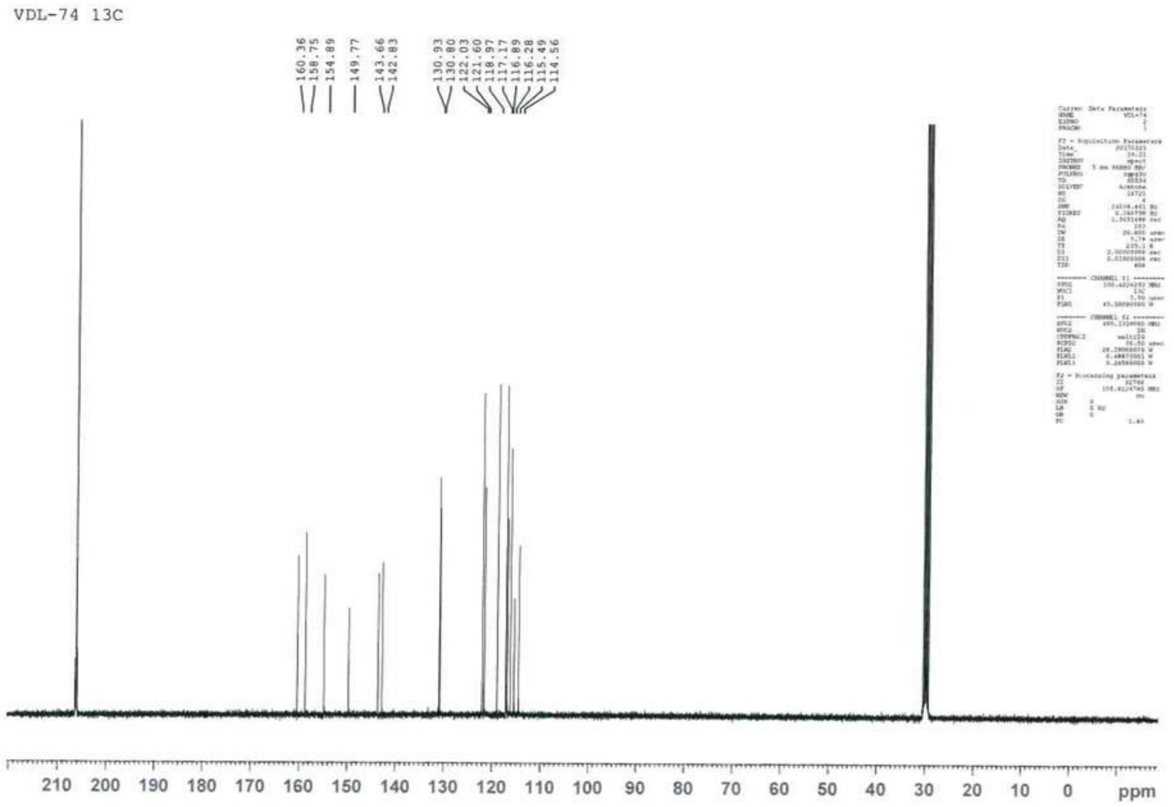

Figure S17. ^{1}H-NMR and ^{13}C-NMR of compound **8**.

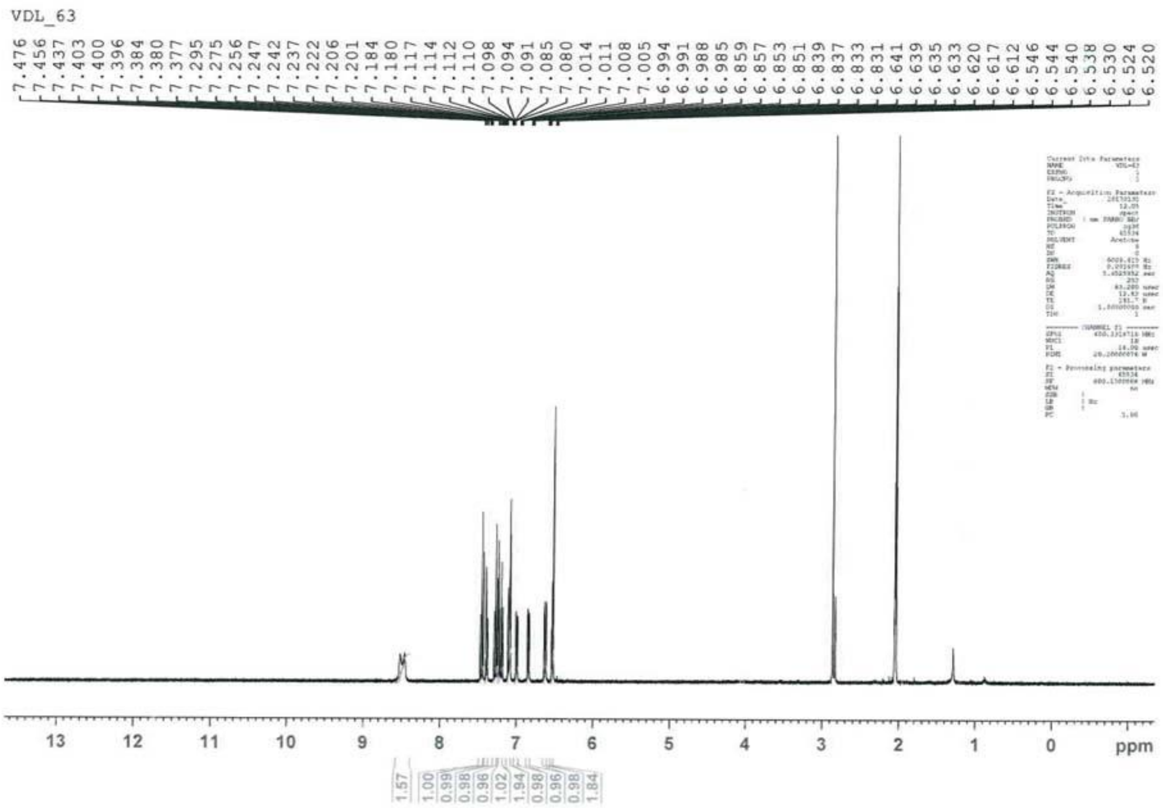

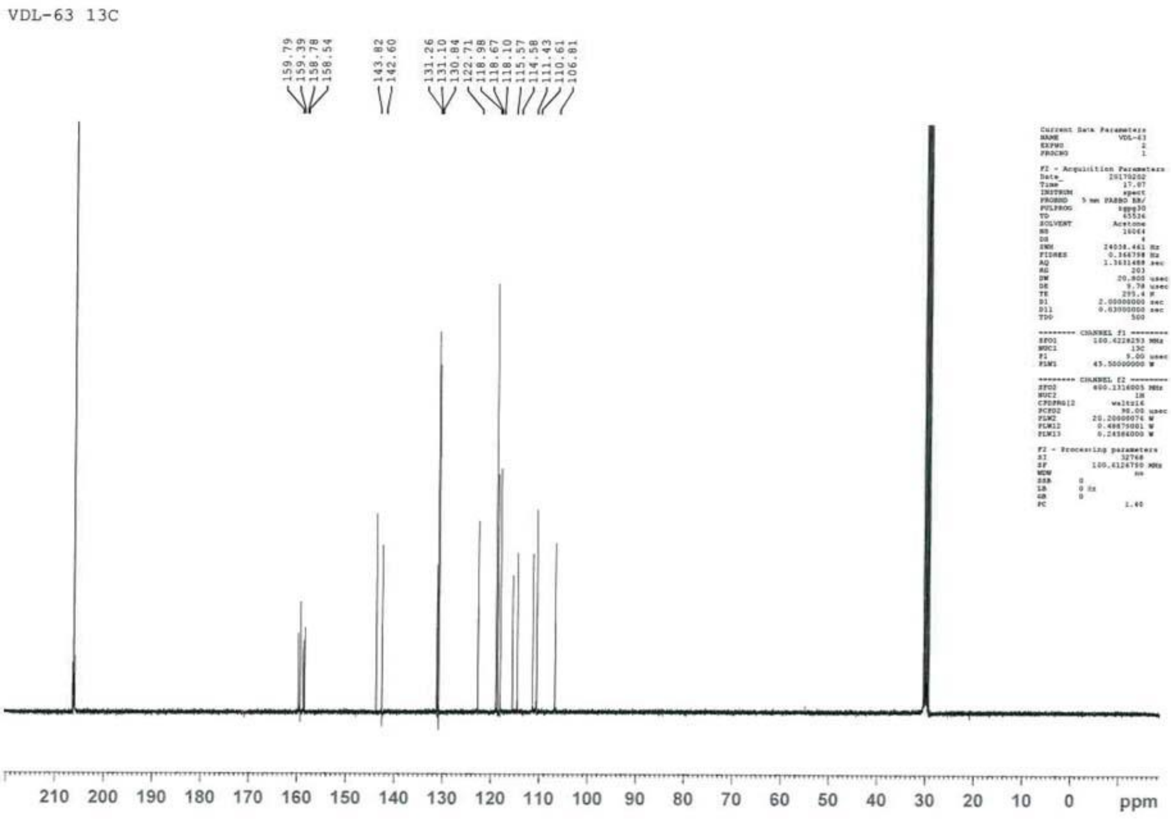

Figure S18. ^{1}H-NMR and ^{13}C-NMR of compound **9**.

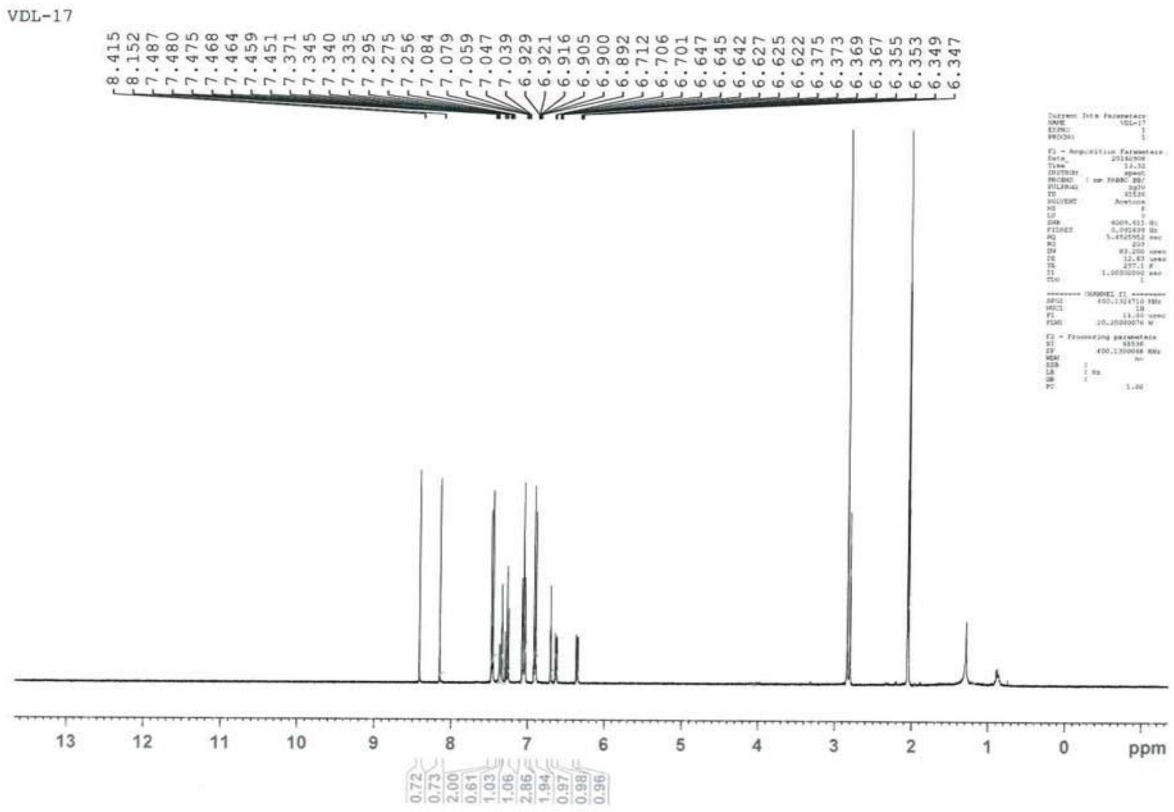

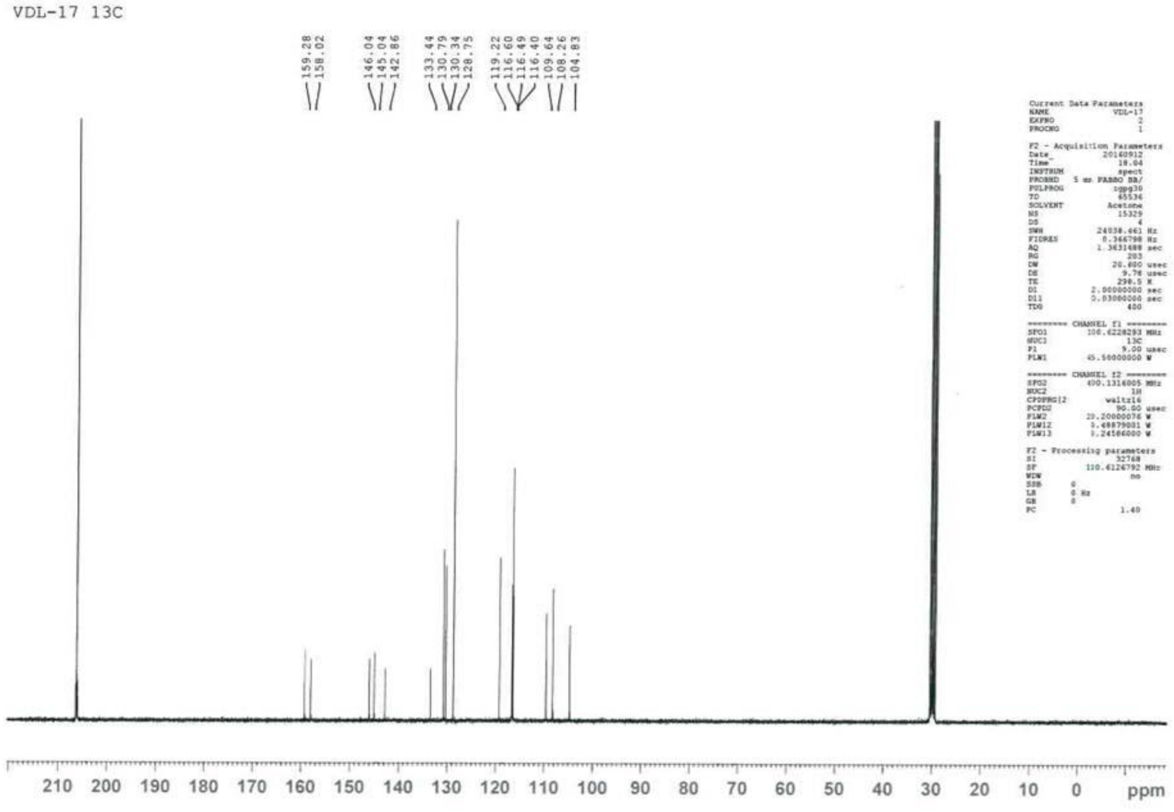

Figure S19. ^{1}H-NMR and ^{13}C-NMR of compound **10**.

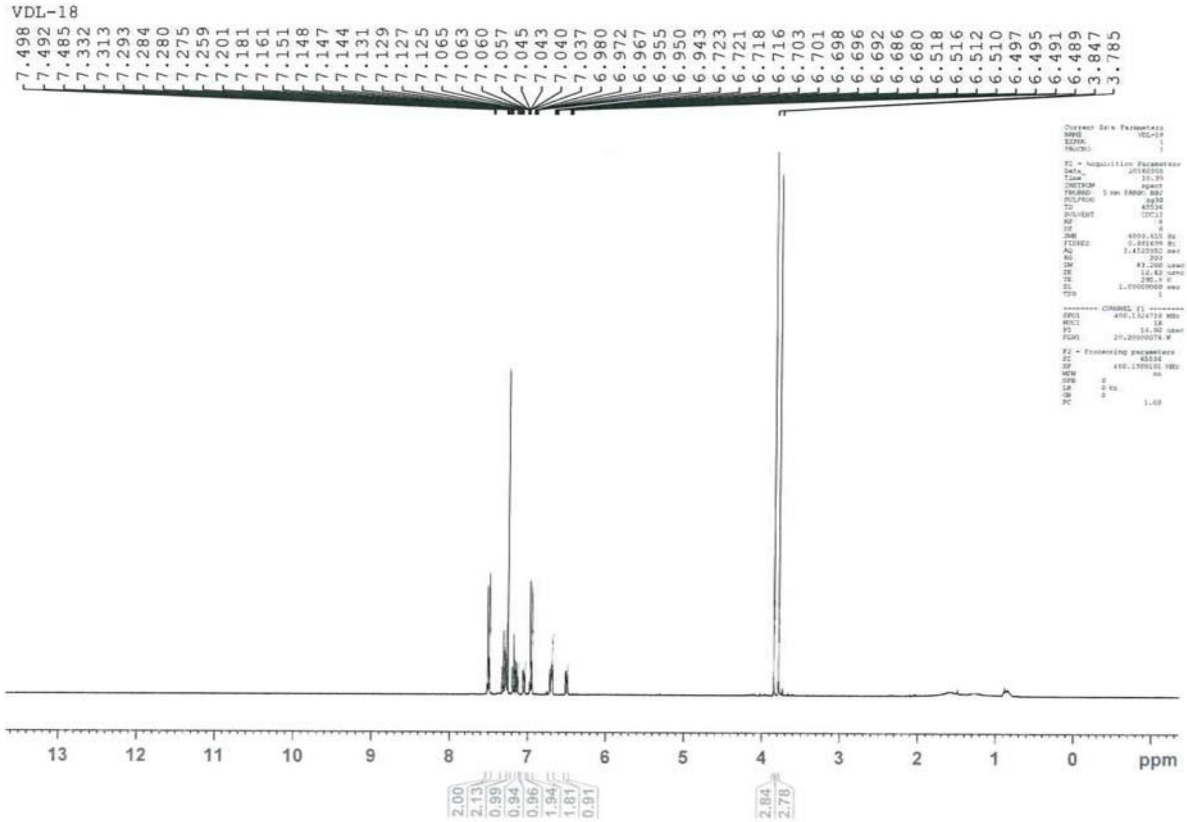

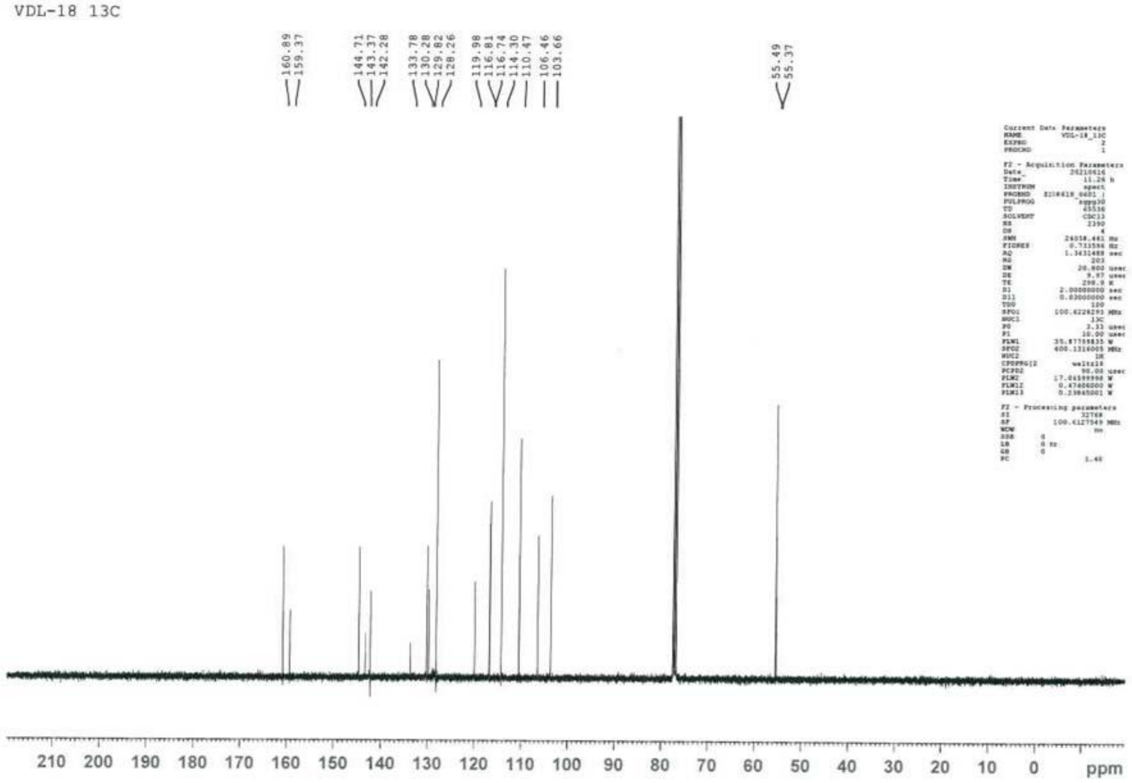

Figure S20. ^{1}H-NMR and ^{13}C-NMR of compound **31**.

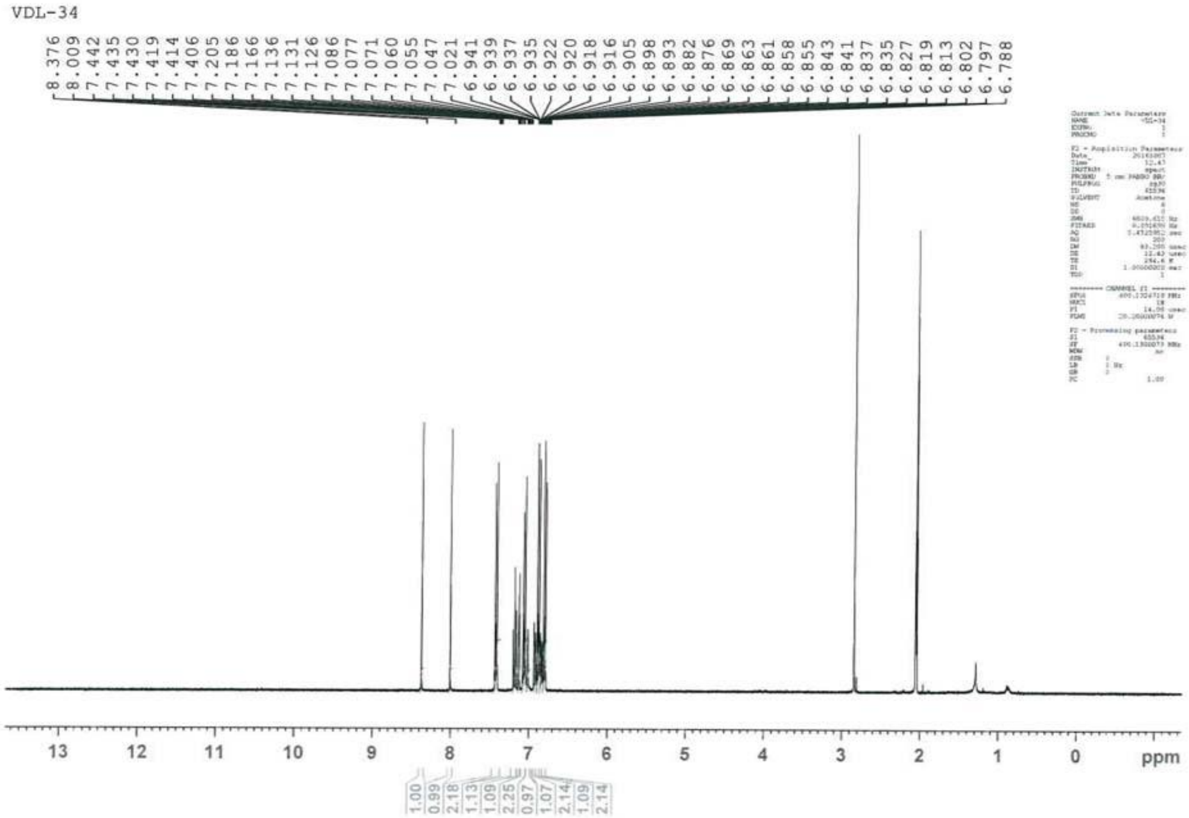

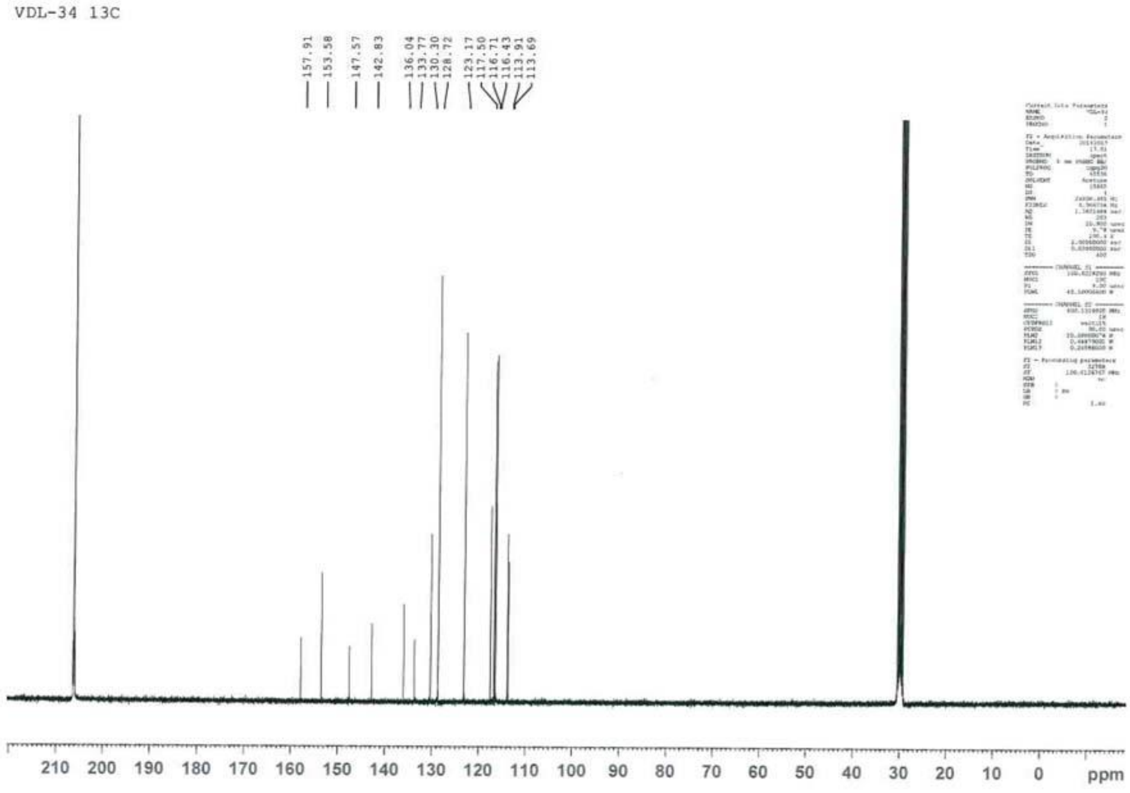

Figure S21. ^{1}H-NMR and ^{13}C-NMR of compound **11**.

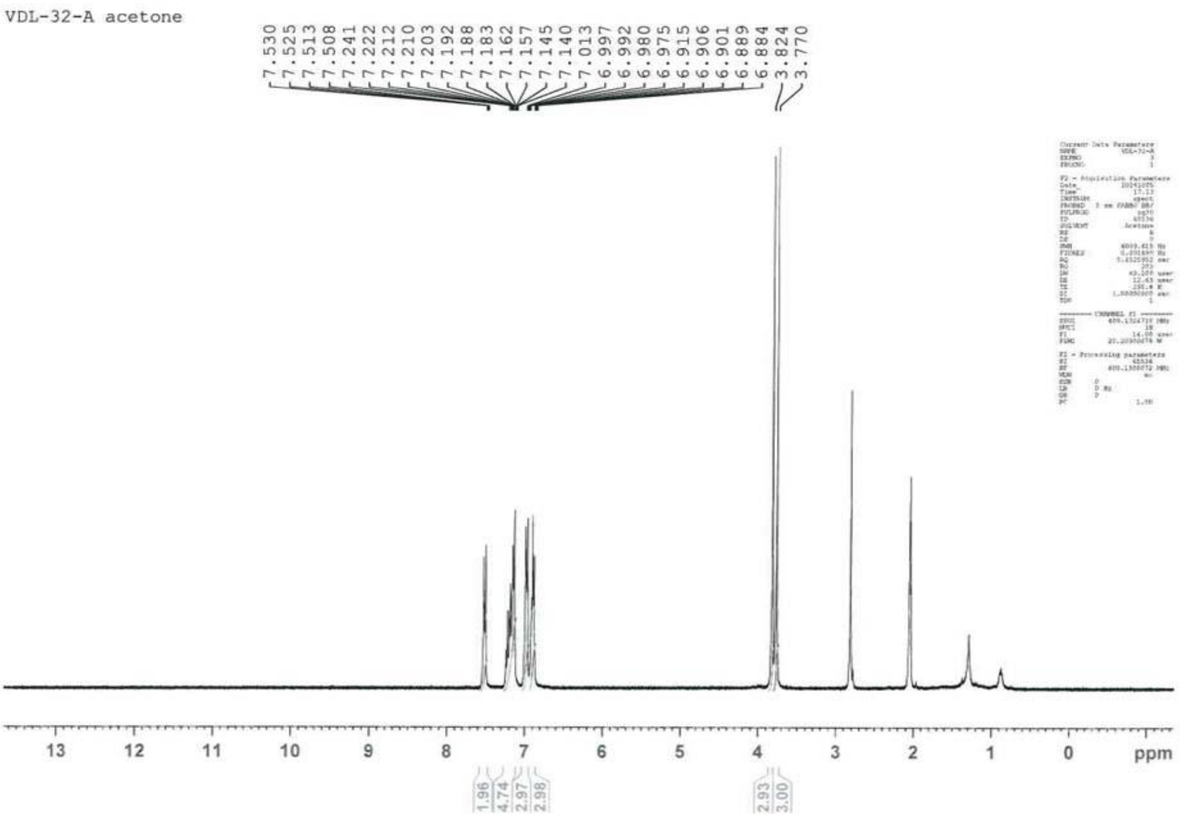

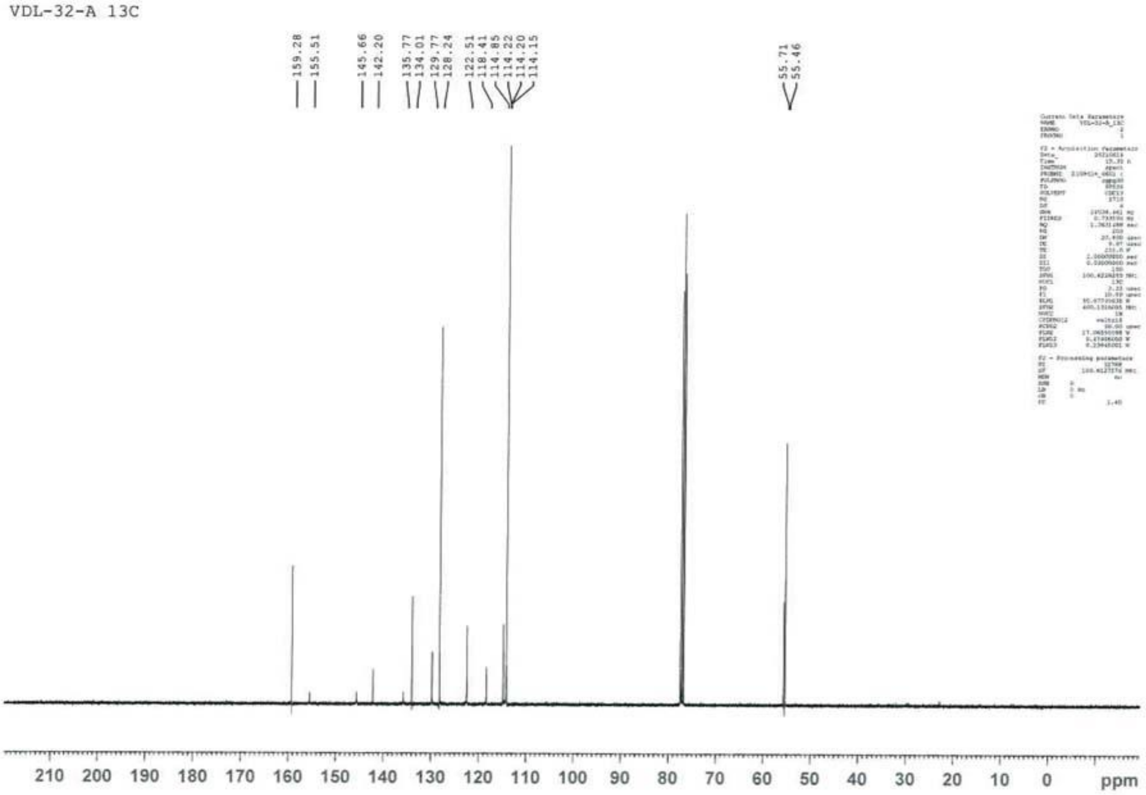

Figure S22. ^{1}H-NMR and ^{13}C-NMR of compound **32**.

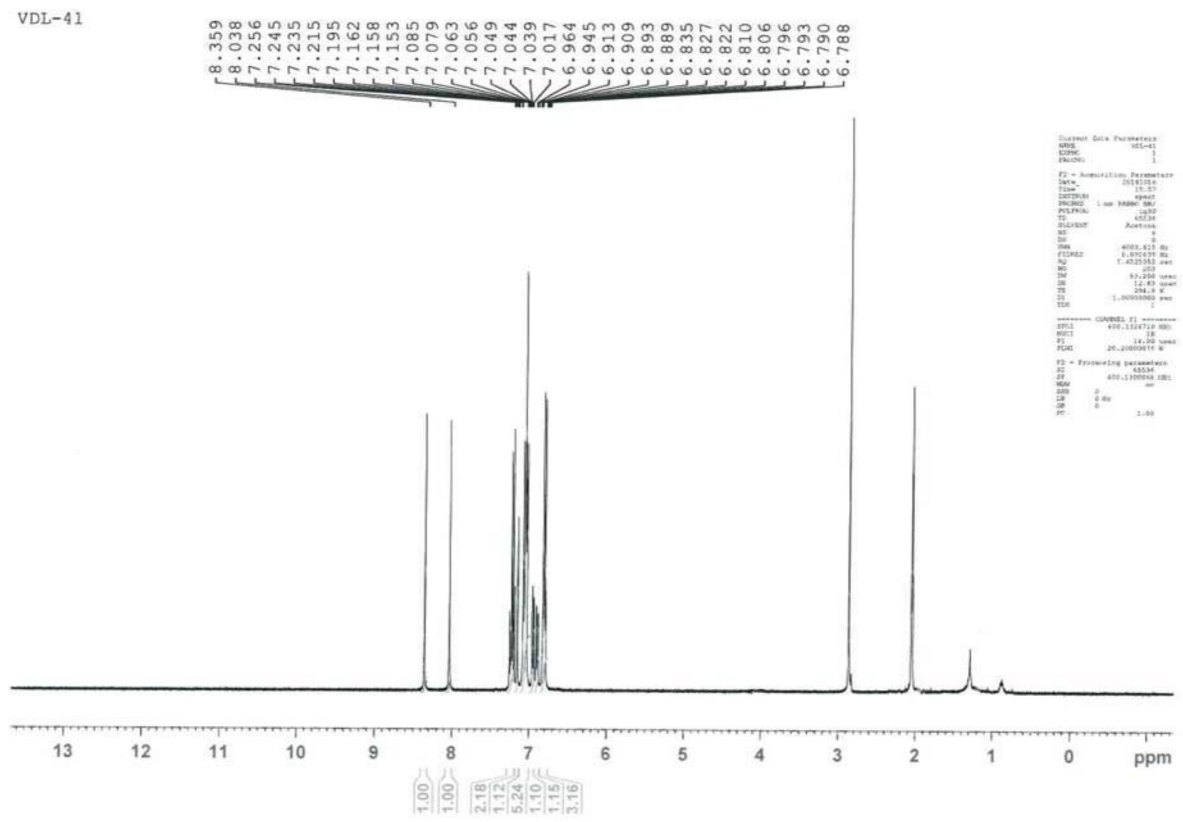

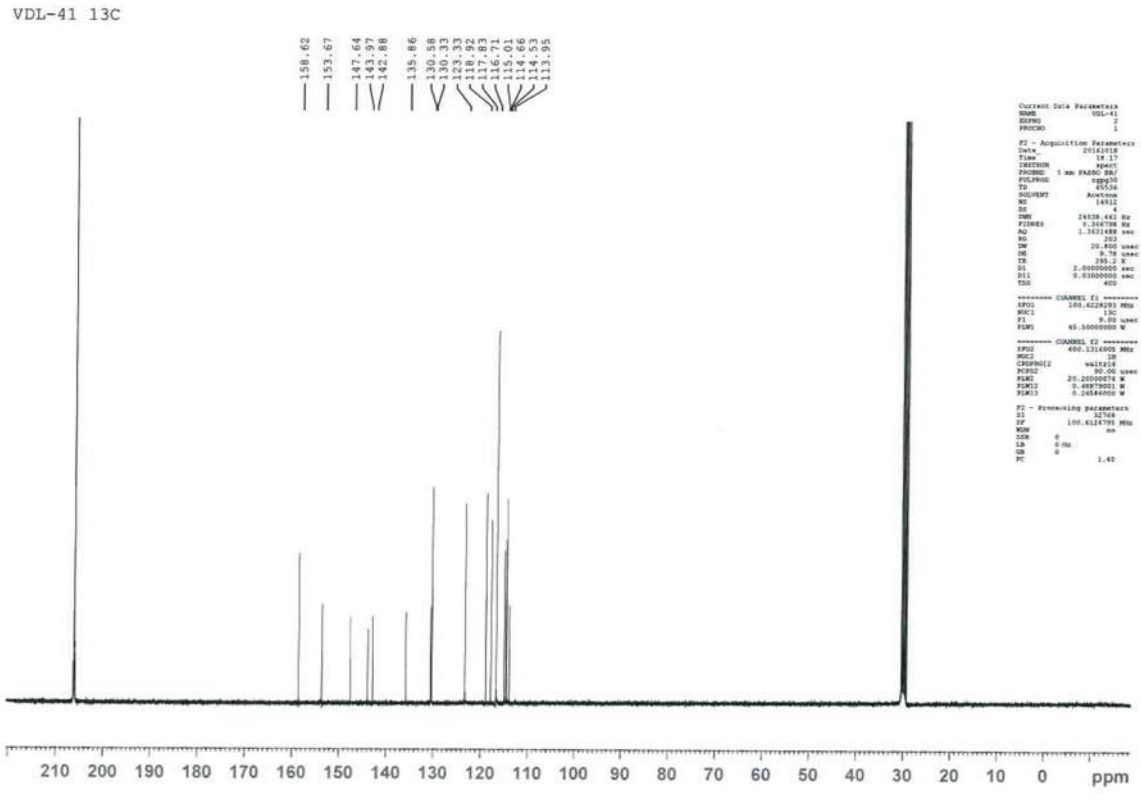

Figure S23. ^{1}H-NMR and ^{13}C-NMR of compound **12**.

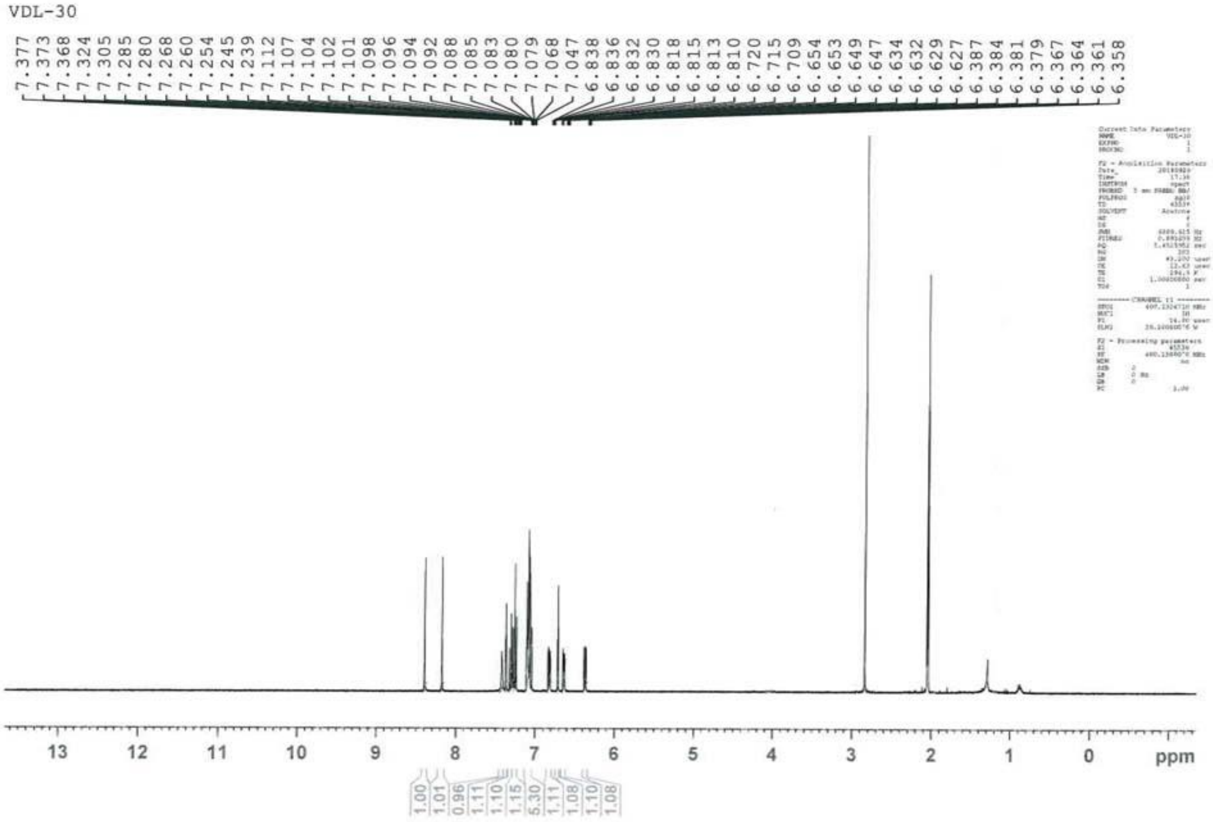

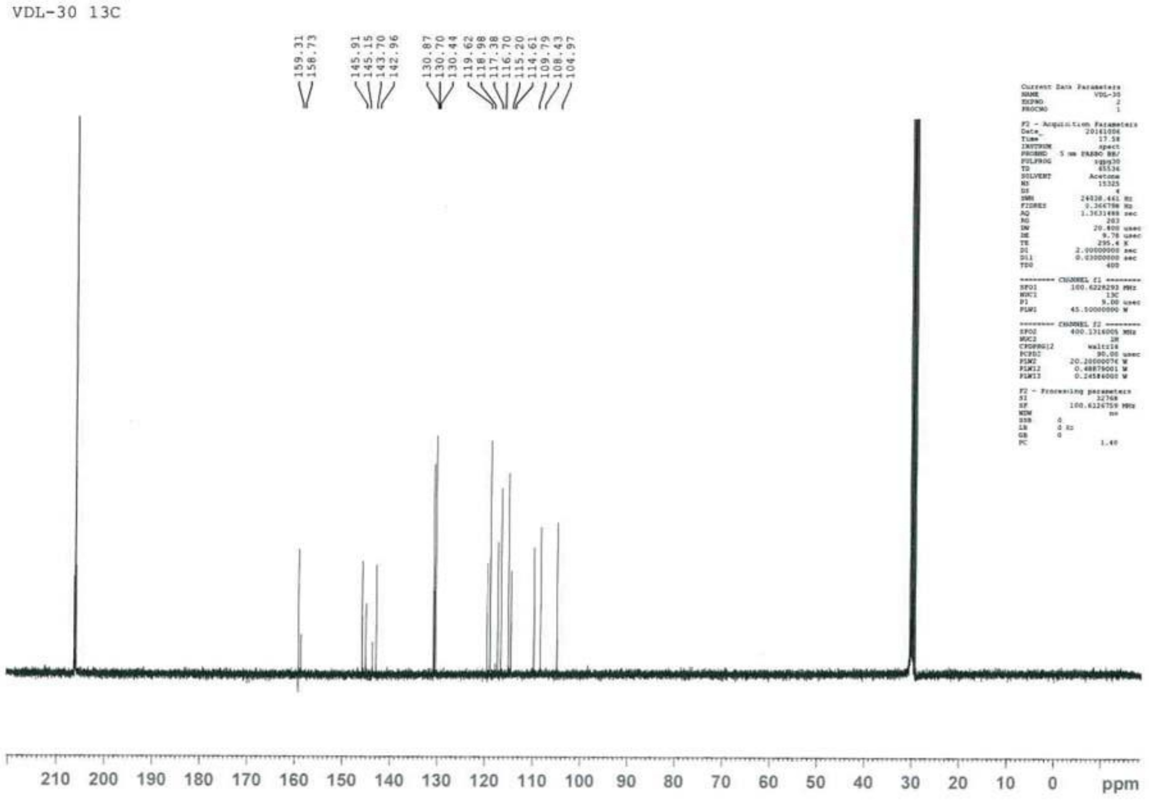

Figure S24. ^{1}H-NMR and ^{13}C-NMR of compound **13**.

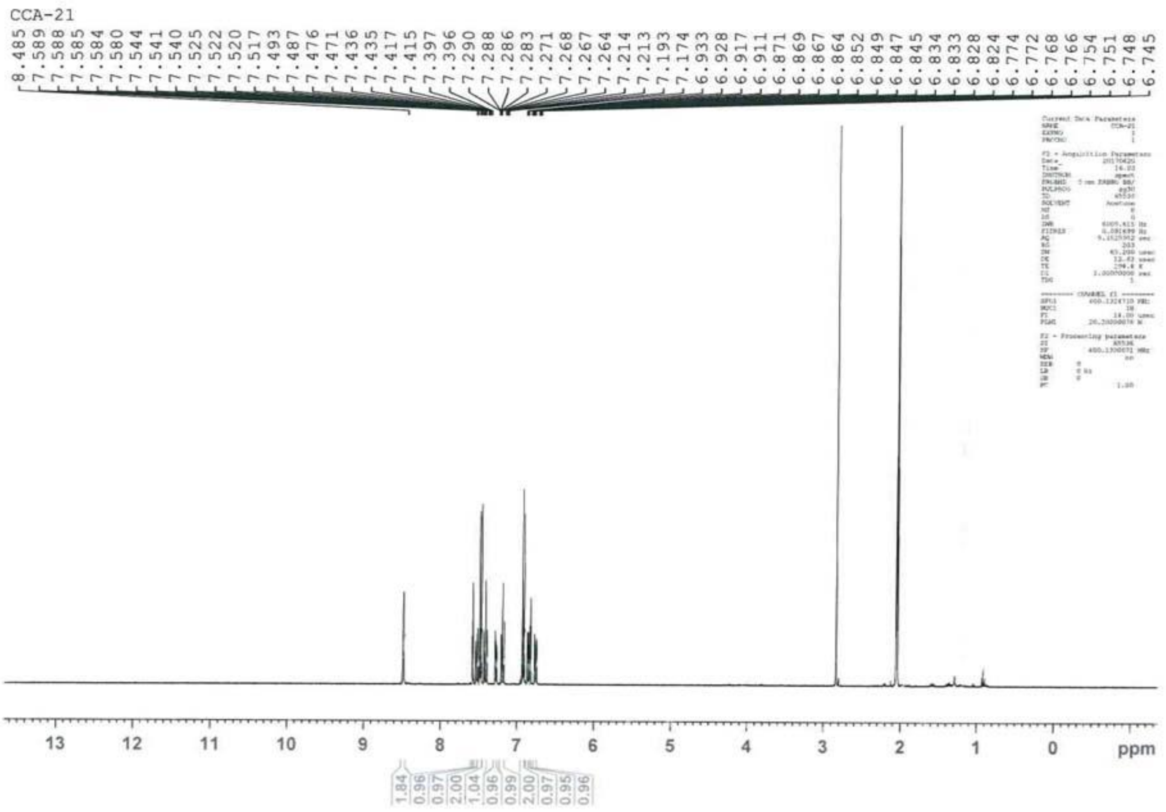

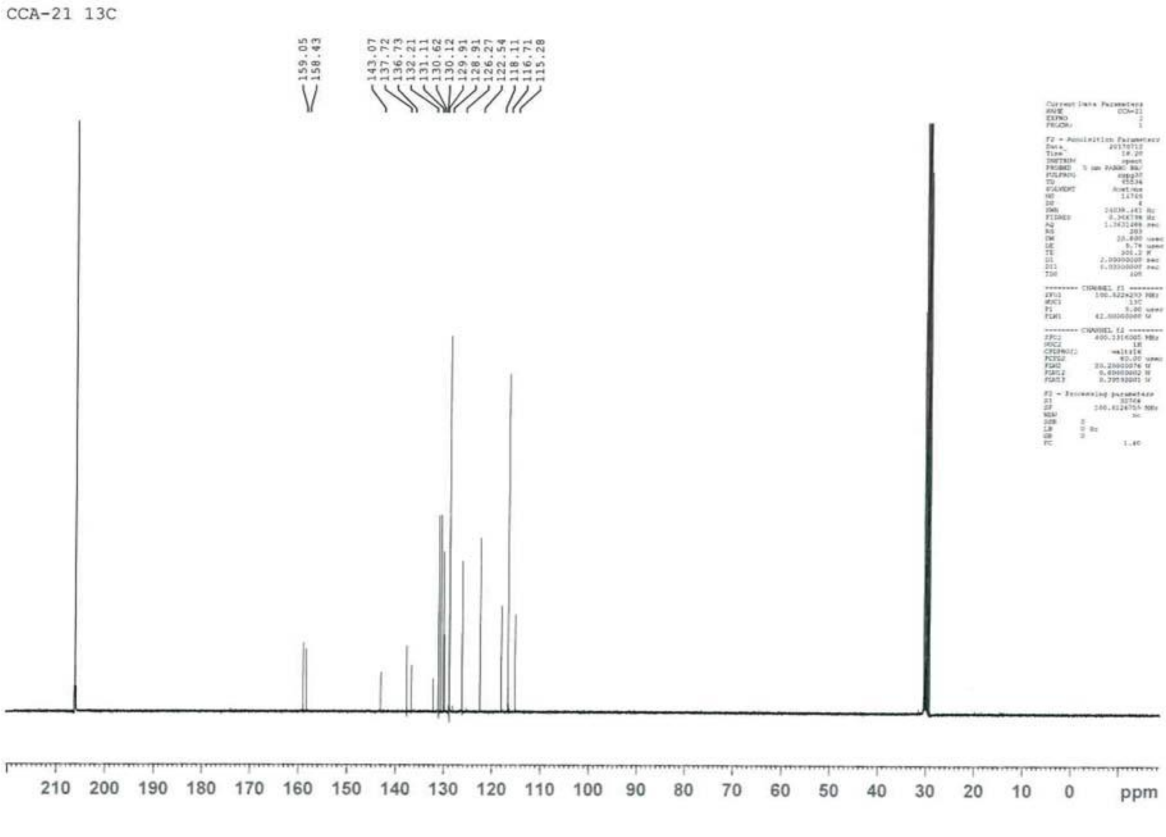

Figure S25. ^{1}H-NMR and ^{13}C-NMR of compound **14**.

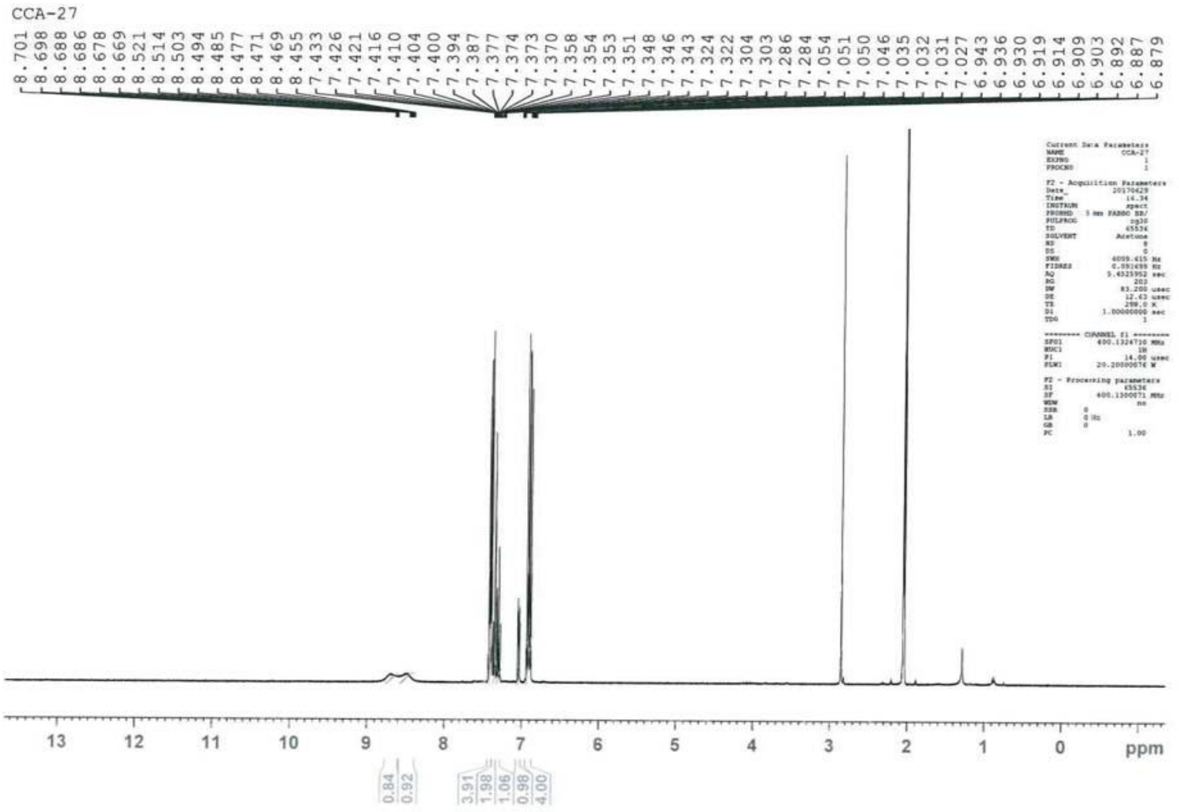

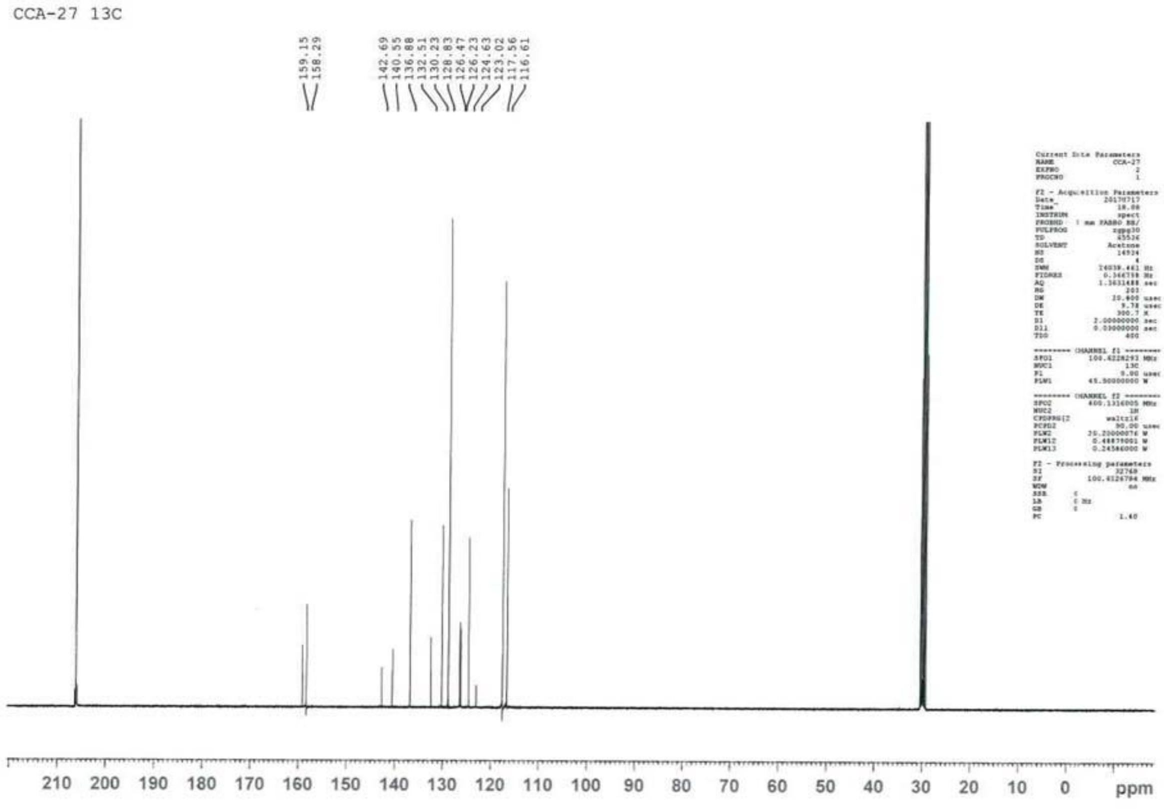

Figure S26. ^{1}H-NMR and ^{13}C-NMR of compound **15**.

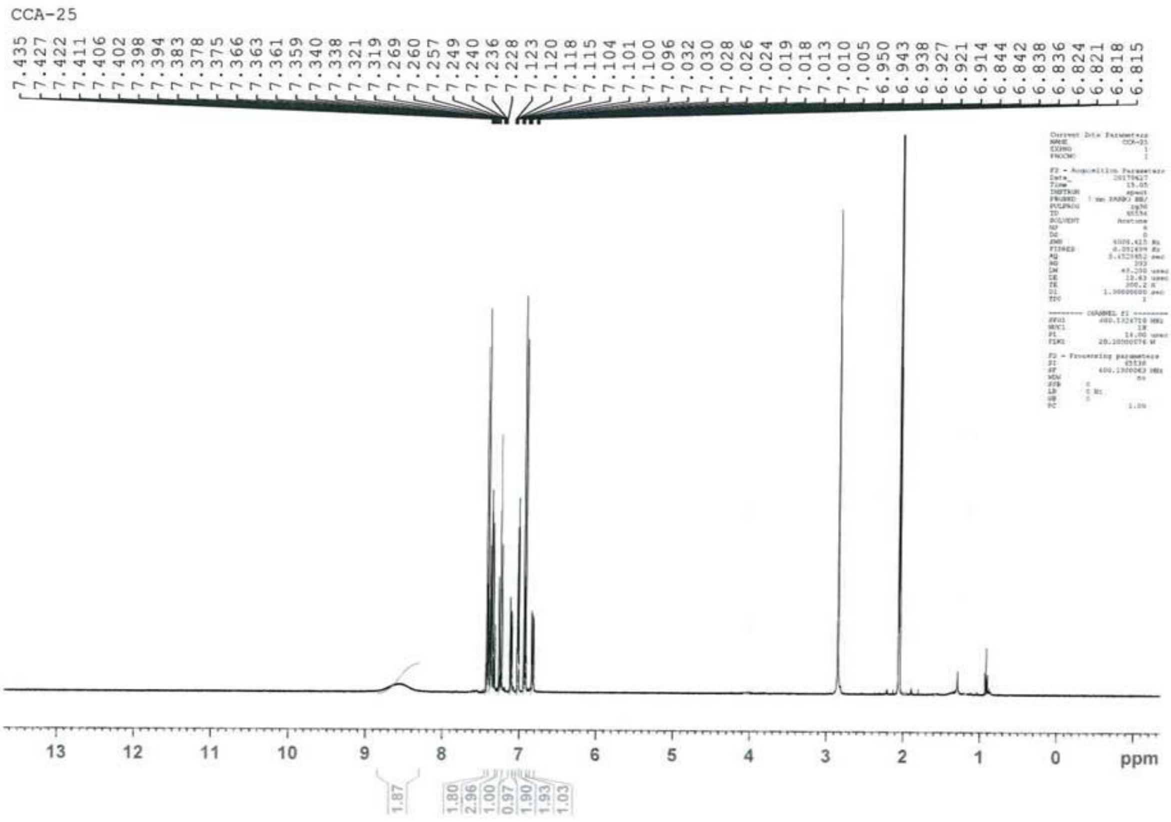

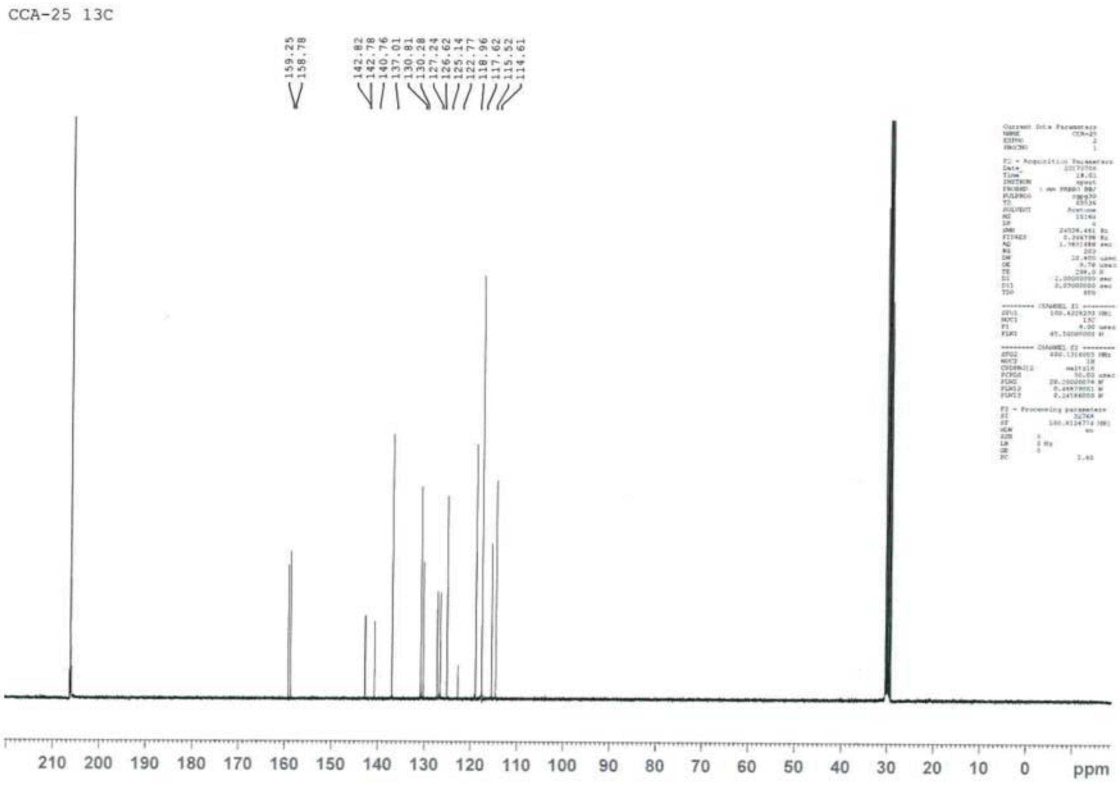

Figure S27. ^{1}H-NMR and ^{13}C-NMR of compound **16**.

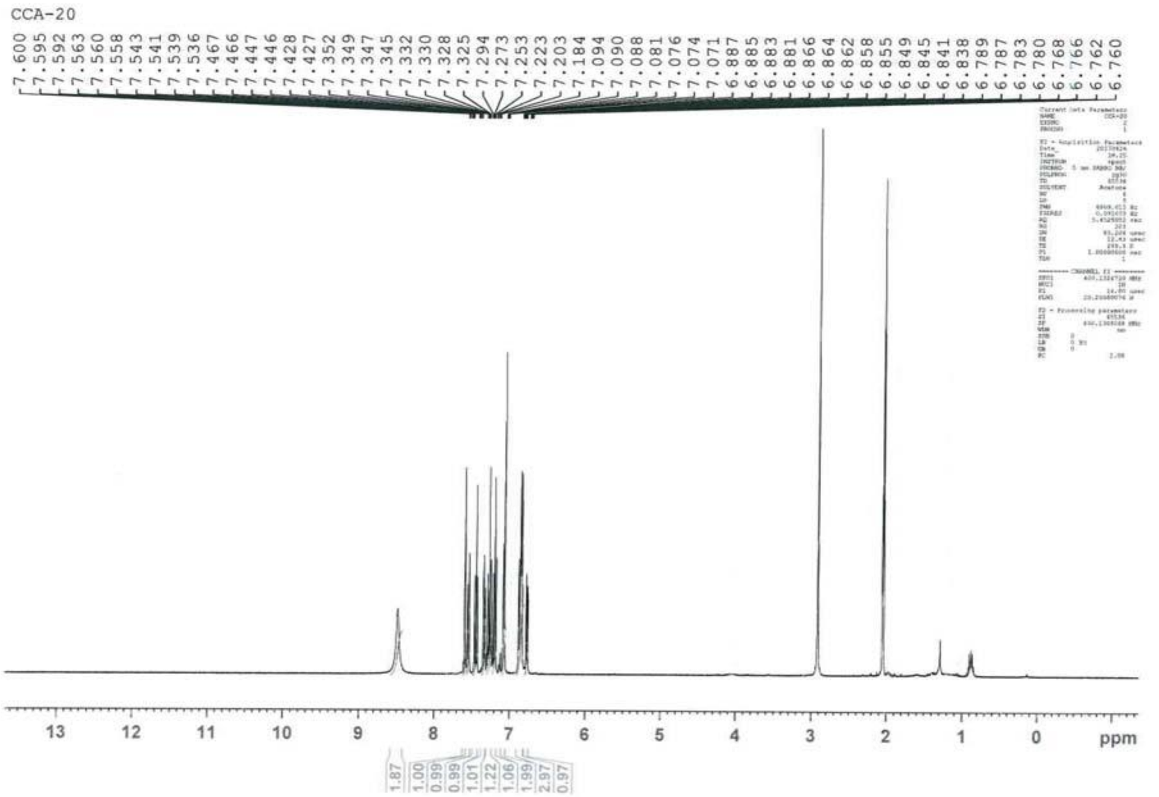

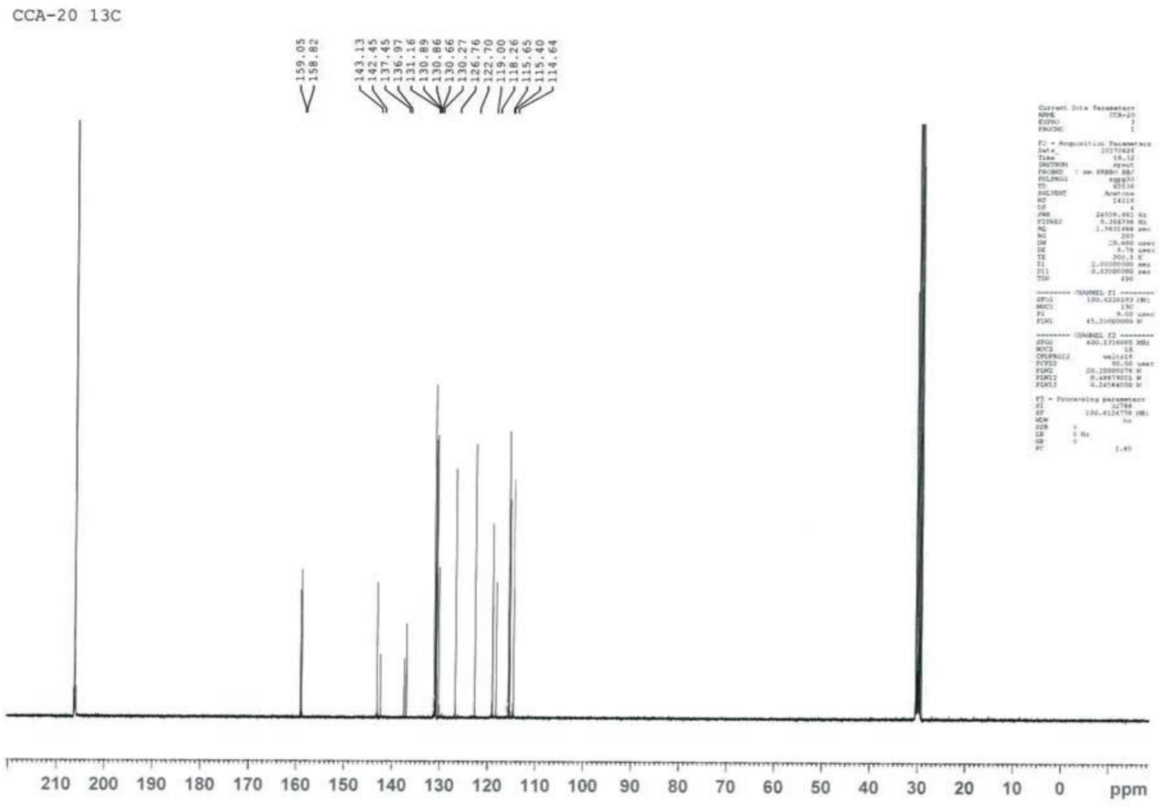

Figure S28. ^{1}H-NMR and ^{13}C-NMR of compound **17**.

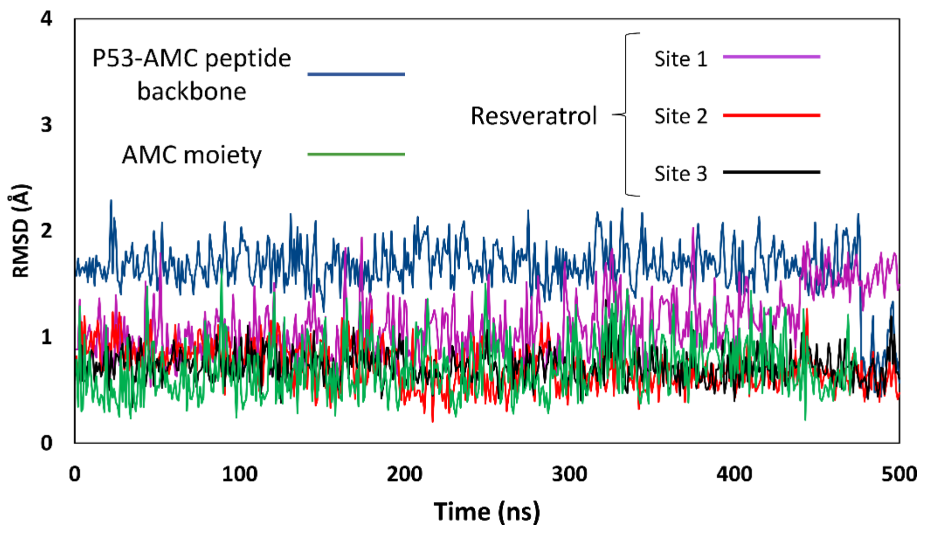

Figure S29. Results of the RMSD analysis of the MD simulation performed on the reference X-ray complex (PDB code 5BTR). The RMSD with respect to the crystallographic coordinates of the fluorogenic peptide backbone, the AMC moiety and the three resveratrol molecules are shown.

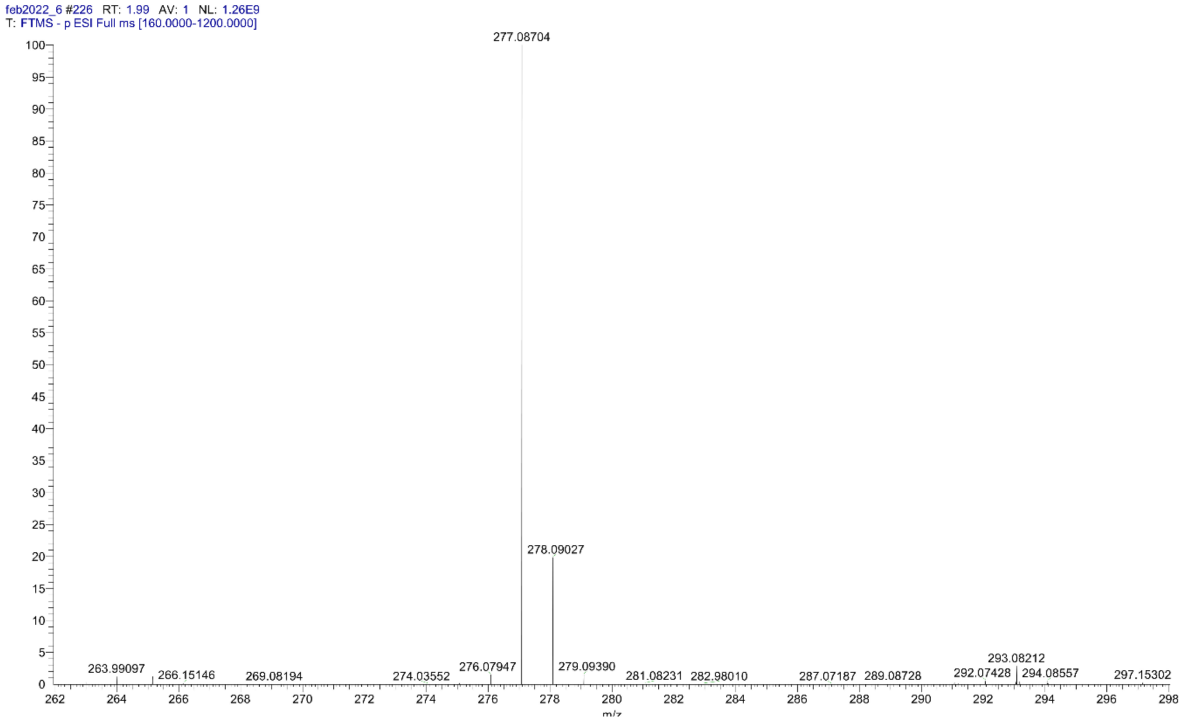

Figure S30. ESI-HRMS spectrum of compound **6**.

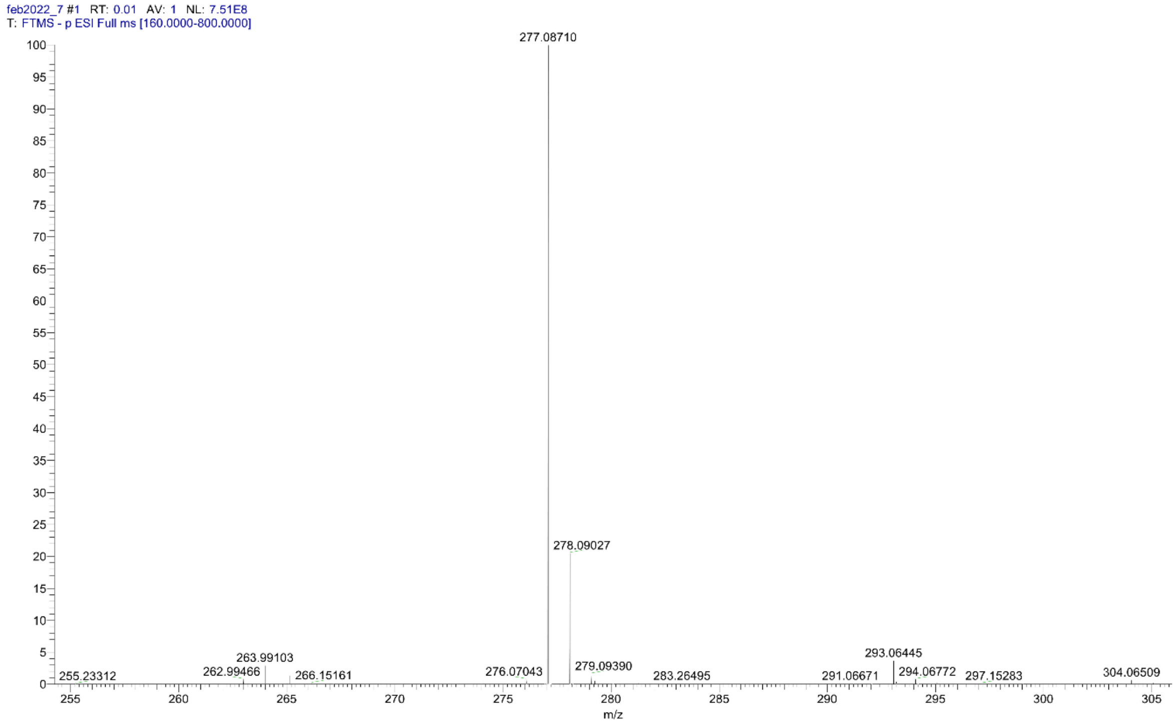

Figure S31. ESI-HRMS spectrum of compound **7**.

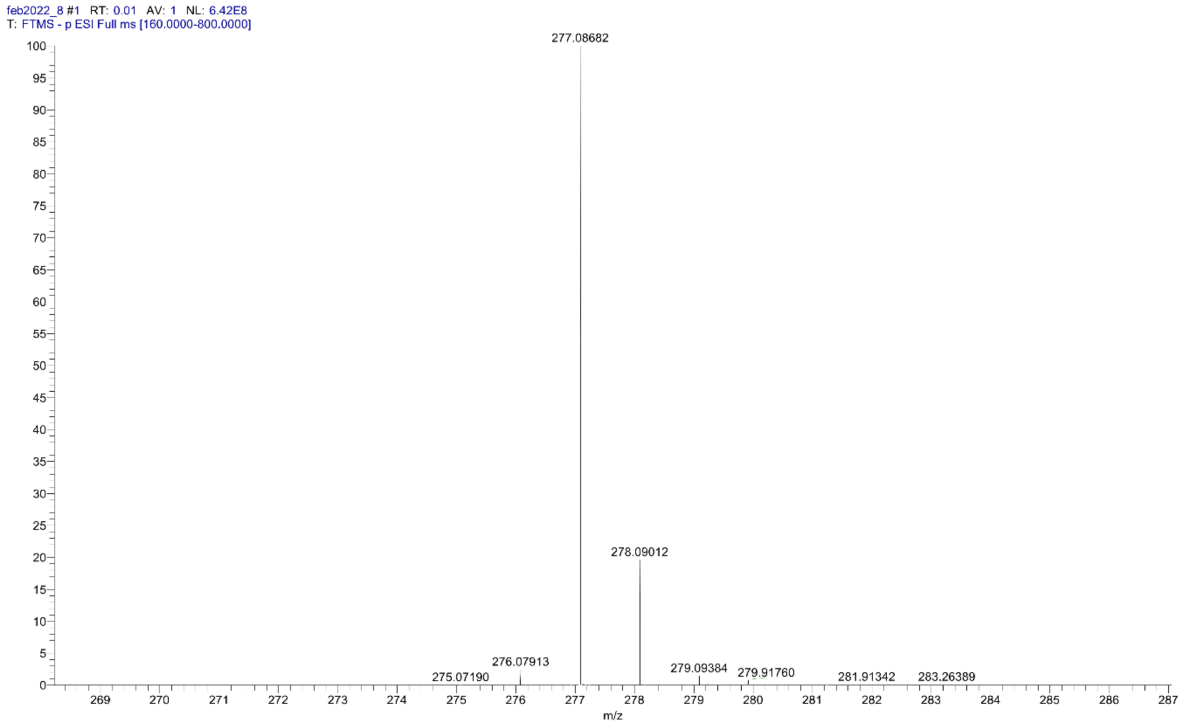

Figure S32. ESI-HRMS spectrum of compound **8**.

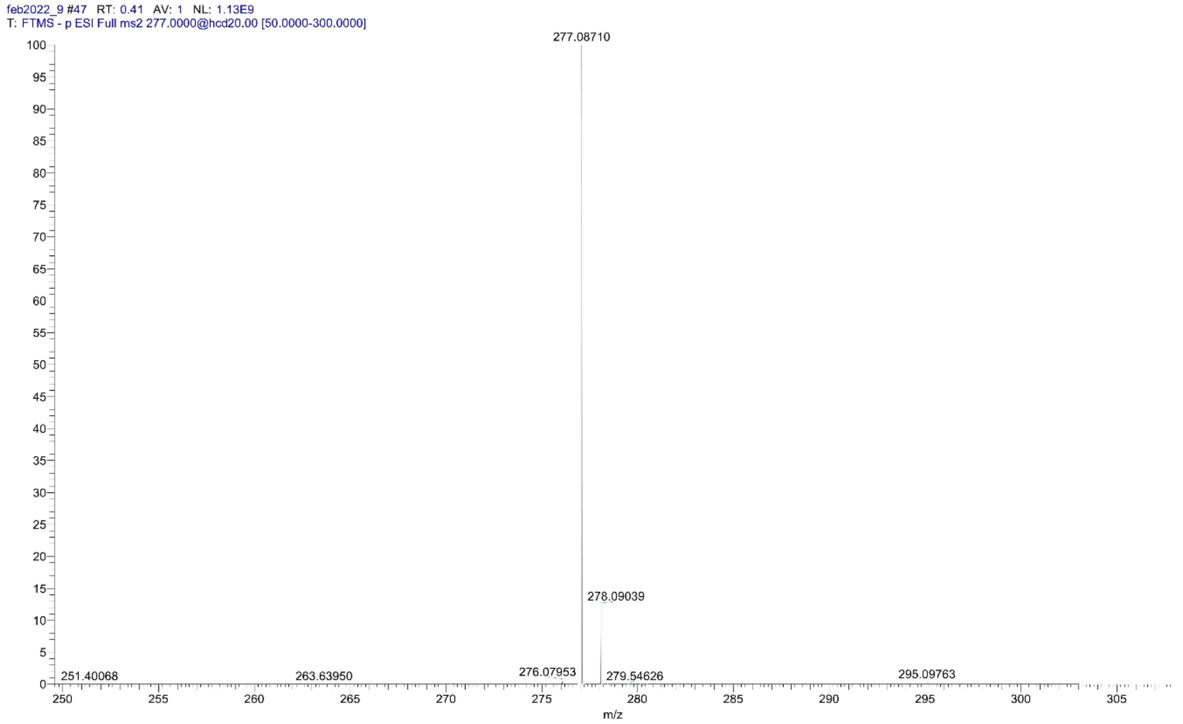

Figure S33. ESI-HRMS spectrum of compound **9**.

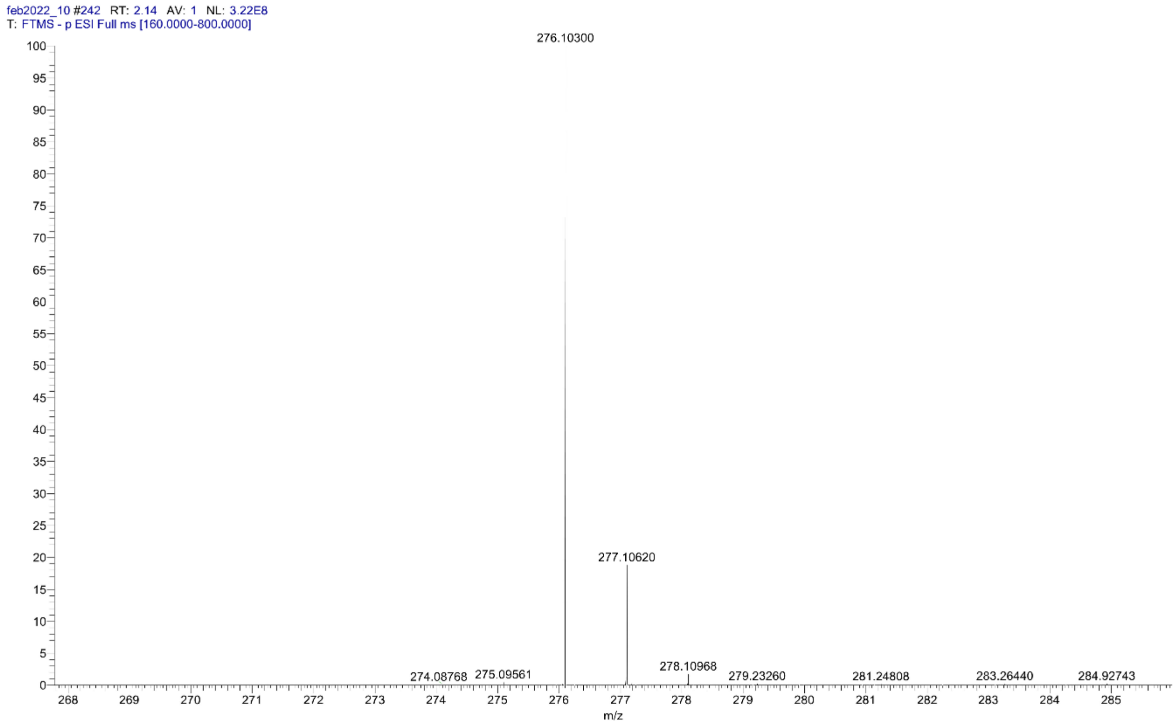

Figure S34. ESI-HRMS spectrum of compound **10**.

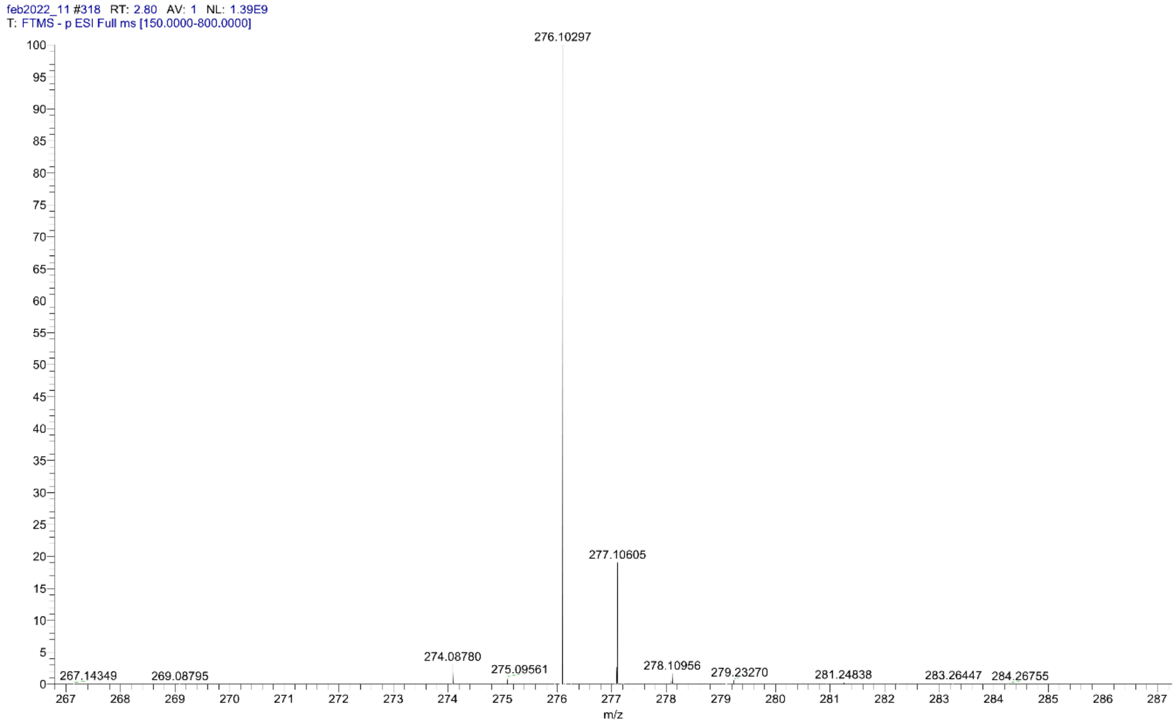

Figure S35. ESI-HRMS spectrum of compound **11**.

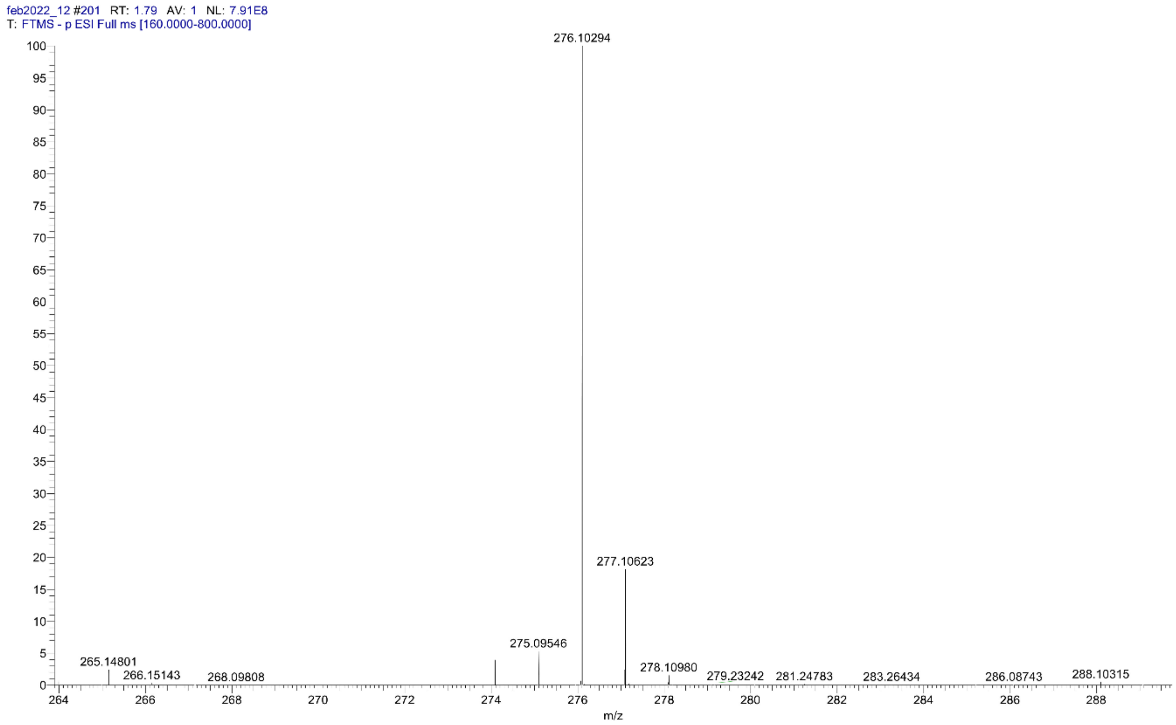

Figure S36. ESI-HRMS spectrum of compound **12**.

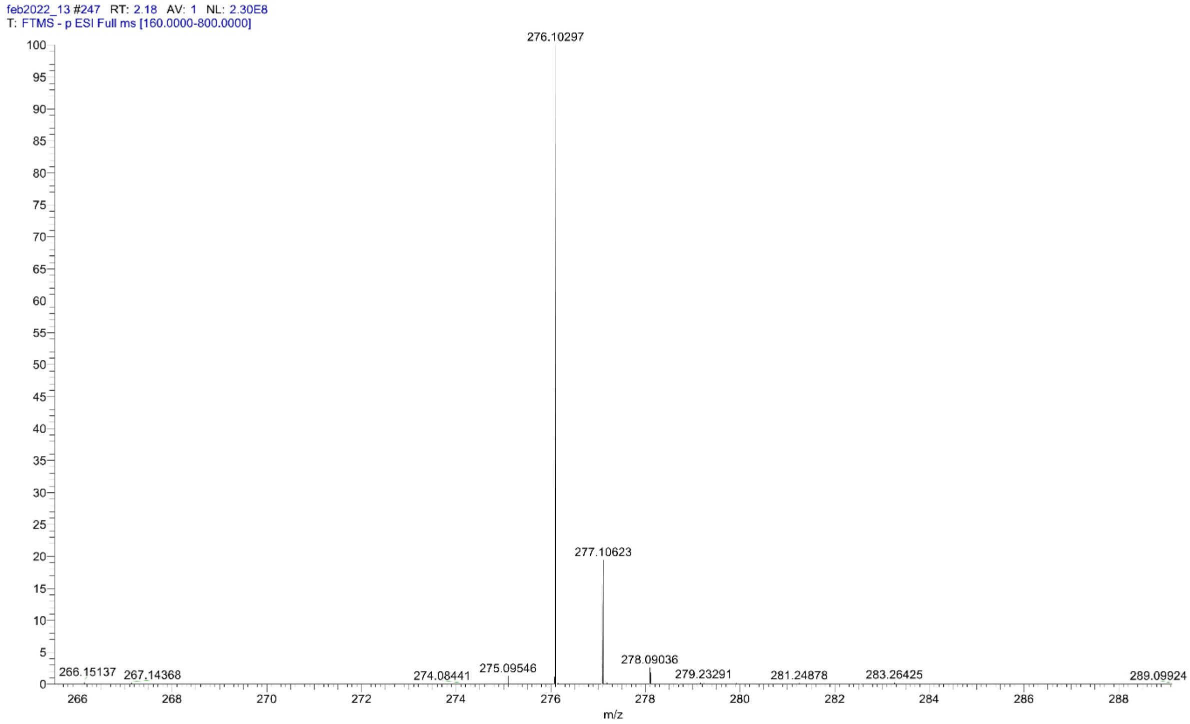

Figure S37. ESI-HRMS spectrum of compound **13**.

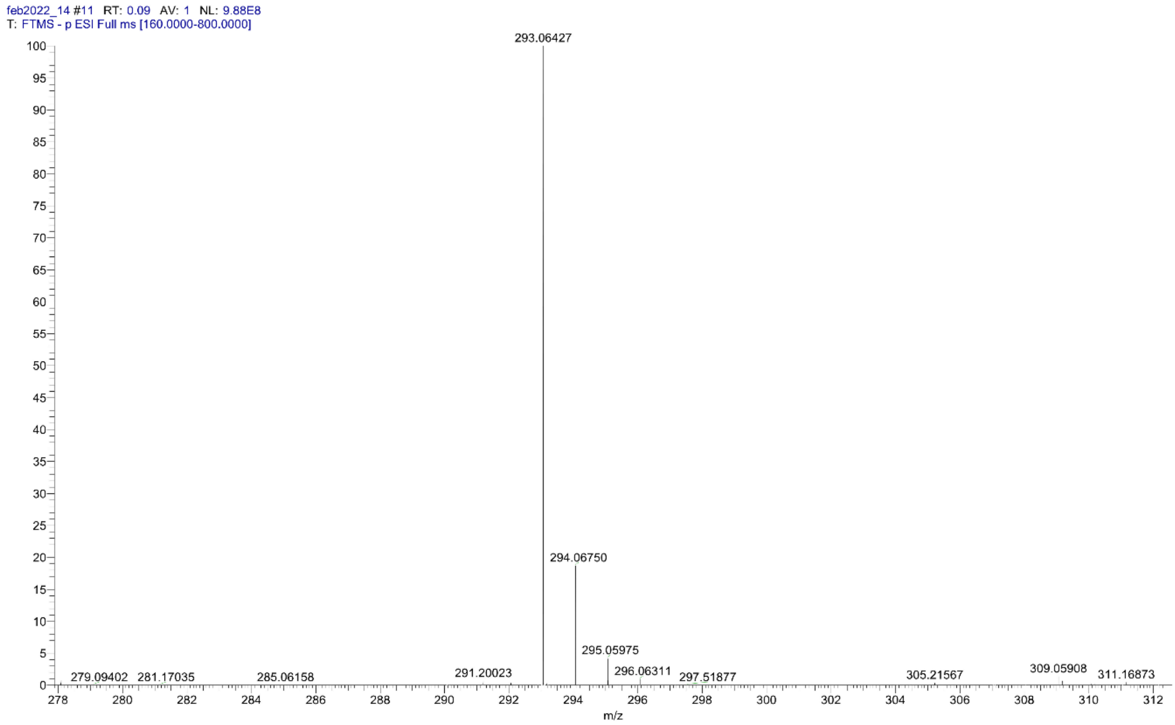

Figure S38. ESI-HRMS spectrum of compound **14**.

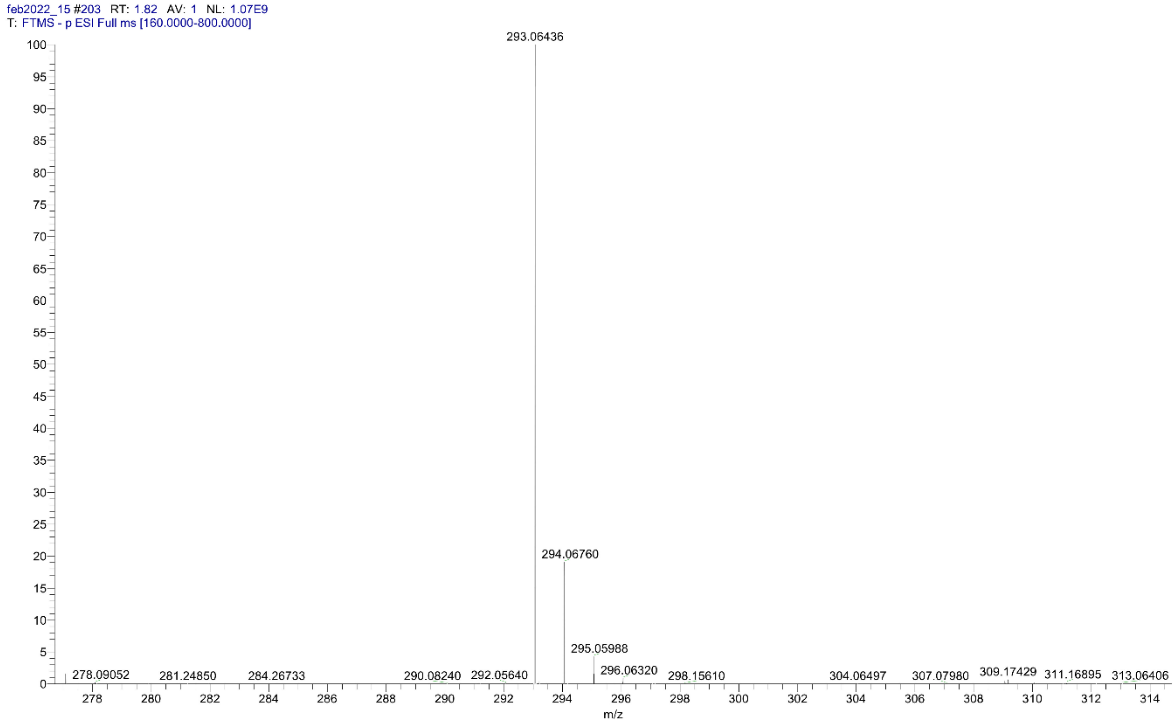

Figure S39. ESI-HRMS spectrum of compound **15**.

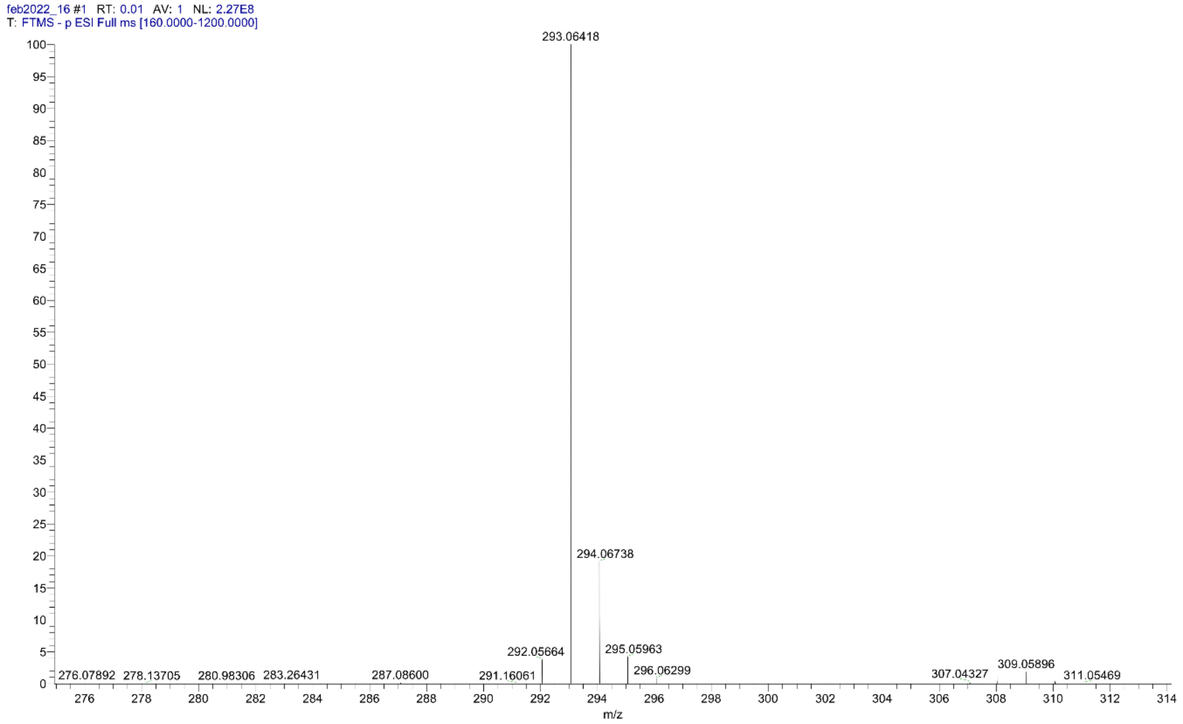

Figure S40. ESI-HRMS spectrum of compound **16**.

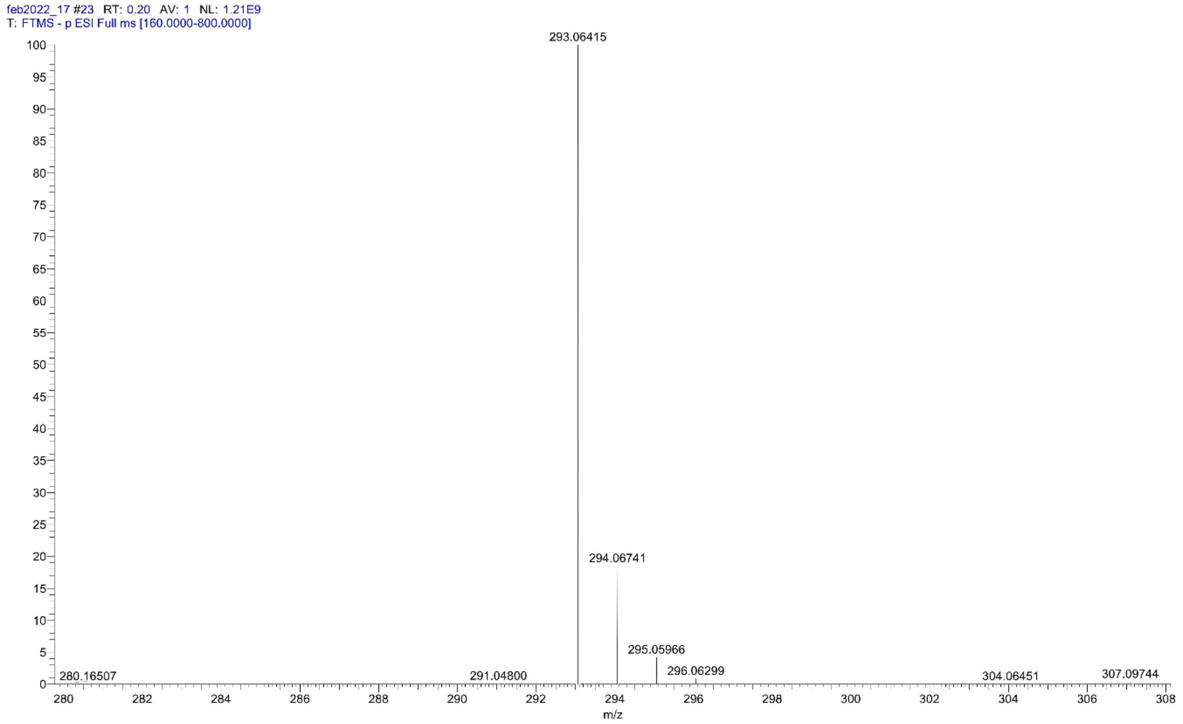

Figure S41. ESI-HRMS spectrum of compound **17**.

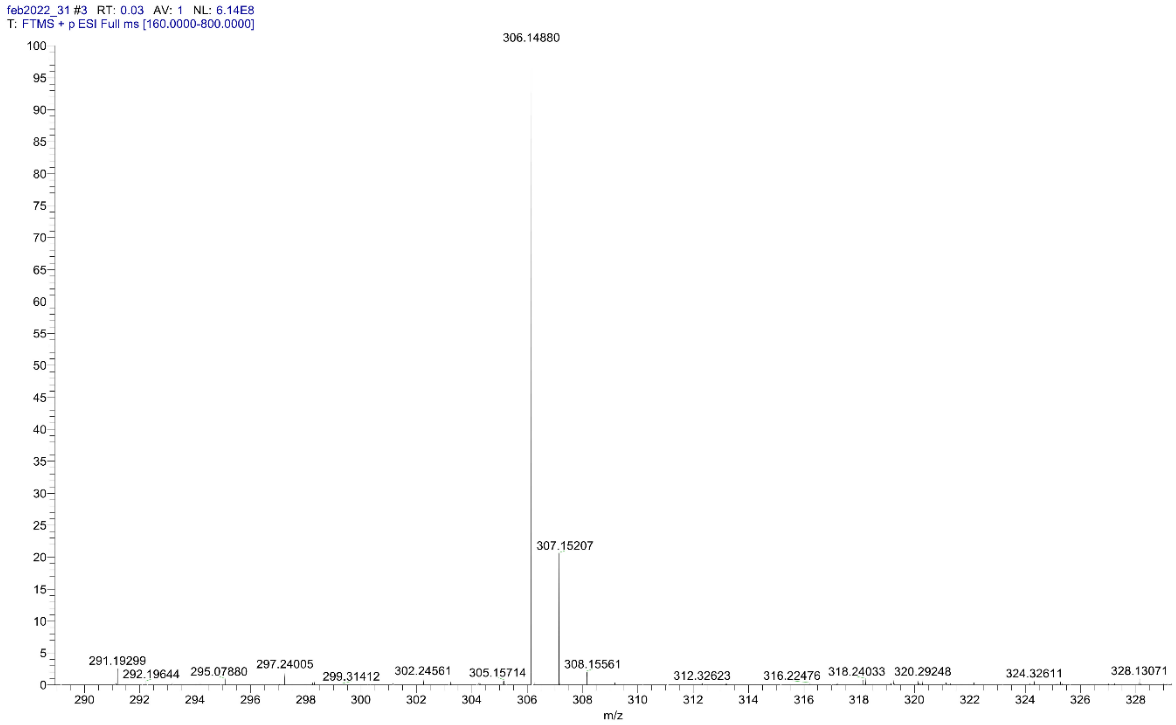

Figure S42. ESI-HRMS spectrum of compound **31**.

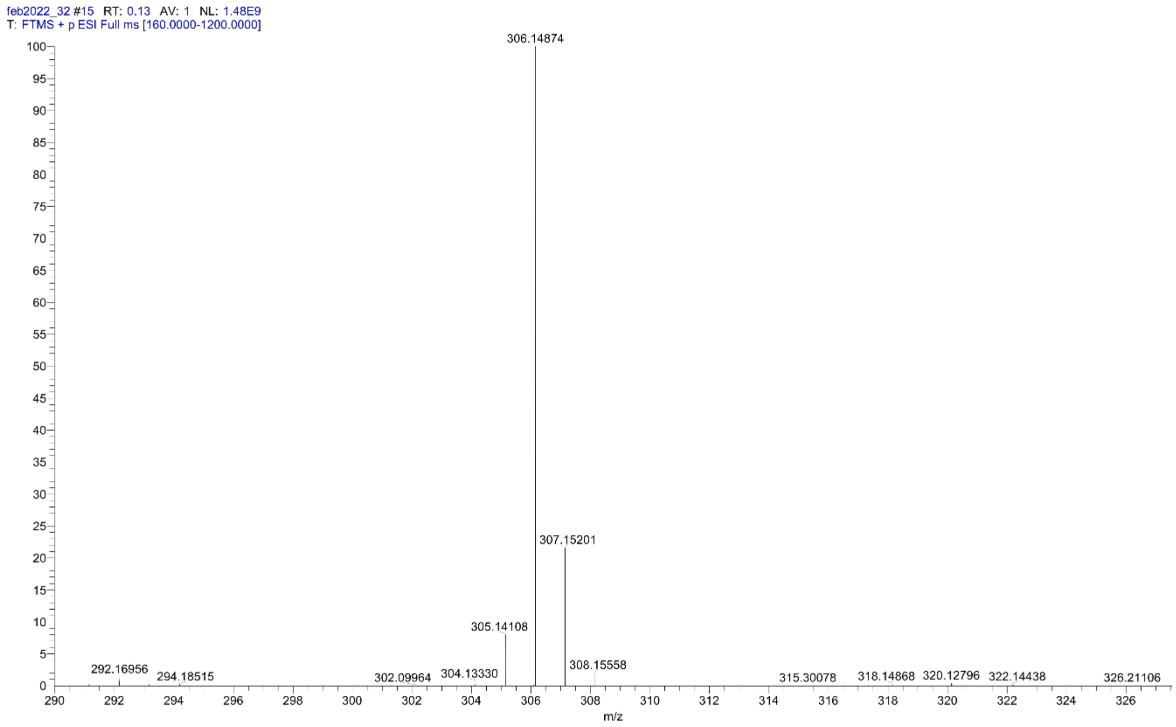

Figure S43. ESI-HRMS spectrum of compound **32**.
